# Crystal structures of *trans*-di­bromido­bis­(3,5-lutidine)gold(III) tribromide and three 3,5-lutidinium salts containing tetra­bromido­aurate(III) anions (including three cases of polymorphism)

**DOI:** 10.1107/S2056989025006401

**Published:** 2025-07-29

**Authors:** Cindy Döring, Peter G. Jones

**Affiliations:** a Institut für Anorganische und Analytische Chemie, Technische Universität Braunschweig, Hagenring 30, D-38106 Braunschweig, Germany; Universität Greifswald, Germany

**Keywords:** crystal structure, polymorphism, lutidine, tetra­bromido­aurate(III), hydrogen bond, halogen bond, coinage bond, stacking

## Abstract

*trans*-Di­bromido­bis­(3,5-lutidine)gold(III) tribromide, [AuBr_2_(C_7_H_9_N)_2_](Br_3_) or [(3,5-Lut)_2_AuBr_2_](Br_3_), **1**, polymorph **a**, crystallizes in the space group *P*1 with *Z* = 1. The gold atom and the central bromine of the tribromide ion lie on inversion centres. Polymorph **b** crystallizes in *C*222_1_ with *Z* = 4. The gold atom, the nitro­gen atoms and the ring atoms at the 4-position of the lutidine ligands, and the central bromine of the tribromide ion all lie on twofold axes. The formula units of **1a** and **1b** are closely similar (including the relative orientations of anion and cation). 3,5-Lutidinium tetra­bromido­aurate(III), (C_7_H_10_N)[AuBr_4_] or (3,5-LutH)[AuBr_4_], **2**, crystallizes as a new polymorph in *P*3_2_ with *Z* = 3. Bis(3,5-lutidinium) tetra­bromido­aurate(III) bromide, (C_7_H_10_N)_2_[AuBr_4_]Br or (3,5-LutH)_2_[AuBr_4_]Br, **3**, polymorph **a**, crystallizes in *C*2/*c* with *Z* = 4. The gold atom lies on an inversion centre and the bromide ion on a twofold axis. Polymorph **b** crystallizes in *P*2_1_/*c* with *Z* = 8 (*Z*′ = 2) and all atoms on general positions. Tris(3,5-lutidinium) bis­[tetra­bromido­aurate(III)] bromide, (C_7_H_10_N)_3_[AuBr_4_]_2_Br or (3,5-LutH)_3_[AuBr_4_]_2_Br, **4**, crystallizes in *P*1 with *Z* = 2. One gold atom lies on a general position and two on inversion centres. The main inter­est centres on the crystal packing patterns. In structure **1a**, a short Au⋯Br_anion_ contact, presumably a ‘coinage bond’, combines with a ‘weak’ hydrogen bond H_*ortho*_⋯Br_anion_ and a further contact Br_cation_⋯Br_anion_ to form a layer structure parallel to the *ac* plane. Polymorph **1b** shows similar Au⋯Br_anion_ and H_*ortho*_⋯Br_anion_ contacts, resulting in a chain of residues parallel to the *c* axis. The most striking feature of the previous polymorph of compound **2** was a topologically square, but distorted, network of tetra­bromido­aurate ions. The packing of the new polymorph of **2** involves three-centre hydrogen bonds Br⋯H⋯Br′, an axial coinage bond Au⋯Br, and two Br⋯Br contacts, one of which completes an unusual AuBr_2_ three-centre system. The extended packing shows three one-dimensional arrays of residues parallel to the threefold axis, linked by one Br⋯Br contact to form a layer. In polymorph **a** of compound **3**, the packing is based on a dimeric unit with twofold symmetry, centred on the free bromide ion, which is involved in two hydrogen bonds and two Br⋯Br inter­actions. The dimers are connected *via* further Br⋯Br contacts to form a zigzag chain parallel to the *c* axis. Polymorph **b** displays two hydrogen-bonded (3,5-LutH⋯)_2_Br groupings to the two free bromides, together with two tetra­bromido­aurate ions linked by a Br⋯Br contact; the second anion is also connected to a free bromide. The residues thus linked form a broad band parallel to the *c* axis. There are also infinite stacks of planar residues with the repeating sequence (⋯[AuBr_4_]^−^⋯lutidinium⋯lutidinium⋯) parallel to the *a* axis. In compound **4**, all three cations are hydrogen bonded to the free bromide. The anions at two gold centres (Au1/Au2) form a chain parallel to the *a* axis *via* Br⋯Br contacts. The anions at Au3 combine with the free bromide to form a chain of Au_2_Br_4_ rings parallel to the *a* axis, *via* Au⋯Br and Br⋯Br contacts, and these chains link with those at Au1 *via* another Br⋯Br contact to form a broad ribbon of residues. The ribbons are in turn linked by a Br⋯π contact.

## Abstract 2

Mol­ecular structures and packing diagrams of four systems involving 3,5-lutidine and gold(III) centres are analysed in terms of hydrogen bonds, halogen bonds, coinage bonds and stacking inter­actions.

## 1. Chemical context

In this series of publications, we have structurally investigated several classes of amine complexes of gold(I) and gold(III) halides, whereby the term ‘amine’ has been used loosely to include aza­aromatics. Background material is given in Parts 18 and (especially) 12 of this series (Döring & Jones, 2025 ▸, 2023 ▸).
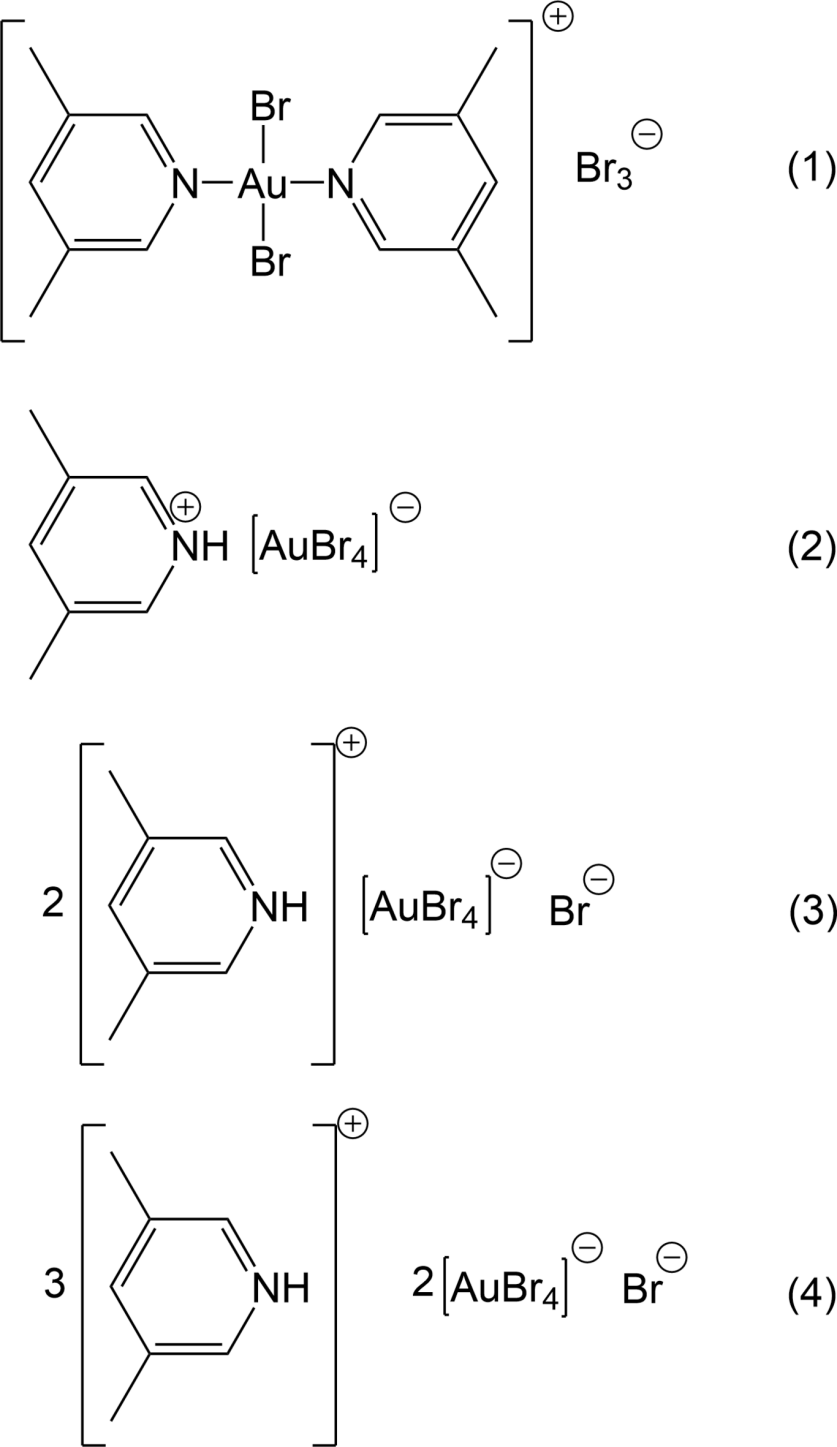


In the series of 3,5-di­methyl­pyridine (3,5-lutidine, henceforth abbreviated to ‘3,5-Lut’) complexes, we have previously determined the structures of (3,5-Lut)AuCl_3_ and (3,5-Lut)AuBr_3_, together with the 1:1 adduct of (3,5-Lut)AuBr_3_ with (2-picoline)AuBr_3_ (Döring & Jones, 2024*a* ▸) and the ionic gold(I) derivatives [(3,5-Lut)_2_Au][Au*X*_2_] (*X* = Cl and Br; Döring & Jones, 2024*b* ▸), which are isotypic. We have also presented the structures of three 3,5-lutidinium derivatives, namely (3,5-LutH)[AuBr_4_] (the previous polymorph of **2**), its diethyl ether solvate, and [(3,5-Lut)_2_H][AuBr_4_], in all of which the tetra­halogenidoaurate ions assembled to form approximately square networks with gold atoms at the corners and short bromine–bromine contacts Au—Br⋯Br—Au along the sides of the squares (Döring & Jones, 2016 ▸).

Here we present the structures of *trans*-di­bromido­bis­(3,5-lutidine)gold(III) tribromide, [(3,5-Lut)_2_AuBr_2_](Br_3_) **1** (as two polymorphs **1a** and **1b**), a second polymorph of 3,5-lutidinium tetra­bromido­aurate(III), (3,5-LutH)[AuBr_4_] **2**, bis­(3-5-lutidinium) tetra­bromido­aurate(III) bromide, (3,5-LutH)_2_[AuBr_4_]Br **3** (as two polymorphs **3a** and **3b**), and tris­(3,5-lutidinium) bis­[tetra­bromido­aurate(III)] bromide, (3,5-LutH)_3_[AuBr_4_]_2_Br **4**. As established for the 4-methyl­piperidinium derivatives in Part 18 (Döring & Jones, 2025 ▸), the presence of both halide and tetra­halogenidoaurate ions in **3** and **4** extends the potential types of observed contacts and substructures.

## 2. Structural commentary

**Figure 1:**
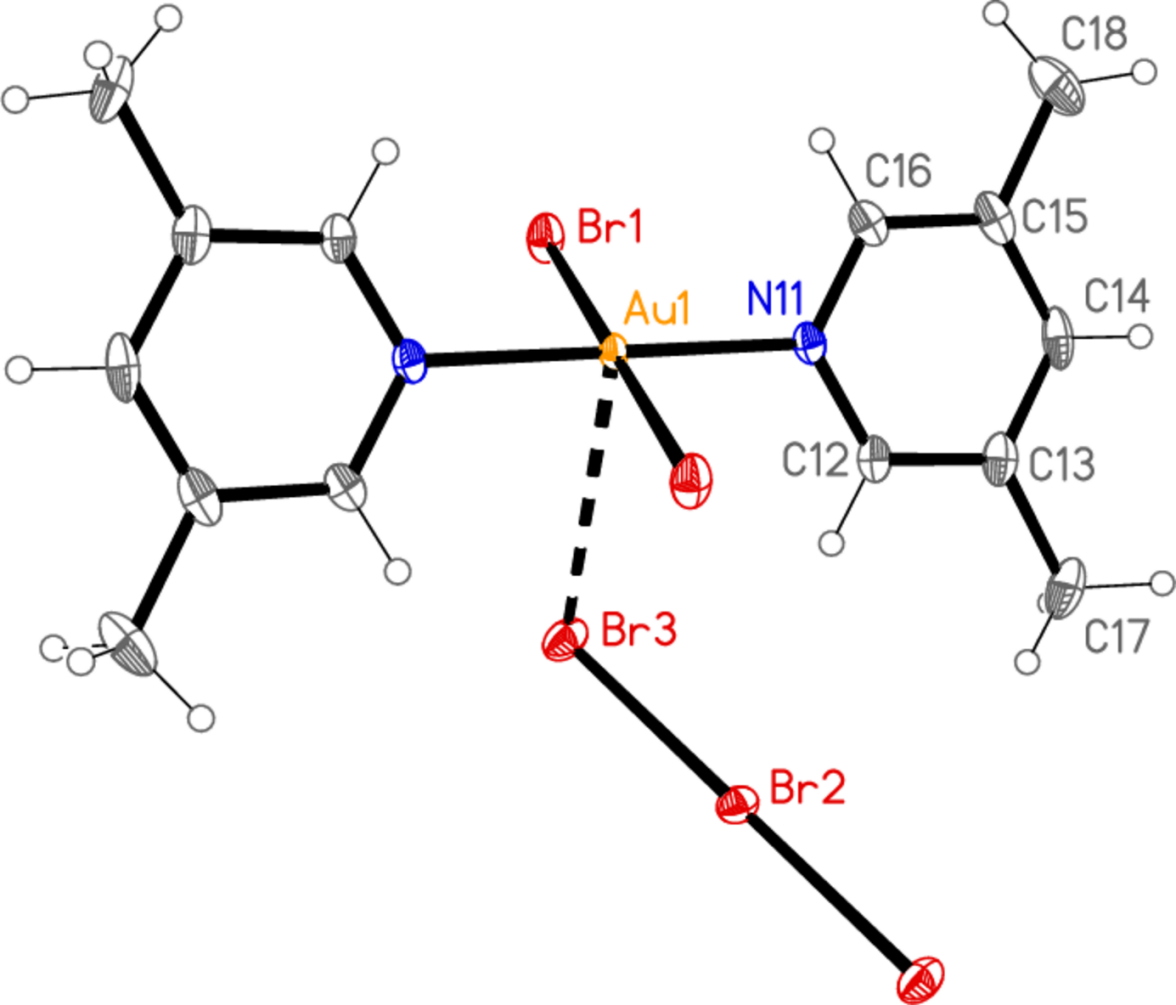
The formula unit of compound **1**, polymorph **1a**, in the crystal. Ellipsoids are drawn at the 50% probability level.

All compounds crystallize solvent-free. In the Figures (Figs. 1 ▸–6 ▸ ▸ ▸ ▸ ▸), the asymmetric units have been extended by symmetry where necessary to show complete residues; the dashed lines indicate short contacts, which are discussed in *Supra­molecular features*. Selected mol­ecular dimensions are shown in Tables 1 ▸–6 ▸ ▸ ▸ ▸ ▸.

Compound **1**, polymorph **1a**, crystallizes in *P*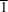 with *Z* = 1. The gold atom and the central bromine of the tribromide ion lie on inversion centres. Polymorph **1b** crystallizes in *C*222_1_ with *Z* = 4. The gold atom, the nitro­gen atoms and the ring atoms at the 4-position of the lutidine ligands, and the central bromine of the tribromide ion all lie on twofold axes. Compound **2** crystallizes in *P*3_2_ with *Z* = 3. Compound **3**, polymorph **3a**, crystallizes in *C*2/*c* with *Z* = 4. The gold atom lies on an inversion centre and the bromide ion on a twofold axis. Polymorph **3b** crystallizes in *P*2_1_/*c* with *Z* = 8 (*Z*′ = 2) and all atoms on general positions. Compound **4** crystallizes in *P*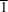 with *Z* = 2. One gold atom lies on a general position and two on inversion centres.

The tetra­bromido­aurate(III) ions show the expected square-planar (4/*mmm*) symmetry to a good approximation, although there is some scatter of the Au—Br bond lengths, which range from 2.4046 (8) to 2.4300 (5) Å. The *cis* angles at gold are all within 1.1° of the ideal 90°, and the maximum deviation for the *trans* angles is 2.4°. The C—N—C angles of the lutidinium cations lie in the narrow range 123–124°.

The formula units of the polymorphs **1a** and **1b** are closely similar, despite the difference in formal symmetry. The Au—N bonds are short, as is usual for mutually *trans* Au—N bonds at Au^III^ centres. The C—N—C angles of the lutidine ligands are 120.6 (2)° for **1a** and 120.5 (5), 121.5 (5)° for **1b**. The angle between the lutidine ring planes is 0° for **1a** (by symmetry) and 5.1 (4)° for **1b**. Even the relative orientations of the cation and the anion are similar, with N—Au⋯Br—Br torsion angles of −129.79 (2) and 50.21 (2)° for **1a** and −120.71 (6) and 59.29 (6) for **1b** (see also *Supra­molecular features*).

## 3. Supra­molecular features

In the packing diagrams, atom labels indicate atoms of the asymmetric unit; hydrogen atoms of the ring CH groups are generally omitted unless relevant to the packing. We subjectively assess the C—H⋯Br contacts to be less important than N—H⋯Br. In the text, primes (′) indicate previously defined or generalized symmetry operators. Hydrogen bonds are listed in Tables 7 ▸–12 ▸ ▸ ▸ ▸ ▸.

In compound **1**, polymorph **1a**, within the formula unit (the asymmetric unit plus, if necessary, atoms generated by symmetry to form complete residues), a short Au1⋯Br3 contact of 3.4502 (3) Å, axial with respect to the ligand plane, connects the anion and cation, and may be considered a ‘coinage bond’ (Daolio *et al.*, 2021 ▸; Pizzi *et al.*, 2022 ▸). The contact angles are Au1⋯Br3—Br2 102.48 (1) and Br1—Au1⋯Br3 87.03 (1)°. The ‘weak’ hydrogen bond H12⋯Br2, also within the formula unit but not drawn explicitly in Fig. 1 ▸, is by far the shortest H⋯Br contact at 2.79 Å. The Br1⋯Br3(−*x*, 1 − *y*, 1 − *z*) contact of 3.4995 (4) Å, with angles Au1—Br1⋯Br3′ = 162.48 (1) and Br2—Br3⋯Br1′ = 135.46 (1)°, is presumably a halogen bond [see *e.g.* Metrangolo *et al.* (2008 ▸) or Cavallo *et al.* (2016 ▸)]. These three contacts combine to form a layer structure parallel to the *ac* plane (Fig. 7 ▸). The packing of the second polymorph **1b** shows a similar contact within the formula unit to that of **1a**, namely Au1⋯Br3 = 3.5486 (5) Å, with Au1⋯Br3—Br2 = 108.18 (2) and Br1—Au1⋯Br3 = 87.20 (1)°. There is also a short intra­cationic contact H22⋯Br2′ of 2.88 Å; Br2 accepts two equivalent such hydrogen bonds with an H⋯Br⋯H angle of 86°. However, there are no contacts of the type Br_anion_⋯Br_cation_; the shortest Br⋯Br contact is Br1⋯Br1(*x*, 1 − *y*, 2 − *z*) = 3.8024 (9) Å, with Au1—Br1⋯Br1′ = 125.38 (2)°, which in terms of length is at best a borderline contact. The contacts combine to form chains of residues parallel to the *c* axis (Fig. 8 ▸).

Compound **2** is already known as a triclinic polymorph (*P*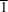, *Z* = 2) with a topologically square, but distorted, network of tetra­bromido­aurate ions involving Au atoms at the corners and Au—Br⋯Br—Au units along the edges (Döring & Jones, 2016 ▸). The packing diagram as originally published stressed this network and deliberately excluded the cations, which are hydrogen bonded to bromide ligands. We remedy this deficiency here (Fig. 9 ▸). The packing of the new polymorph is entirely different; it involves three-centre hydrogen bonds Br1⋯H01⋯Br1′ with an angle of 87 (2)° at the hydrogen atom and an axial coinage bond Au1⋯Br1(1 − *x* + *y*, 2 − *x*, 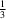 + *z*) of 3.5023 (9) Å, in addition to the halogen bonds Br4⋯Br1(1 − *x* + *y*, 2 − *x*, 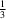 + *z*) = 3.6158 (13) Å (which completes an unusual three-centre inter­action between Au1, Br4 and Br1′) and Br2⋯Br3(2 − *y*, 2 + *x* − *y*, −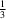 + *z*) = 3.4290 (13) Å. To simplify the packing diagrams, the rather longer contact Br3⋯Br4(1 − *y*, 1 + *x* − *y*, −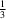 + *z*) = 3.8901 (14) Å has been omitted. The associated, mostly approximately linear, angles are: Au1—Br2⋯Br3′ = 170.65 (4), Au1—Br3⋯Br2′ = 163.68 (5), Au1—Br1⋯Br4′ = 167.75 (4) and Au1—Br1⋯Au1′ = 137.24 (3)°, with approximate right angles for *e.g.* Br1—Au1⋯Br1′, 82.32 (2)°. Within the three-centre triangle, the angles are 67.55 (3), 72.59 (4) and 39.86 (2)°, respectively, at Au1, Br4 and Br1′. A packing diagram viewed perpendicular to the *ac* plane (Fig. 10 ▸) shows three one-dimensional arrays of residues parallel to the threefold axis (horizontal), linked by Br2⋯Br3′ to form a layer. A further Br2⋯Br3′ contact links the layers thus formed in the third dimension (Fig. 11 ▸).

**Figure 5:**
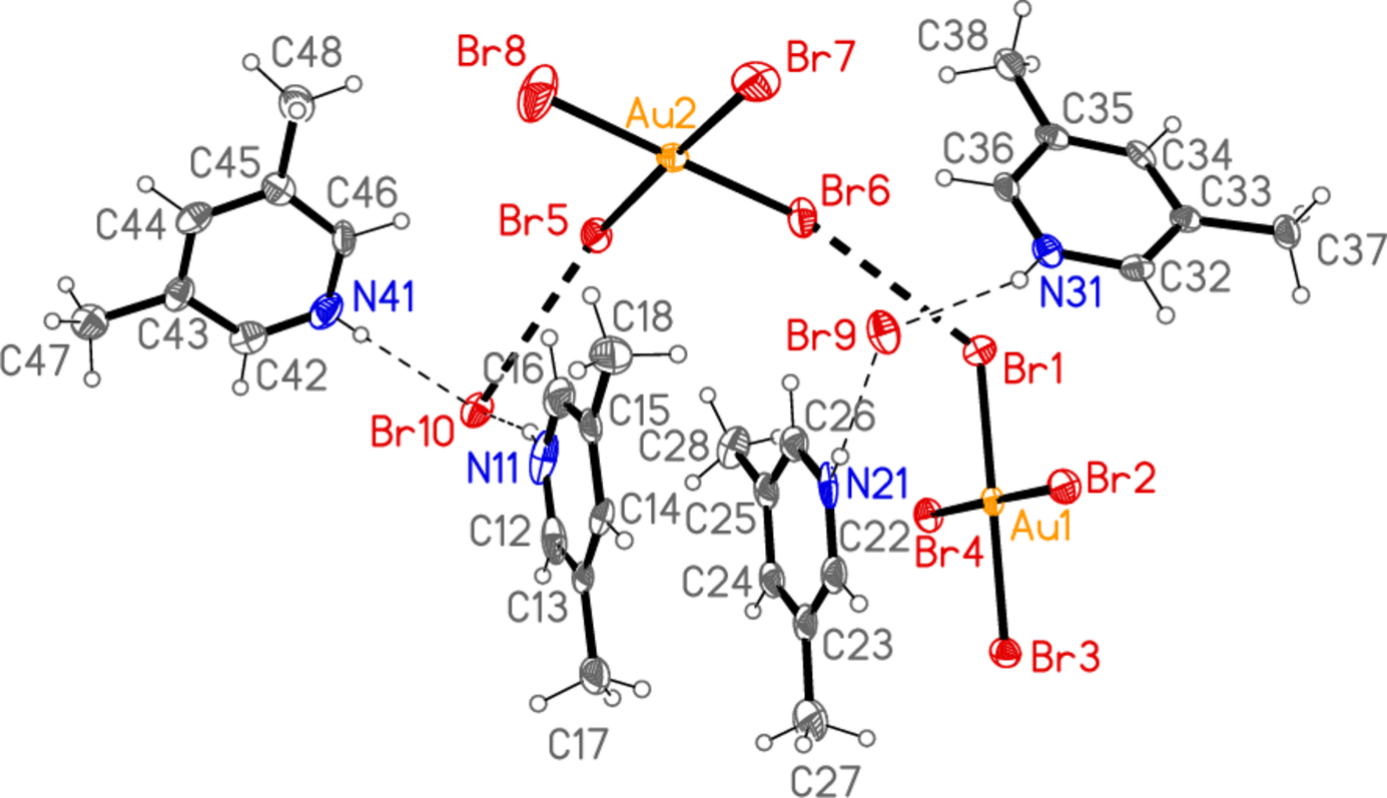
The asymmetric unit of compound **3**, polymorph **3b**, in the crystal. Ellipsoids are drawn at the 50% probability level.

In polymorph **a** of compound **3**, the most obvious substructure in the packing is a dimeric unit with twofold symmetry (Fig. 12 ▸) centred on the free bromide ion Br3, with contacts Br2⋯Br3 of 3.6097 (6) Å that augment the classical hydrogen bonds. The free bromide is thus involved in two hydrogen bonds and two Br⋯Br inter­actions. The relevant angles are Au1—Br2⋯Br3 = 172.24 (2), Br2⋯Br3⋯Br2(1 − *x*, *y*, 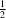 − *z*) = 78.52 (2) and H01⋯Br3⋯H01′ = 106 (2)°. Dimers are connected *via* further Br2⋯Br3 contacts to form a zigzag chain parallel to the *c* axis (Fig. 13 ▸). Polymorph **b** is more complex, with eight residues in the asymmetric unit (Fig. 5 ▸), which includes two hydrogen-bonded (3,5-LutH⋯)_2_Br groupings to the free bromides Br9 and Br10, together with two tetra­bromido­aurate ions linked by the contact Br1⋯Br6; the second anion is also connected to a free bromide by the contact Br5⋯Br10. In contrast to **3a**, the free bromide Br9 is thus involved in two hydrogen bonds only, whereas Br10 is involved in two hydrogen bonds and one Br⋯Br contact. Associated dimensions for the hydrogen bonded units are H02⋯Br9⋯H03 = 98 (2) and H02⋯Br10⋯H03 = 87 (2)°, and for the anions Br1⋯Br6 = 3.6451 (11), Br5⋯Br10 = 3.7036 (11) Å, Au1—Br1⋯Br6 = 127.16 (3), Br1⋯Br6—Au2 = 164.26 (4) and Au2—Br5⋯Br10 = 151.71 (3). A further contact between the anions is Br2⋯Br4(*x*, 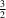 − *y*, −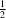 + *z*) of 3.6543 (10) Å, with Au1—Br2⋯Br4′ = 152.57 (3) and Au1—Br4⋯Br2′ = 151.02 (3)°. The residues are thus linked to form a broad band (with width equal to the *a* axis length) parallel to *c* (Fig. 14 ▸).

The packing of **3b** as seen in Fig. 14 ▸ seems to be comprehensible, if complicated. It would thus not be suspected that a whole class of packing inter­actions has not been shown, but this is indeed the case; inter­actions involving the aromatic rings have not yet been considered. For aromatic ring systems, stacking (π–π), C—H⋯π or halogen⋯π contacts may be present. These are not observed for structures **1**–**3a**, but for **3b** they become important. We use the following notation: ring *n* is the ring containing N*n*1, and *Cgn* = centroid of ring *n*. Rings 1 and 2 align in parallel, with an inter­planar angle of 2.85 (6)°, a *Cg*1⋯*Cg*2 distance of 3.637 (4) Å and an offset of 1.15 Å. Furthermore, these rings stack with neighbouring tetra­bromido­aurate anions involving Au1, with Au1⋯*Cg*2 = 3.588 (3), Au1(−1 + *x*, *y*, *z*)⋯*Cg*1 =3.566 (3) Å, respective inter­planar angles of 2.69 (5) and 1.17 (3)° and respective offsets 0.51, 0.65 Å. The resulting pattern consists of infinite stacks with the repeating sequence (⋯[AuBr_4_]^−^⋯lutidinium⋯lutidinium⋯) parallel to the *a* axis (Fig. 15 ▸). Angles along the stack are *Cg*1′⋯Au1⋯*Cg*2 = 173°, *Cg*2⋯*Cg*1⋯Au1′ = 156°, *Cg1*⋯*Cg2*⋯Au1 = 156°. Finally, Br7 is involved in a short Br⋯π contact of 3.313 (3) Å to *Cg*3(1 − *x*, 1 − *y*, 1 − *z*); this connects the region *y* ≃ 0.75 (as in Figs. 14 ▸ and 15 ▸) with that at *y* ≃ 0.25 (Fig. 16 ▸). Such contacts can be regarded as a type of halogen bond.

**Figure 6:**
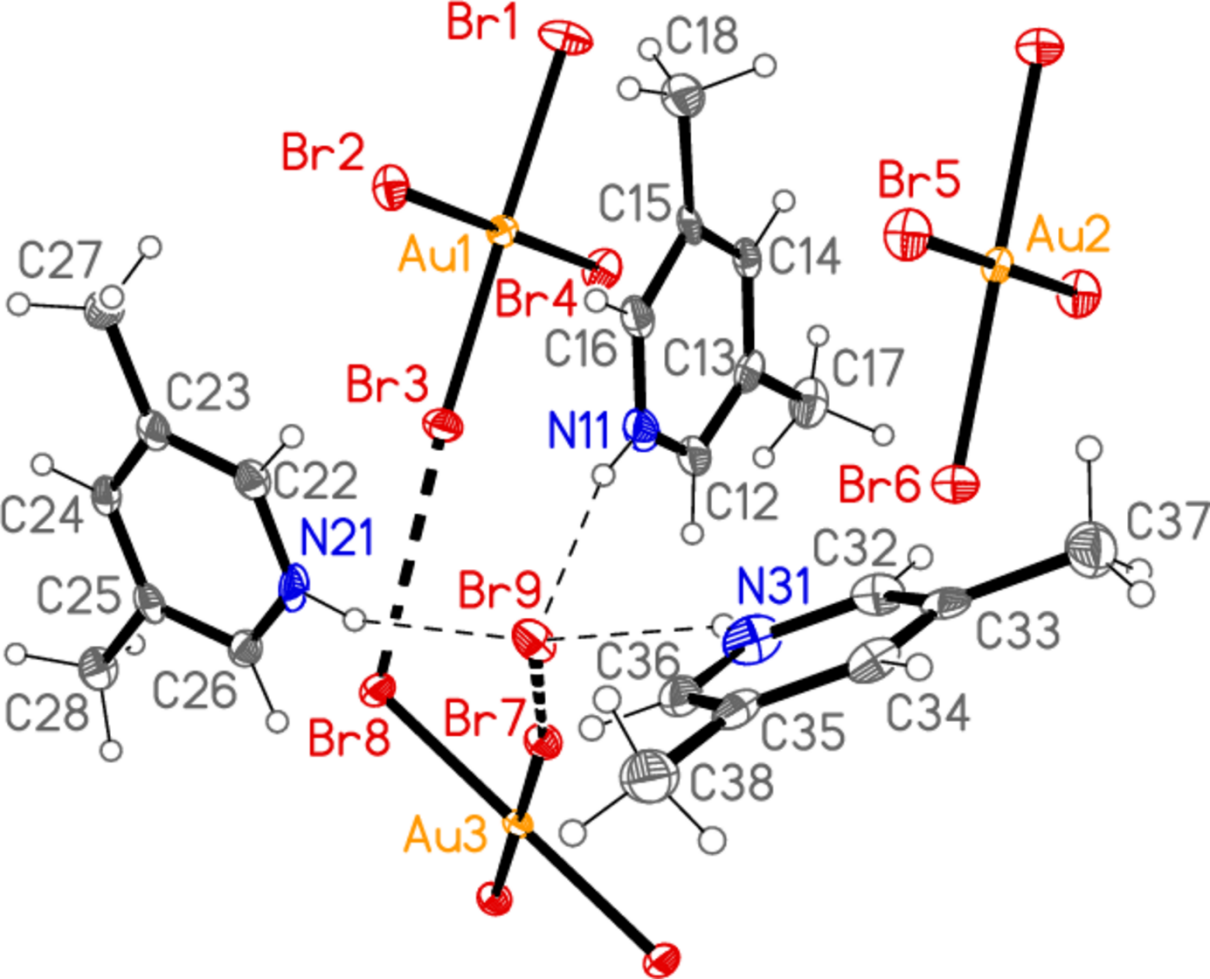
The formula unit of compound **4** in the crystal (extended by symmetry to show complete residues). Ellipsoids are drawn at the 50% probability level.

**Figure 7:**
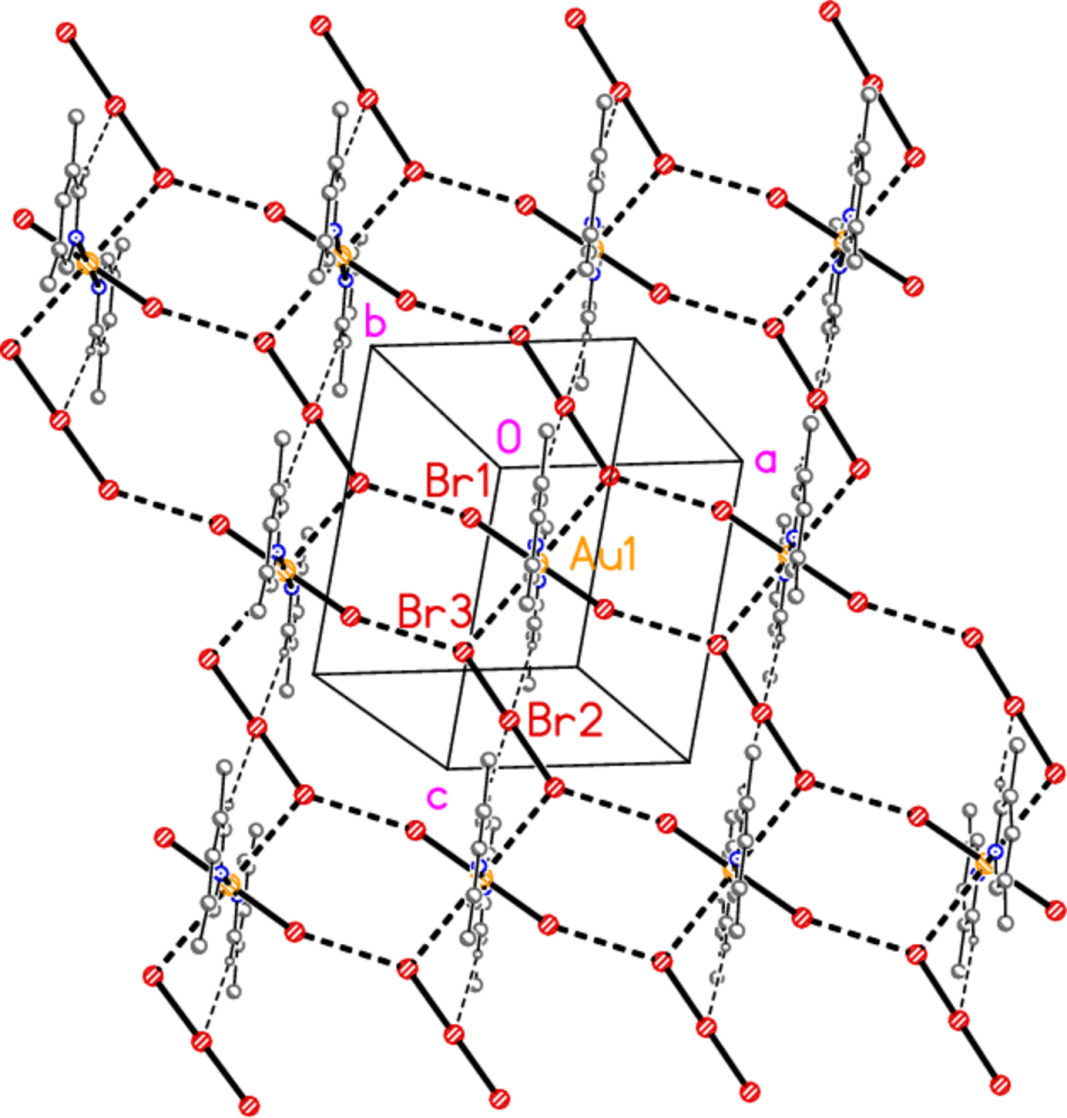
The packing of compound **1**, polymorph **1a**, viewed perpendicular to the *ac* plane. Dashed lines indicate Au⋯Br, Br⋯Br (thick) or H⋯Br (thin) contacts.

**Figure 8:**
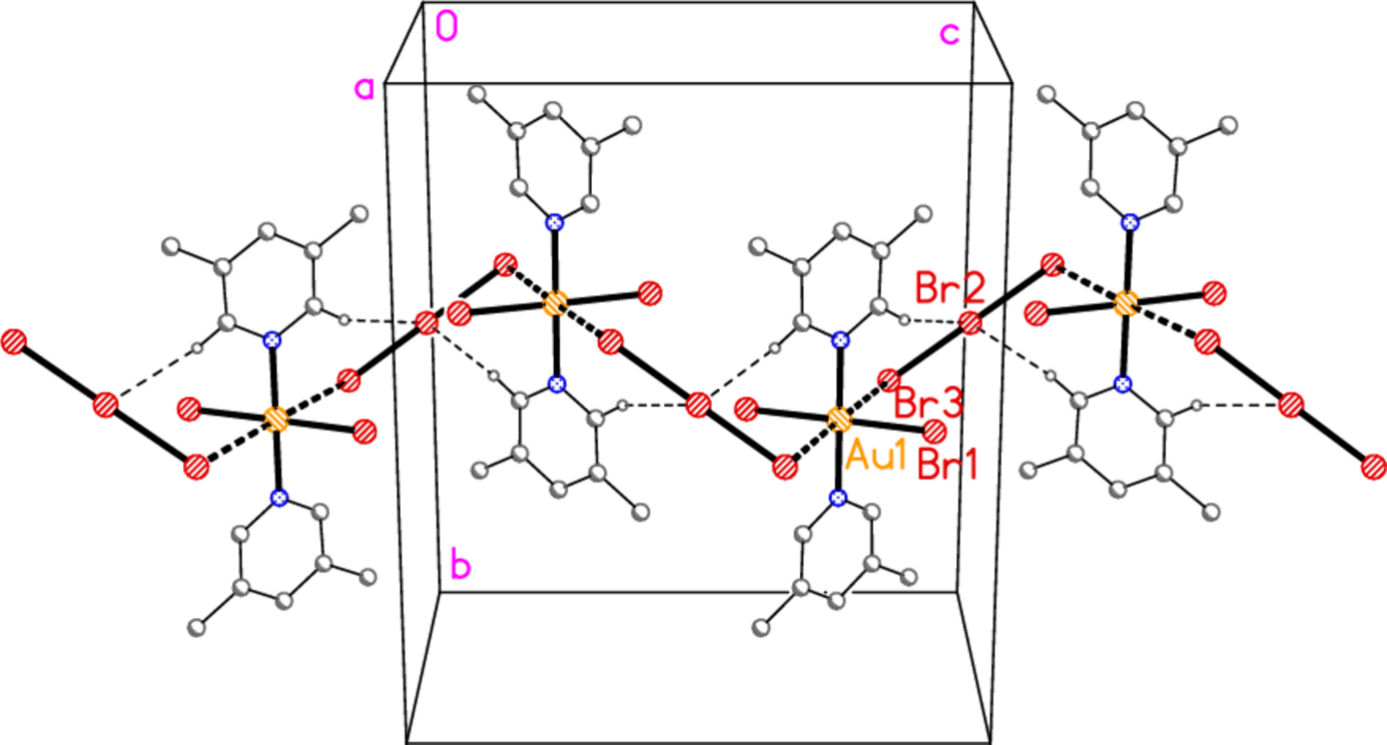
The packing of compound **1**, polymorph **1b**, viewed approximately parallel to the *a* axis (but slightly rotated horizontally). Dashed lines indicate Au⋯Br (thick) or H⋯Br (thin) contacts. Borderline Br⋯Br contacts (see text) are omitted.

**Figure 9:**
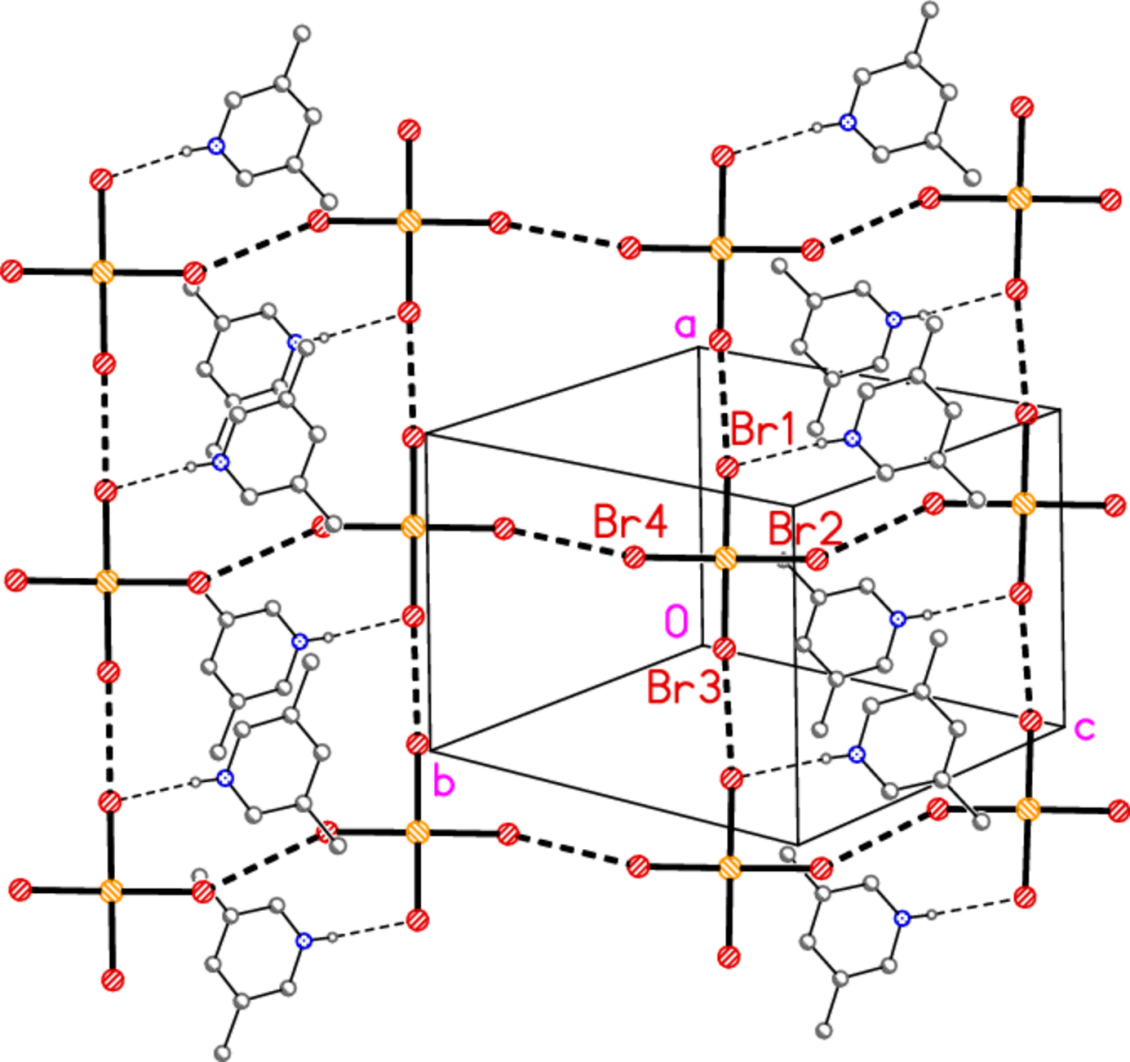
The packing of the previously known polymorph **2′** of compound **2** (Döring & Jones, 2016 ▸), viewed perpendicular to (011). Dashed lines indicate Br⋯Br contacts (thick) or hydrogen bonds (thin). The three independent Br⋯Br distances are Br1⋯Br3′ = 3.4751 (8), Br2⋯Br2′ = 3.6685 (13) and Br4⋯Br4′ = 3.6791 (12) Å.

**Figure 10:**
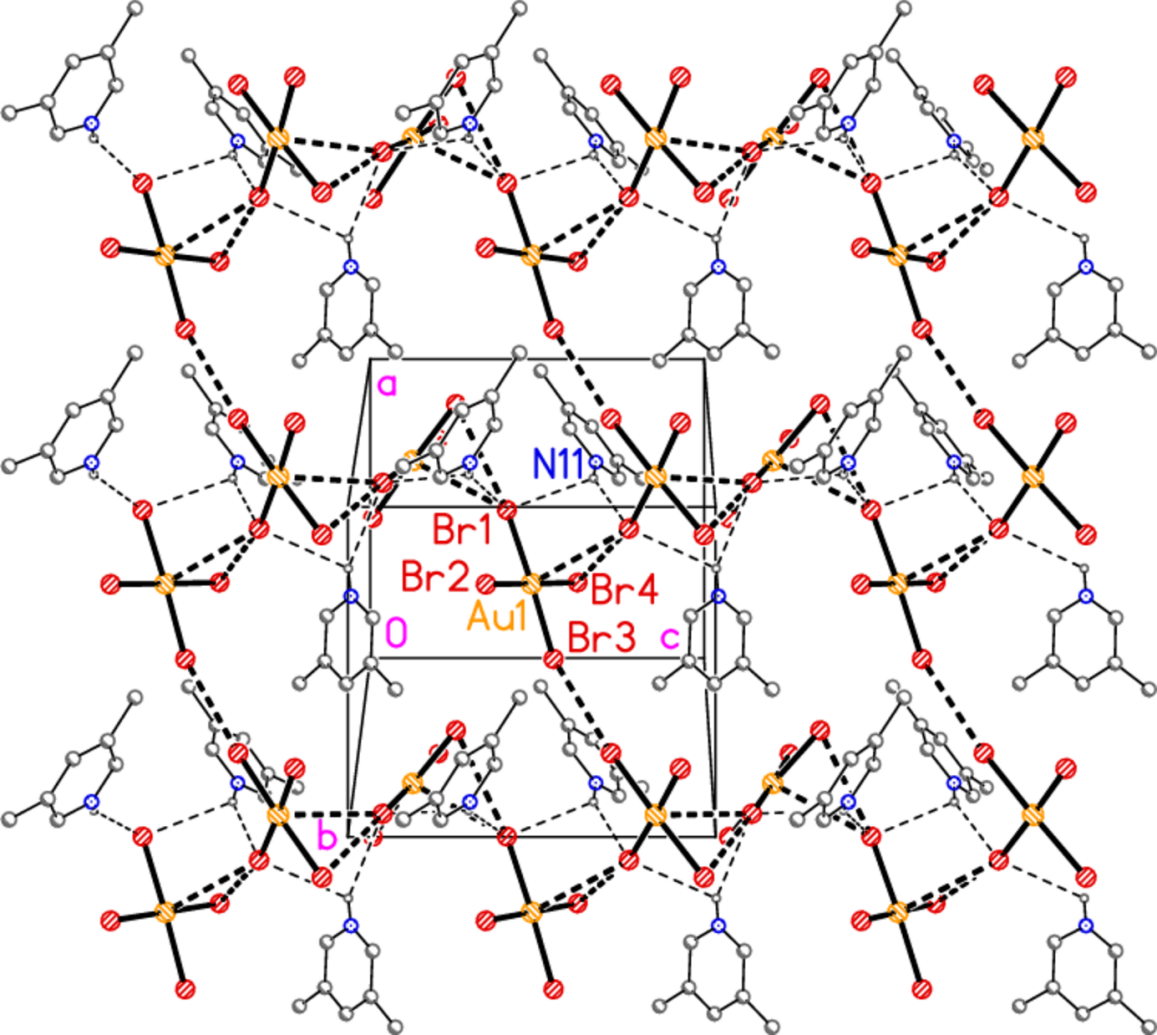
Packing diagram of compound **2** (new polymorph) viewed perpendicular to the *ac* plane in the region *y* ≃ 1. Dashed lines indicated Au⋯Br and Br⋯Br inter­actions (thick) or hydrogen bonds (thin).

**Figure 11:**
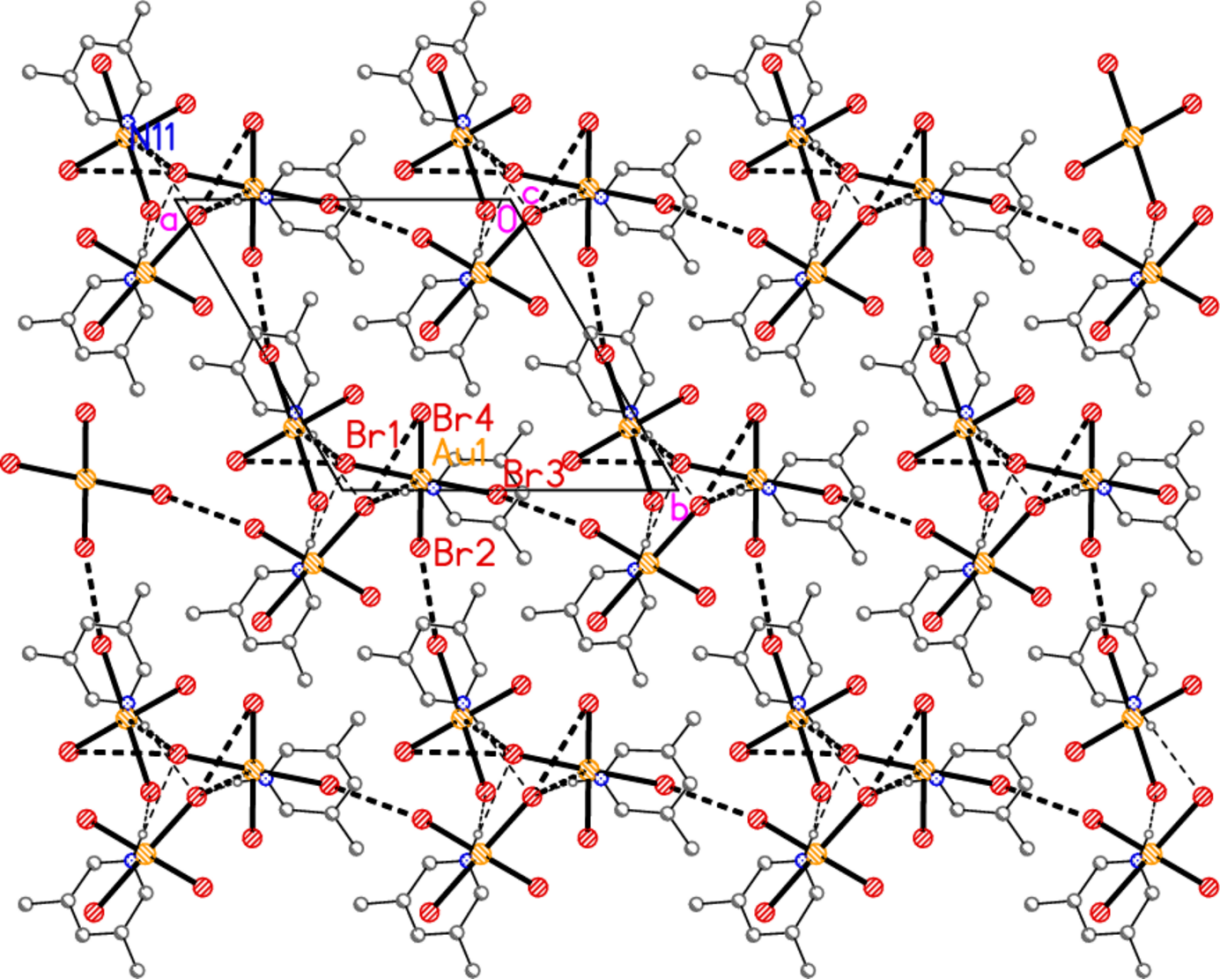
The packing of compound **2** (new polymorph) projected parallel to the *c* axis. Dashed lines indicate Au⋯Br and Br⋯Br inter­actions (thick) or hydrogen bonds (thin).

**Figure 12:**
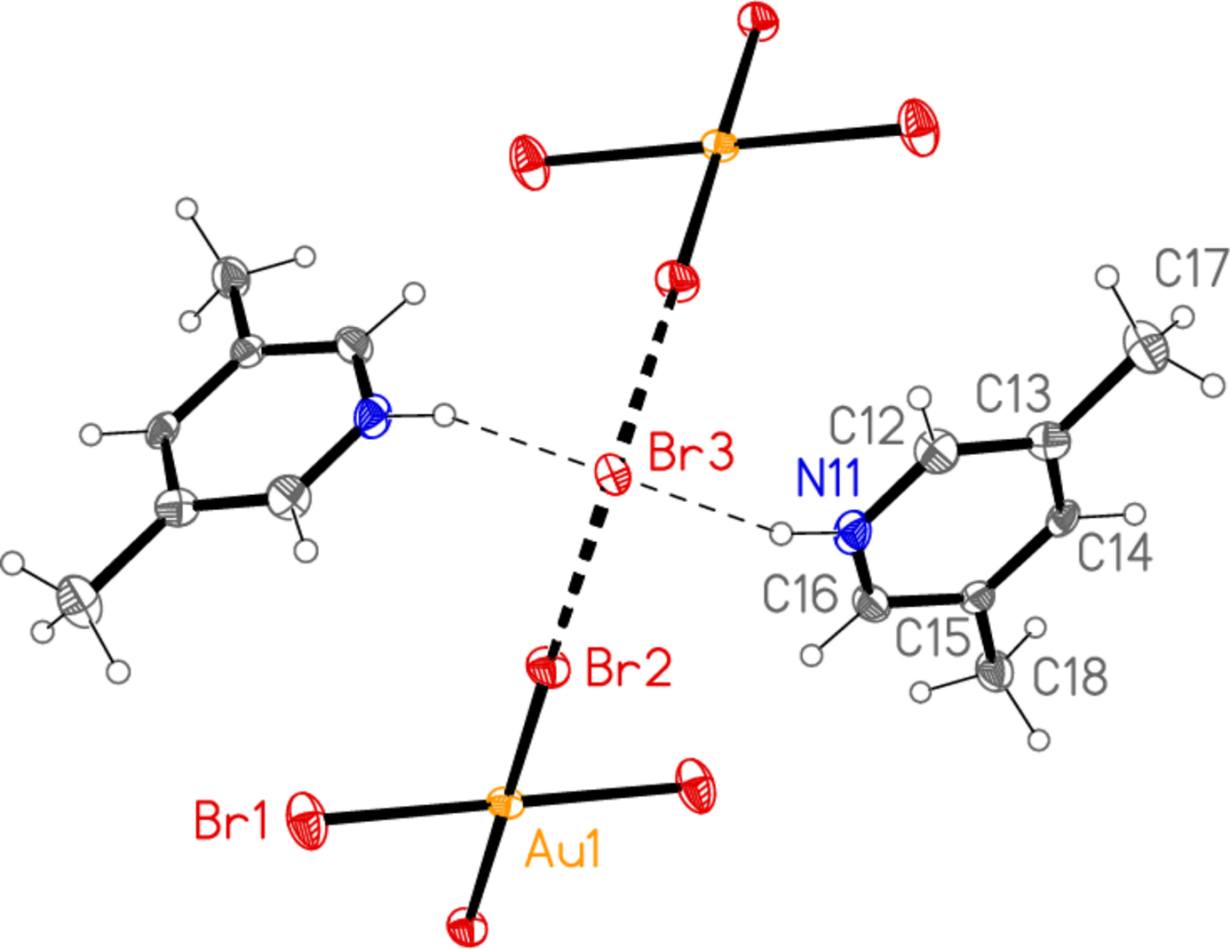
The dimeric unit of compound **3**, polymorph **a**, centred on the free bromide ion Br3. Dashed lines indicate Br⋯Br inter­actions (thick) or hydrogen bonds (thin).

**Figure 13:**
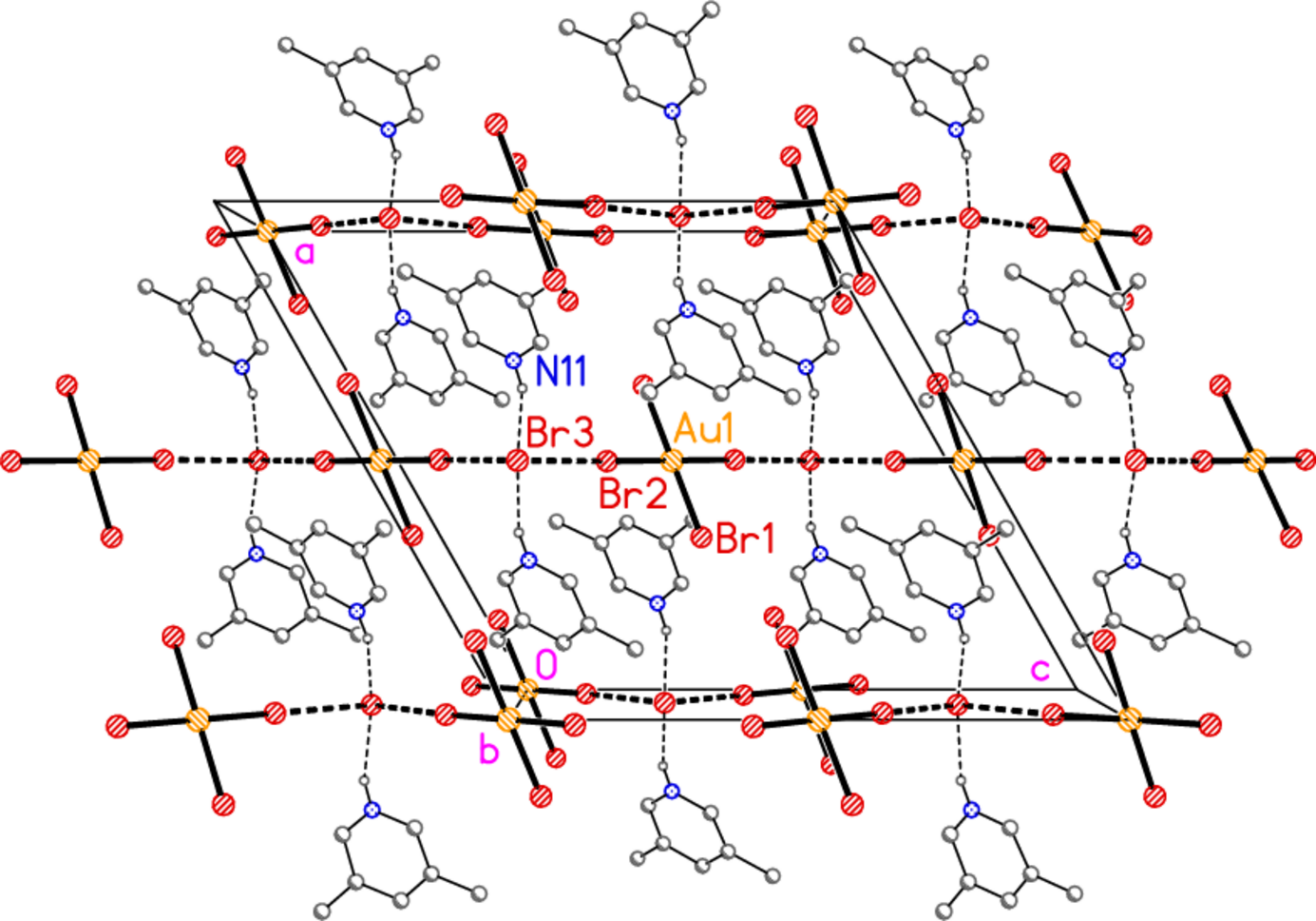
The extended packing of compound **3**, polymorph **a**, viewed parallel to the *b* axis. A chain of residues parallel to the *c* axis runs horizontally in the region *x* ≃ 0.5 (partial chains are shown in the regions *x* ≃ 0 and 1). Dashed lines indicate Br⋯Br inter­actions (thick) or hydrogen bonds (thin).

**Figure 14:**
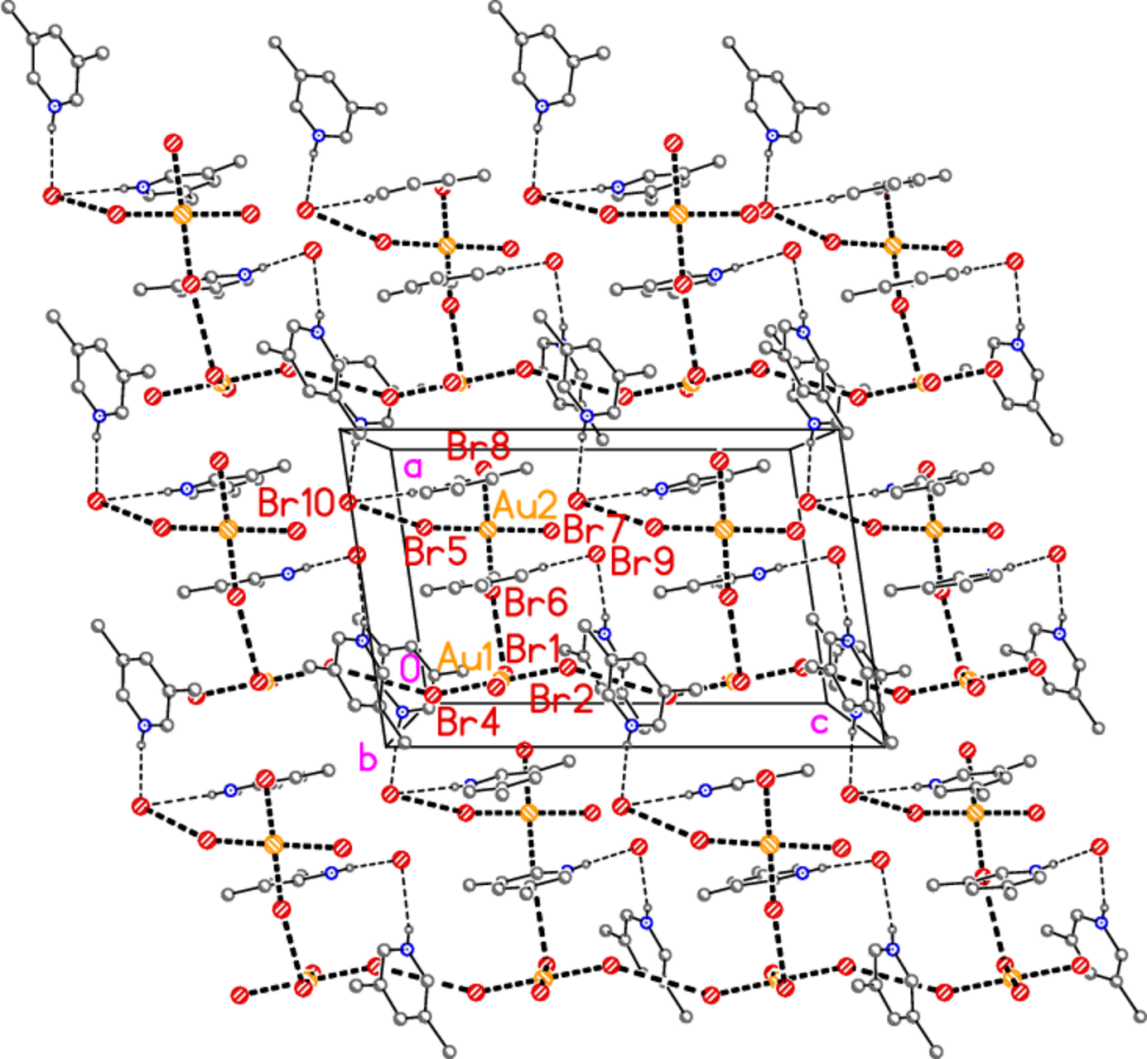
The packing of compound **3**, polymorph **b**, viewed parallel to the *b* axis in the region *y* ≃ 0.75. Three broad bands of residues parallel to the *c* axis can be recognized. Dashed lines indicate Br⋯Br inter­actions (thick) or hydrogen bonds (thin).

**Figure 15:**
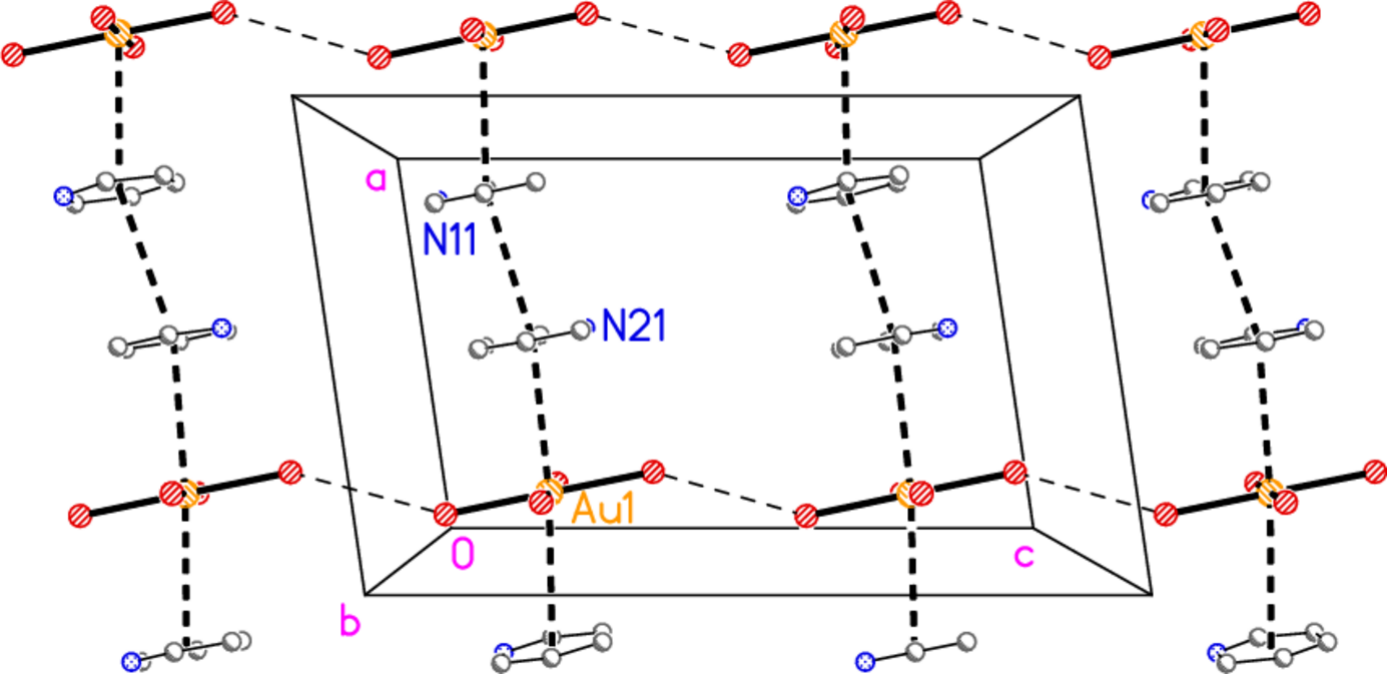
Stacking inter­actions in structure **3b** with view direction *etc*. as in Fig. 14 ▸. Dashed lines indicate stacking inter­actions (thick) or Br⋯Br contacts (thin).

**Figure 16:**
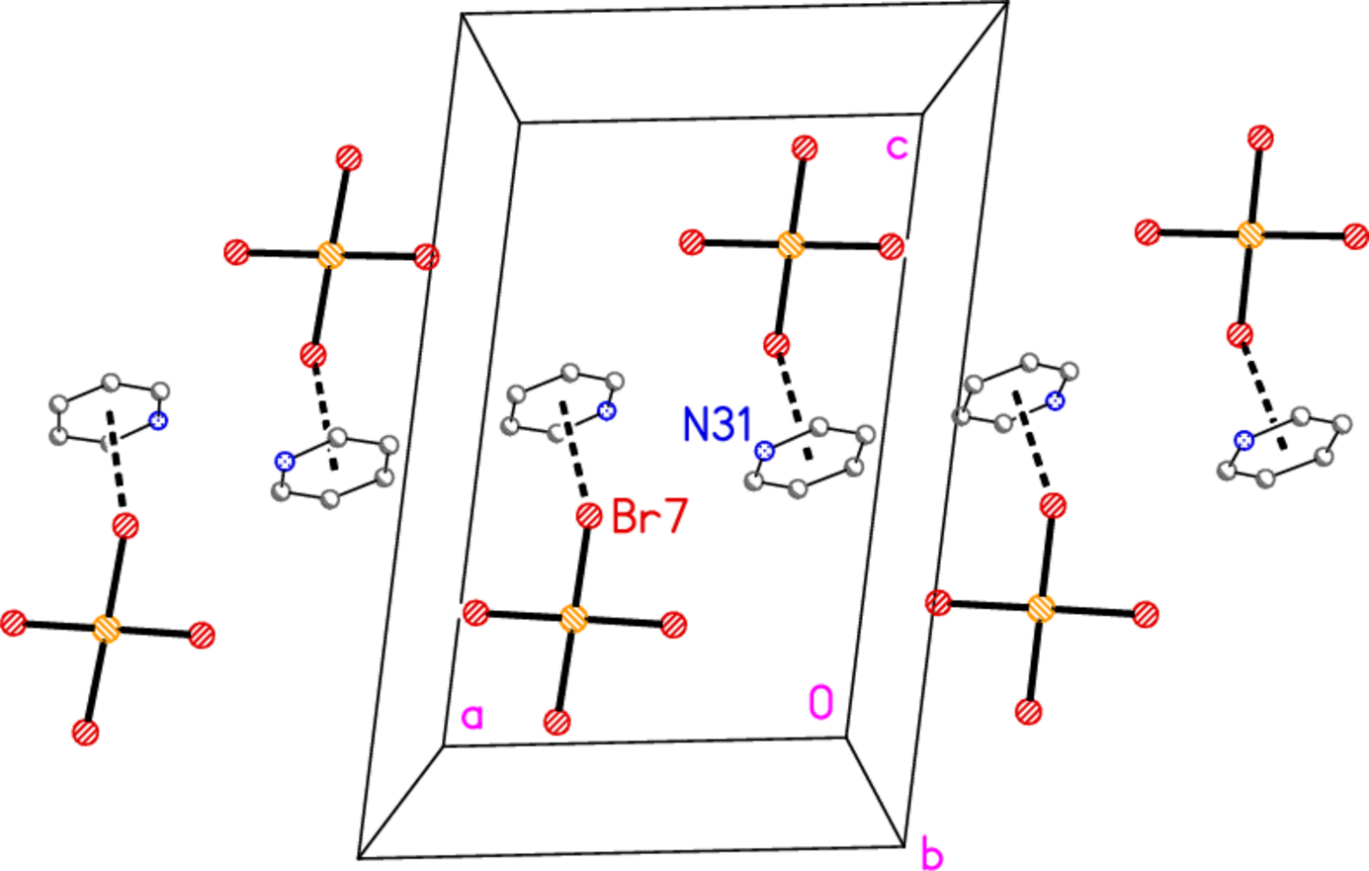
Br⋯π inter­actions in structure **3b** shown as thick dashed lines. The view direction is again parallel to the *b* axis, but in the region *y* ≃ 0.5.

**Figure 17:**
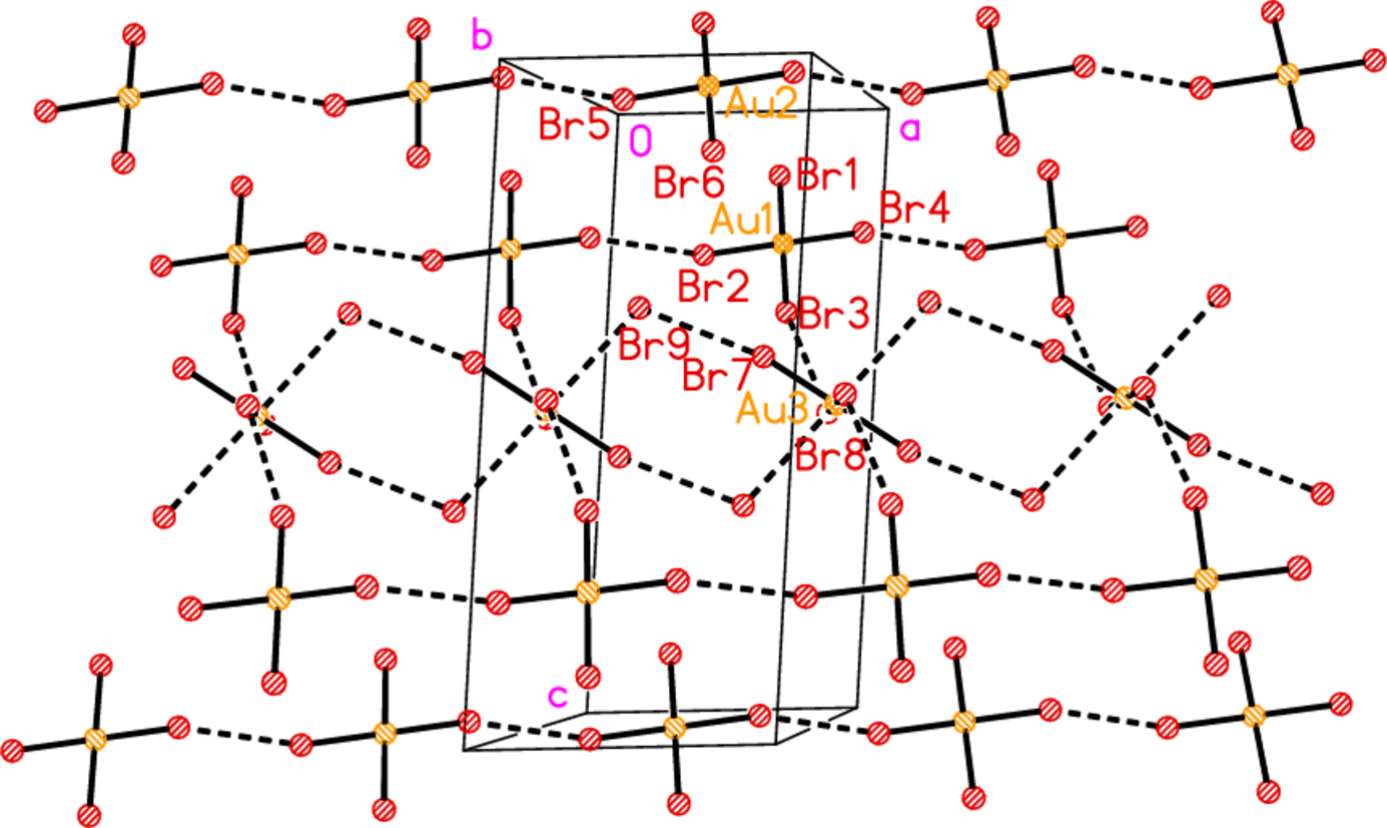
The packing of structure **4** showing only the anions, viewed perpendicular to the *ac* plane. Dashed lines indicate Br⋯Br and Au⋯Br contacts. The atom Br8 is almost eclipsed by Au3.

**Figure 18:**
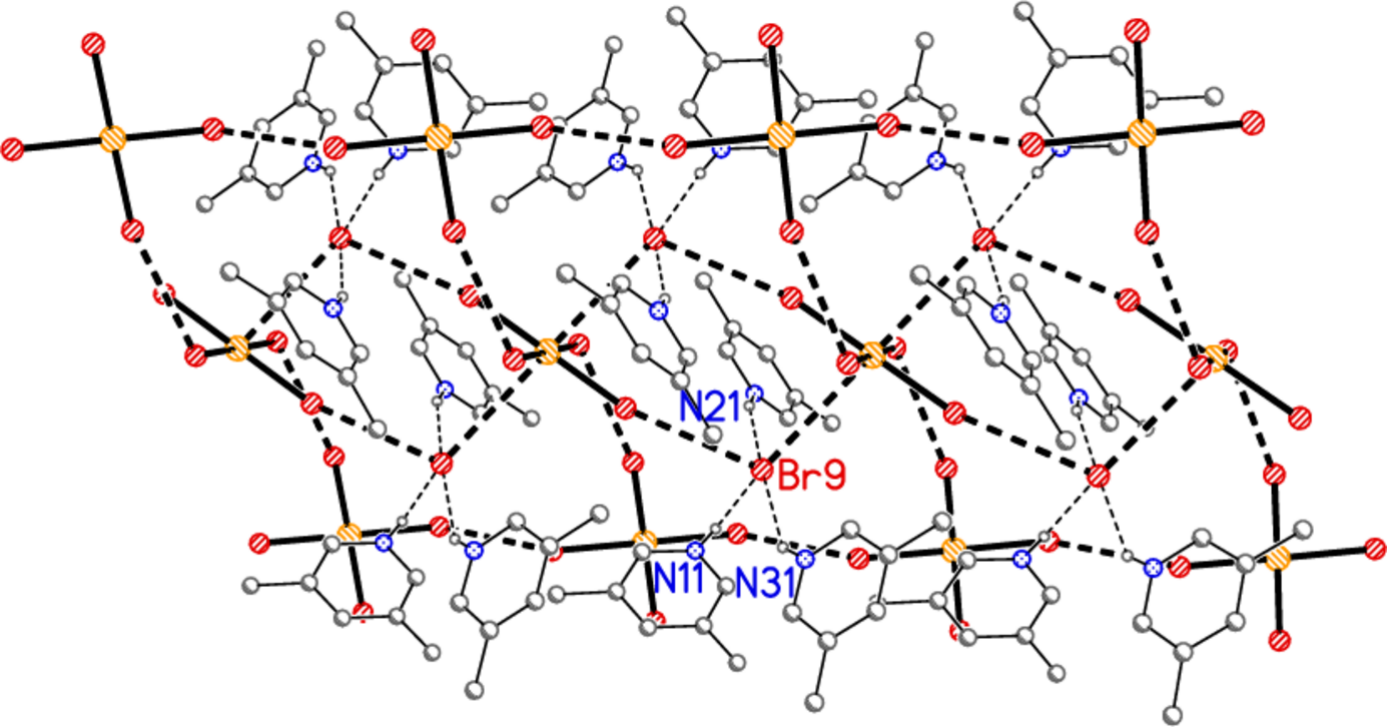
The packing of structure **4** including the cations but omitting the anions at Au2, with the same view direction as in Fig. 17 ▸. Dashed lines indicate Br⋯Br and Au⋯Br contacts (thick) or hydrogen bonds (thin).

The asymmetric unit of compound **4** (Fig. 6 ▸) was chosen to include as many short contacts as possible between the seven residues. All three cations are hydrogen bonded to the free bromide Br9, which is also involved in the contact Br7⋯Br9, 3.7404 (8) Å, with Au3—Br7⋯Br9 = 168.48 (2)°. The anions at Au1 and Au3 are linked *via* Br3⋯Br8, 3.4990 (8) Å, with Au1—Br3⋯Br8 = 162.26 (2) and Au3—Br8⋯Br3 = 110.41 (2)°. The anion at Au2 is not involved in short contacts within the asymmetric unit (see below for its role in the extended packing) but it does accept a weak hydrogen bond H32⋯Br6 (not drawn explicitly in Fig. 6 ▸). Similarly, the anions at Au1 and Au2 connect with ring 1, with Au1⋯*Cg*1 = 3.653 (2), Br6⋯*Cg*1 = 3.637 (2) Å and Au1⋯*Cg*1⋯Br6 = 164°; the anion at Au1 is almost parallel to ring 1 [inter­planar angle 5.2 (2)°]. These contacts too are not drawn explicitly for the sake of clarity. Fig. 17 ▸ shows the extended packing of the anions. The anions at Au2 form a chain parallel to the *a* axis (horizontal in the Figure, top and bottom) *via* the contact Br5⋯Br5(−*x*, 1 − *y*, −*z*) = 3.4414 (10) Å, with Au2—Br5⋯Br5′ = 157.80 (3)°. The anions at Au1 form a similar chain, also parallel to the *a* axis, with Br2⋯Br4(−1 + *x*, *y*, *z*) = 3.5253 (7) Å, Au1—Br2⋯Br4′ = 151.01 (2) and Au1—Br4⋯Br2′ = 152.39 (2)°. The anions at Au3 combine with the free bromide Br9 to form a chain of Au_2_Br_4_ rings parallel to the *a* axis, *via* the contacts Br7⋯Br9 and the axial contact Au3⋯Br9(1 + *x*, *y*, *z*) = 3.8611 (6) Å. These chains link with those at Au1 *via* the contacts Br3⋯Br8 to form a broad ribbon of residues. Fig. 18 ▸ shows the same view, but modified to show just the central ribbon, including cations and hydrogen bonds. The ribbons are joined by the Br⋯π contact Br2(*x*, 1 + *y*, *z*)⋯*Cg*3 = 3.769 (2) Å.

It is worth stressing that two less frequent types of secondary inter­action, namely halogen⋯π contacts (which may be considered as halogen bonds) and stacking of aromatic rings with planar anions, play a significant role in two of the structures described here. Yet these inter­actions can be difficult to find using standard programs and instructions. We used the ‘CENT/X’ command in *XP* (Bruker, 1998 ▸) to find the centres of gravity (labelled by the program as X1A, B, C, *etc*.) of the rings, and then used these pseudo-atoms to search for contacts. Even then, contacts within the asymmetric unit do not stand out because they have no symmetry operator, and the contacts to the centres of gravity may not be drawn because these are defined by XP as carbon (scattering factor type 1); to avoid this problem, we redefined the pseudo-atoms X as nitro­gen. Our personal view is that *XP* remains one of the best graphics programs despite its age.

## 4. Database survey

The previous publication in this series (Döring & Jones, 2025 ▸) presented a survey of structures involving both halide and tetra­halogenidoaurate(III) ions. For the current paper, a search for structures with stacking of [Au*X*_4_]^−^ anions and six-membered aromatic rings was performed. Ring atoms were restricted to C or N. The inter­planar angle was restricted to the range 0–5°, and the maximum distance between the gold atom and the centroid of the ring was originally set to 3.8 Å. This gave 20 hits. To restrict the hits to the shortest distances, the maximum distance was then reduced to 3.6 Å, whereby five hits remained. These were: 2-(pyrimidin-2-yl)pyrimidin-1-ium tetra­chlorido­aurate(III) (refcode AHIYEX; Chernyshev *et al.*, 2015 ▸); *N*-{[4-(acetamido­meth­yl)-2,3,5,6-tetra­methyl­phen­yl]meth­yl}-1- hy­droxy­ethan-1-iminium tetra­chlorido­aurate(III) (FACGID; Shaffer *et al.*, 2021 ▸); tri­phenyl­telluronium tetra­chlorido­aurate(III) (MIHSOL; Oilunkaniemi *et al.*, 2001 ▸); di­chloro­(4,4′-dimethyl-2,2′-bi­pyridine)­gold tetra­chlorido­aurate(III) (NOKREM; Amani *et al.*, 2014 ▸); and 6,6′′-dimethyl-2,2′:6′,2′′-terpyridin-1,1′′-di-ium bis­[tetra­chlorido­aurate(III)] monohydrate (TIRMAL; Bocian *et al.*, 2019 ▸). In three of these publications, the stacking was not discussed (the focus of the publications lay elsewhere, and such inter­actions are not as easily recognized as, say, hydrogen bonds), but Shaffer *et al.* (2021 ▸) gave an extensive description of the stacking of FACGID (infinite stacks of alternating [AuCl_4_]^−^ anions and durene rings) and its potential for extracting tetra­halogenidoaurates(III) using durene derivatives, and Amani *et al.* (2014 ▸) presented the stacking of NOKREM (infinite stacks of alternating [AuCl_4_]^−^ anions and pyridine rings) in some detail. Tiekink & Zukerman-Schpector (2009 ▸) have published a review of stacking involving gold complexes, but this was restricted to C_6_ rings.

**Figure 19:**
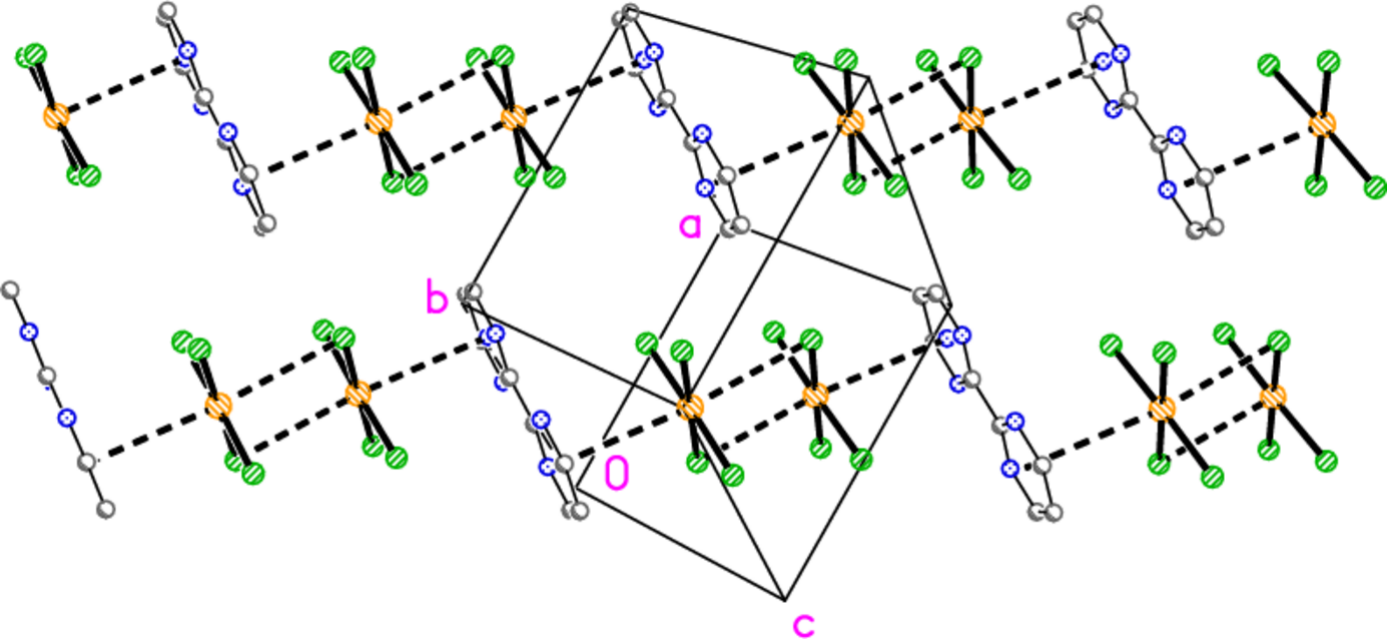
The packing of AHIYEX (Chernyshev *et al.*, 2015 ▸) drawn using the deposited coordinates, viewed perpendicular to (011). Dashed lines indicate Au⋯*Cg* or Au⋯Cl contacts. The space group is *P*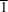 and all atoms lie on general positions.

**Figure 20:**
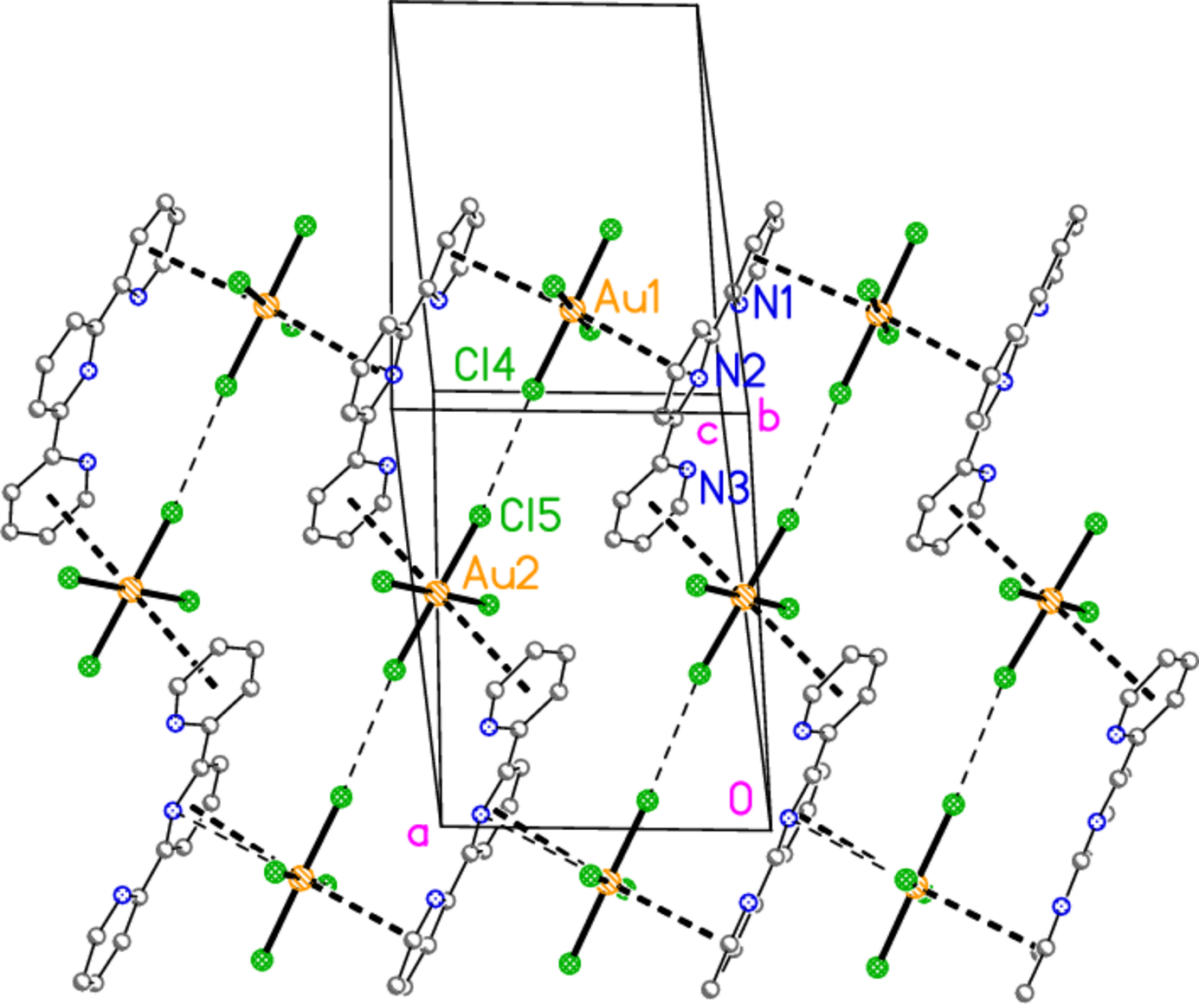
The packing of TIRMAL (Bocian *et al.*, 2019 ▸) drawn using the deposited coordinates, viewed perpendicular to (01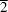). Dashed lines indicate Au⋯*Cg* (thick) or Cl⋯Cl (thin) contacts. The ribbons run parallel to the *a* axis. The *z* coordinates were reduced by 0.5 to give a better fit to the cell. The space group is *P*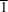; atoms Au2 lie on inversion centres.

The packing of AHIYEX (Fig. 19 ▸) involves a substructure in which pairs of offset-stacked [AuCl_4_]^−^ anions with Au⋯Cl = 3.455 Å are flanked by bipyrimidinium cations (Au⋯*Cg* = 3.567 Å) to give stacks of four planes; offset stacking is a well-known feature of tri- or tetra­halogenidogold(III) centres, *e.g.* in (tht)AuCl_3_ (Upmann *et al.*, 2017 ▸). The stacks propagate in the direction [1
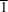
1]. The packing of MIHSOL was, justifiably, analysed in terms of Te⋯Cl contacts, but the structure also contains isolated *Cg*(phen­yl)⋯Au⋯*Cg*(phen­yl) stacks with distances of 3.525 and 3.736 Å and an angle of 175.9°. For TIRMAL, a ribbon substructure (Fig. 20 ▸) can be recognized that involves three Au⋯*Cg* inter­actions [Au1⋯*Cg*1 = 3.569, Au1⋯*Cg*3 = 3.487 and Au2⋯*Cg*2 = 3.645 Å (× 2)], so that each ring of the terpyridine system is involved. Cl⋯Cl contacts of 3.467 Å also contribute to the ribbon.

The searches employed the routine ConQuest (Bruno *et al.*, 2002 ▸), part of Version 2024.3.0 of the Cambridge Database (Groom *et al.*, 2016 ▸).

## 5. Synthesis and crystallization

*Compounds **2**, **3** (polymorph **b**), **4***: 90 mg (0.247 mmol) of (tht)AuBr were added to 2 mL of 3,5-lutidine. The mixture was sonicated and the white solid allowed to settle. The supernatant solution was pipetted off and the solid, presumed to be [(3,5-Lut)_2_Au][AuBr_2_] (Döring & Jones, 2024*b* ▸), was dissolved in 2 mL di­chloro­methane. The clear colourless solution was distributed over five ignition tubes and overlayered with various precipitants, before being stored in a refrigerator overnight. In the tube with diisopropyl ether as precipitant, well-formed red hexa­gonal blocks of **4** together with some red plates of **3b** were found. We were unable to establish how the oxidation had taken place, because no bromine was added. Two possibilities would be aerial oxidation or disproportionation. For compound **2**, the same method was used, but two drops of bromine were added to the di­chloro­methane solution before overlayering. In the tube with *n*-heptane as precipitant, red needles and prisms of **2** formed.

*Compound **3** (polymorph **a**)*: [(3,5-Lut)_2_Au][AuBr_2_] was obtained as above, but from the supernatant solution, which was transferred to a round-bottomed flask and overlayered with petroleum ether until a permanent turbidity was observed. The solid product (26.8 mg) was dried under vacuum and dissolved in 2 mL of di­chloro­methane. After the addition of two drops of bromine, the solution was overlayered as above. In the tube using diisopropyl ether as precipitant, red blocks and plates of **3a** formed.

*Compounds **1a***, ***1b***: [(3,5-Lut)_2_Au][AuBr_2_] was obtained as above, but on a larger scale; 151 mg (0.414 mmol) were dissolved by sonication in 8 mL di­chloro­methane. The flask was connected *via* an angled tube to a further flask, containing 10 mL of di­chloro­methane and excess bromine, to allow slow diffusion. After one month, the solution had become red, and large red crystals had formed on the walls of the flask. This was then disconnected from the bromine solution and allowed to stand for a further month, by which time the solvent had evaporated. A red block was investigated and led to structure **1b**. There were also a few smaller red blocks of a slightly different appearance, which proved to be the other polymorph **1a**. A satisfactory analysis was obtained: Calculated C 20.74, H 2.24, N 3.45; found C 20.78, H 2.21, N 3.57%.

More details are given in the PhD thesis of CD (Döring, 2016 ▸).

## 6. Refinement

Details of the measurements and refinements are given in Table 13 ▸. Structures were refined anisotropically on *F*^2^. Data for **3b** are weak (with a correspondingly high value of *R*_int_) but establish the existence of the second polymorph of **3**. Hydrogen atoms of the NH groups were refined freely. For compounds **3b** and **4**, N—H distances were restrained to be approximately equal (command ‘SADI’) and a common isotropic *U* value was employed for the NH hydrogen atoms. Hydrogen atoms of the lutidine rings were included at calculated positions and refined using a riding model with C—H = 0.95 Å. Methyl groups were included as idealized rigid groups with C—H = 0.98 Å and H—C—H = 109.5°, and were allowed to rotate but not tip (command ‘AFIX 137’), but the convergence was in many cases slow, and the methyl hydrogen positions should be inter­preted with caution (see below). *U* values of the hydrogen atoms were fixed at 1.5 × *U*_eq_ of the parent carbon atoms for methyl groups and 1.2 × *U*_eq_ of the parent carbon atoms for other hydrogens. For structures **1b** and **3b**, the second weighting parameter *b* oscillated over a small range. Structures **1b** and **2** crystallize only by chance in Sohncke space groups; the compounds are achiral.

*checkCIF* alerts: For compound **3a**, the cell as originally determined (at a time when *C*-centred monoclinic settings were preferred to *I*-centred) can be transformed by the matrix (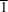 0 0 / 0 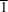 0 / 1 0 1) to an *I*-centred cell with *a* = 15.567, *b* = 9.578, *c* = 16.007 Å and β = 118.11°, space group *I*2/*a*. The β angle is slightly smaller than that of the original cell, which is thus formally non-reduced, causing checkCIF alert G ‘PLAT158’. The positions of the methyl hydrogens at C17 were assigned using ‘AFIX 137’, which detected three clear maxima in the residual electron density. Nevertheless, the refinement converged slowly and *checkCIF* found negative electron density at the hydrogen positions (alert G ‘PLAT977’. It is possible that this methyl group is rotationally disordered. For compound **3b**, the asymmetric unit was chosen to maximize the number of contacts contained therein. This leads to a position for the lutidine at N41 with most *x* coordinates > 1 and thus a centre of gravity outside the unit cell, which causes a *checkCIF* alert G ‘PLAT790’. Despite this alert, we prefer the chosen position.

## Supplementary Material

Crystal structure: contains datablock(s) 1a, 1b, 2, 3a, 3b, 4, global. DOI: 10.1107/S2056989025006401/yz2069sup1.cif

Structure factors: contains datablock(s) 1a. DOI: 10.1107/S2056989025006401/yz20691asup8.hkl

Structure factors: contains datablock(s) 1b. DOI: 10.1107/S2056989025006401/yz20691bsup3.hkl

Structure factors: contains datablock(s) 2. DOI: 10.1107/S2056989025006401/yz20692sup4.hkl

Structure factors: contains datablock(s) 3a. DOI: 10.1107/S2056989025006401/yz20693asup5.hkl

Structure factors: contains datablock(s) 3b. DOI: 10.1107/S2056989025006401/yz20693bsup6.hkl

Structure factors: contains datablock(s) 4. DOI: 10.1107/S2056989025006401/yz20694sup7.hkl

CCDC references: 2473668, 2473667, 2473666, 2145227, 2145228, 2145229

Additional supporting information:  crystallographic information; 3D view; checkCIF report

## Figures and Tables

**Figure 2:**
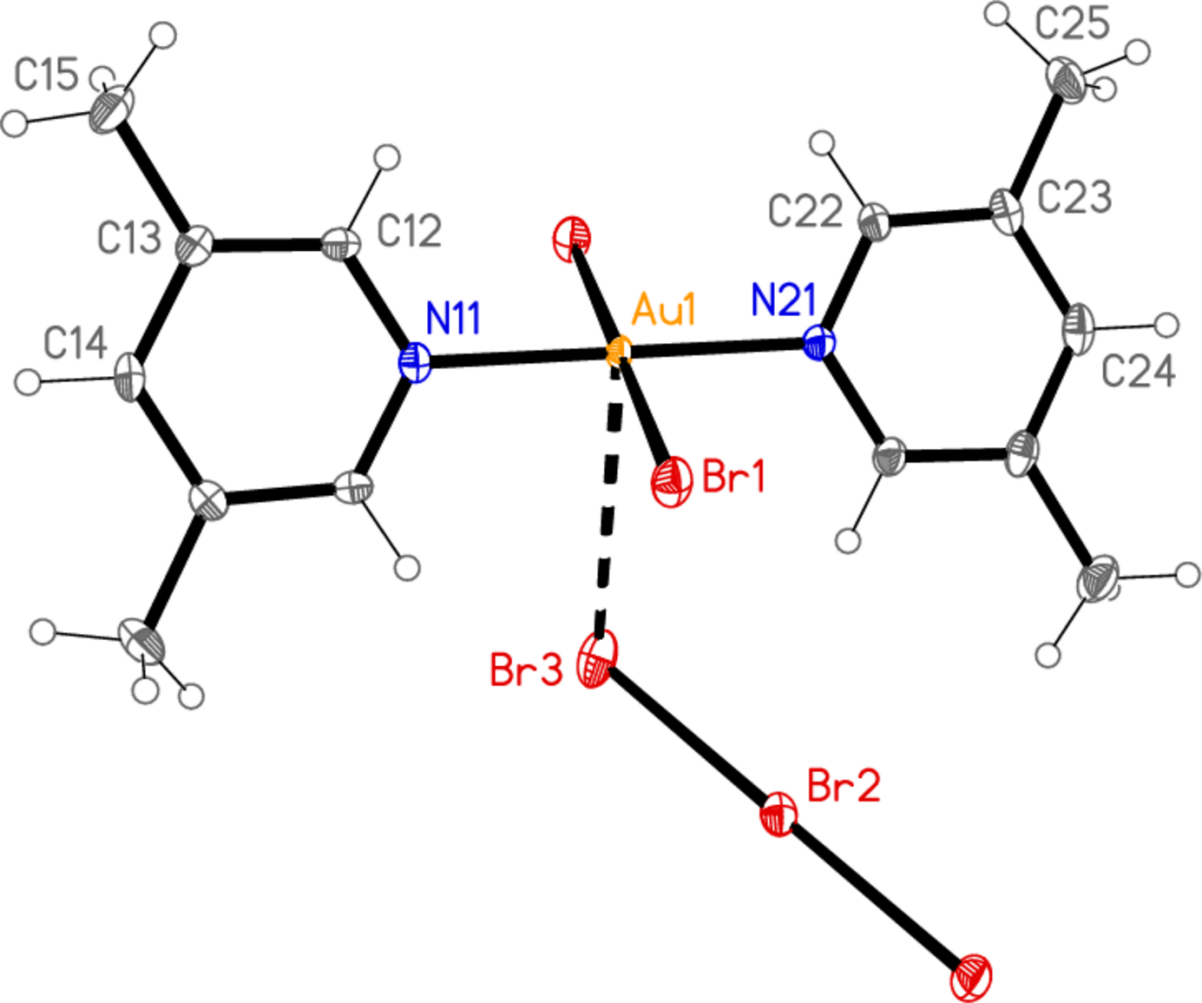
The formula unit of compound **1**, polymorph **1b**, in the crystal. Ellipsoids are drawn at the 50% probability level.

**Figure 3:**
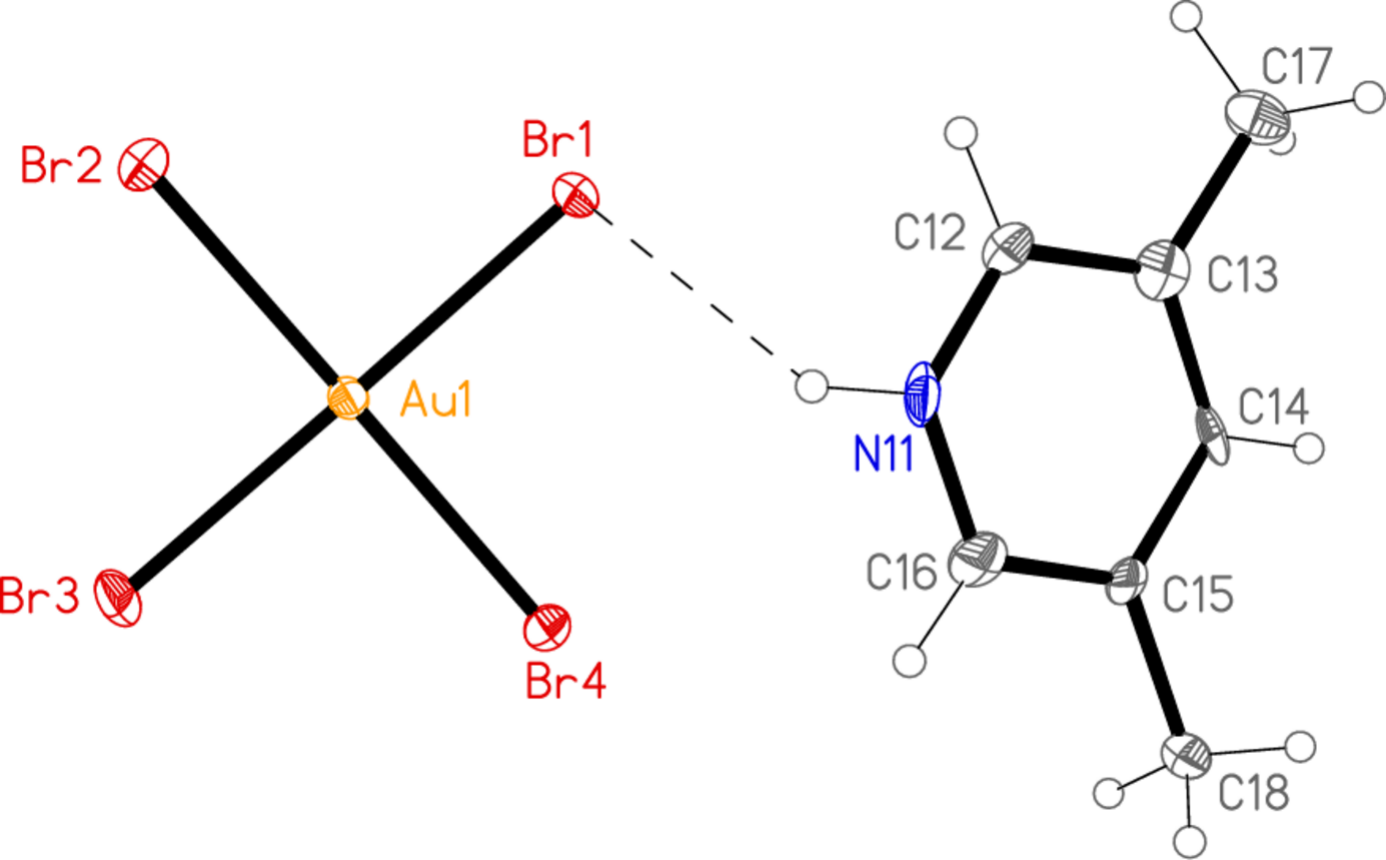
The formula unit of compound **2** in the crystal. Ellipsoids are drawn at the 50% probability level.

**Figure 4:**
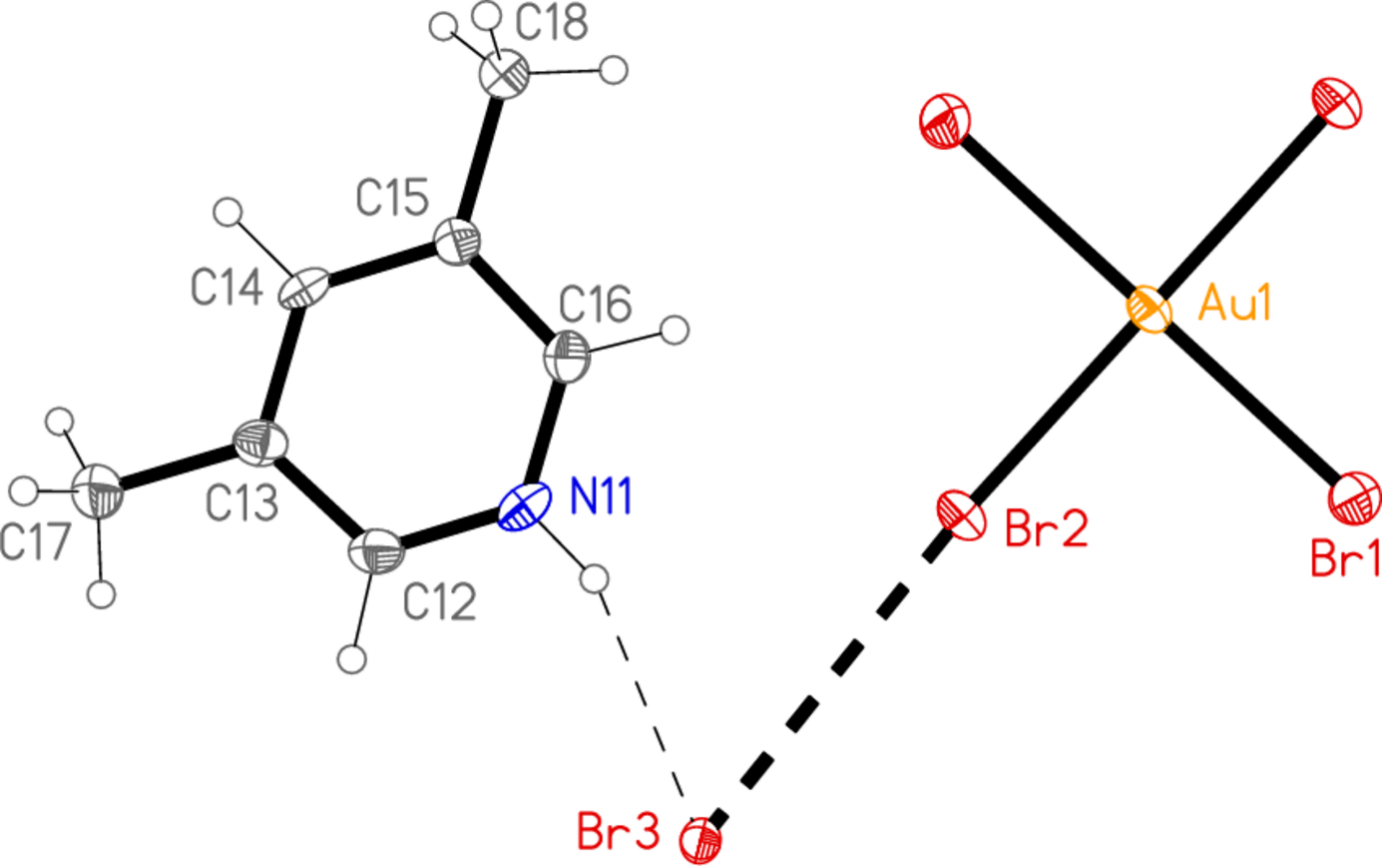
The formula unit of compound **3**, polymorph **3a**, in the crystal. Ellipsoids are drawn at the 50% probability level.

**Table 1:** Selected geometric parameters (Å, °) for **1a**[Chem scheme1]

Au1—N11	2.025 (2)	N11—C12	1.340 (3)
Au1—Br1	2.4174 (3)	N11—C16	1.343 (3)
Br2—Br3	2.5385 (3)		
			
N11^i^—Au1—N11	180.0	Br1^i^—Au1—Br1	180.0
N11—Au1—Br1^i^	89.99 (6)	Br3^ii^—Br2—Br3	180.0
N11—Au1—Br1	90.01 (6)	C12—N11—C16	120.6 (2)

Symmetry codes: (i) 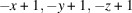; (ii) 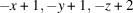.

**Table 2:** Selected geometric parameters (Å, °) for **1b**[Chem scheme1]

Au1—N11	2.020 (4)	Br2—Br3	2.5388 (5)
Au1—N21	2.032 (4)	N11—C12	1.347 (5)
Au1—Br1	2.4090 (4)	N21—C22	1.346 (5)
			
N11—Au1—N21	180.0	Br3^ii^—Br2—Br3	179.55 (3)
N11—Au1—Br1	89.753 (12)	C12^i^—N11—C12	120.5 (5)
N21—Au1—Br1	90.247 (12)	C22^i^—N21—C22	121.5 (5)
Br1^i^—Au1—Br1	179.51 (2)		

Symmetry codes: (i) 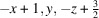; (ii) 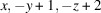.

**Table 3:** Selected geometric parameters (Å, °) for **2**[Chem scheme1]

Au1—Br3	2.4186 (10)	Au1—Br1	2.4285 (10)
Au1—Br2	2.4254 (10)	N11—C12	1.338 (11)
Au1—Br4	2.4285 (9)	N11—C16	1.340 (12)
			
Br3—Au1—Br2	90.31 (4)	Br2—Au1—Br1	89.01 (3)
Br3—Au1—Br4	89.45 (3)	Br4—Au1—Br1	91.32 (3)
Br2—Au1—Br4	177.81 (4)	C12—N11—C16	124.0 (8)
Br3—Au1—Br1	177.69 (4)		

**Table 4:** Selected geometric parameters (Å, °) for **3a**[Chem scheme1]

Au1—Br1	2.4197 (4)	N11—C16	1.332 (5)
Au1—Br2	2.4280 (4)	N11—C12	1.334 (5)
			
Br1^i^—Au1—Br1	180.0	Br2—Au1—Br2^i^	180.0
Br1—Au1—Br2	89.450 (16)	C16—N11—C12	123.0 (4)
Br1—Au1—Br2^i^	90.550 (16)		

Symmetry code: (i) 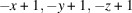.

**Table 5:** Selected geometric parameters (Å, °) for **3b**[Chem scheme1]

Au1—Br3	2.4222 (8)	N11—C12	1.310 (11)
Au1—Br1	2.4268 (8)	N11—C16	1.337 (11)
Au1—Br2	2.4269 (7)	N21—C22	1.339 (10)
Au1—Br4	2.4276 (7)	N21—C26	1.356 (9)
Au2—Br6	2.4060 (8)	N31—C36	1.331 (9)
Au2—Br8	2.4122 (9)	N31—C32	1.342 (9)
Au2—Br7	2.4142 (8)	N41—C42	1.329 (10)
Au2—Br5	2.4232 (8)	N41—C46	1.340 (10)
			
Br3—Au1—Br1	179.57 (3)	Br8—Au2—Br7	90.49 (3)
Br3—Au1—Br2	89.34 (3)	Br6—Au2—Br5	89.54 (3)
Br1—Au1—Br2	90.39 (3)	Br8—Au2—Br5	90.35 (3)
Br3—Au1—Br4	90.04 (3)	Br7—Au2—Br5	176.77 (3)
Br1—Au1—Br4	90.23 (3)	C12—N11—C16	124.3 (7)
Br2—Au1—Br4	179.30 (3)	C22—N21—C26	123.5 (7)
Br6—Au2—Br8	179.17 (4)	C36—N31—C32	123.0 (6)
Br6—Au2—Br7	89.66 (3)	C42—N41—C46	123.6 (7)

**Table 6:** Selected geometric parameters (Å, °) for **4**[Chem scheme1]

Au1—Br2	2.4203 (5)	Au3—Br7	2.4300 (5)
Au1—Br3	2.4206 (6)	N11—C12	1.321 (7)
Au1—Br1	2.4255 (6)	N11—C16	1.333 (7)
Au1—Br4	2.4285 (5)	N21—C26	1.333 (7)
Au2—Br6	2.4159 (6)	N21—C22	1.341 (7)
Au2—Br5	2.4210 (5)	N31—C36	1.334 (7)
Au3—Br8	2.4174 (5)	N31—C32	1.345 (7)
			
Br2—Au1—Br3	90.39 (2)	Br5—Au2—Br5^i^	180.0
Br2—Au1—Br1	90.16 (2)	Br8—Au3—Br8^ii^	180.0
Br3—Au1—Br1	179.13 (2)	Br8—Au3—Br7^ii^	88.897 (18)
Br2—Au1—Br4	178.57 (2)	Br8—Au3—Br7	91.104 (18)
Br3—Au1—Br4	89.93 (2)	Br7^ii^—Au3—Br7	180.0
Br1—Au1—Br4	89.55 (2)	C12—N11—C16	123.1 (5)
Br6^i^—Au2—Br6	180.00 (3)	C26—N21—C22	123.3 (5)
Br6—Au2—Br5	89.722 (19)	C36—N31—C32	123.8 (5)
Br6—Au2—Br5^i^	90.279 (19)		

Symmetry codes: (i) 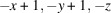; (ii) 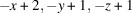.

**Table 7:** Hydrogen-bond geometry (Å, °) for **1a**[Chem scheme1]

*D*—H⋯*A*	*D*—H	H⋯*A*	*D*⋯*A*	*D*—H⋯*A*
C14—H14⋯Br1^iii^	0.95	2.95	3.762 (3)	144
C12—H12⋯Br2	0.95	2.76	3.683 (3)	165
C12—H12⋯Br3	0.95	3.05	3.718 (3)	129
C16—H16⋯Br3^i^	0.95	3.00	3.689 (3)	130
C17—H17*C*⋯Br3^iii^	0.98	3.04	3.946 (3)	155

Symmetry codes: (i) 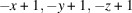; (iii) 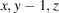.

**Table 8:** Hydrogen-bond geometry (Å, °) for **1b**[Chem scheme1]

*D*—H⋯*A*	*D*—H	H⋯*A*	*D*⋯*A*	*D*—H⋯*A*
C15—H15*A*⋯Br1^iii^	0.98	3.01	3.500 (5)	112
C22—H22⋯Br1^i^	0.95	3.05	3.367 (4)	102
C22—H22⋯Br2^iv^	0.95	2.88	3.780 (4)	159
C12—H12⋯Br3^i^	0.95	2.90	3.662 (4)	138
C14—H14⋯Br3^v^	0.95	3.04	3.802 (4)	138
C14—H14⋯Br3^vi^	0.95	3.04	3.802 (4)	138
C24—H24⋯Br3^vii^	0.95	2.96	3.708 (4)	137
C24—H24⋯Br3^viii^	0.95	2.96	3.708 (4)	137

Symmetry codes: (i) 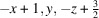; (iii) 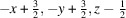; (iv) 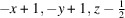; (v) 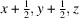; (vi) 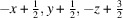; (vii) 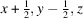; (viii) 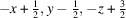.

**Table 9:** Hydrogen-bond geometry (Å, °) for **2**[Chem scheme1]

*D*—H⋯*A*	*D*—H	H⋯*A*	*D*⋯*A*	*D*—H⋯*A*
N11—H01⋯Br1	0.86 (7)	2.96 (8)	3.471 (8)	120 (6)
N11—H01⋯Br1^i^	0.86 (7)	2.83 (8)	3.612 (8)	153 (7)
C16—H16⋯Br2^ii^	0.95	2.96	3.699 (9)	136
C17—H17*A*⋯Br2^iii^	0.98	2.99	3.881 (9)	152
C18—H18*C*⋯Br2^iv^	0.98	2.99	3.948 (9)	165

Symmetry codes: (i) 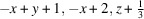; (ii) 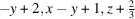; (iii) 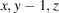; (iv) 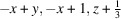.

**Table 10:** Hydrogen-bond geometry (Å, °) for **3a**[Chem scheme1]

*D*—H⋯*A*	*D*—H	H⋯*A*	*D*⋯*A*	*D*—H⋯*A*
N11—H01⋯Br3	0.87 (4)	2.42 (4)	3.234 (4)	158 (4)
N11—H01⋯Br3^ii^	0.87 (4)	2.42 (4)	3.234 (4)	158 (4)
C18—H18*A*⋯Br1^i^	0.98	2.93	3.862 (4)	159
C16—H16⋯Br2	0.95	3.00	3.704 (4)	132
C16—H16⋯Br1^i^	0.95	3.04	3.908 (4)	153

Symmetry codes: (i) 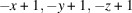; (ii) 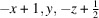.

**Table 11:** Hydrogen-bond geometry (Å, °) for **3b**[Chem scheme1]

*D*—H⋯*A*	*D*—H	H⋯*A*	*D*⋯*A*	*D*—H⋯*A*
N11—H01⋯Br10	0.77 (4)	2.49 (5)	3.215 (7)	157 (8)
N21—H02⋯Br9	0.78 (4)	2.46 (4)	3.220 (7)	168 (8)
N31—H03⋯Br9	0.77 (4)	2.47 (5)	3.203 (6)	159 (7)
N41—H04⋯Br10	0.77 (4)	2.45 (4)	3.218 (6)	172 (8)
C12—H12⋯Br9^i^	0.95	2.83	3.672 (8)	148
C16—H16⋯Br5	0.95	2.97	3.666 (8)	131
C22—H22⋯Br10^ii^	0.95	2.81	3.470 (7)	127
C26—H26⋯Br6	0.95	2.81	3.718 (8)	160
C32—H32⋯Br4^ii^	0.95	2.97	3.882 (7)	162
C36—H36⋯Br6	0.95	3.02	3.697 (7)	130
C42—H42⋯Br2^iii^	0.95	3.09	3.896 (8)	143
C17—H17*C*⋯Br3^iv^	0.98	2.87	3.680 (7)	141
C27—H27*B*⋯Br3	0.98	2.90	3.666 (8)	136
C28—H28*A*⋯Br4	0.98	3.01	3.809 (7)	140
C48—H48*B*⋯Br6^iv^	0.98	3.02	3.694 (8)	127
C48—H48*A*⋯Br8	0.98	3.00	3.744 (8)	133
C18—H18*A*⋯Br9	0.98	2.98	3.955 (8)	174
C24—H24⋯Br9^i^	0.95	2.92	3.714 (7)	142
C14—H14⋯Br10^ii^	0.95	2.93	3.792 (7)	152

Symmetry codes: (i) 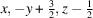; (ii) 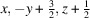; (iii) 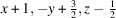; (iv) 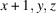.

**Table 12:** Hydrogen-bond geometry (Å, °) for **4**[Chem scheme1]

*D*—H⋯*A*	*D*—H	H⋯*A*	*D*⋯*A*	*D*—H⋯*A*
N11—H01⋯Br9	0.83 (3)	2.40 (3)	3.217 (5)	166 (5)
N21—H02⋯Br9	0.84 (3)	2.50 (4)	3.286 (4)	157 (5)
N31—H03⋯Br9	0.83 (3)	2.53 (4)	3.246 (5)	145 (5)
C12—H12⋯Br7	0.95	2.95	3.740 (6)	141
C32—H32⋯Br6	0.95	2.87	3.769 (5)	159
C18—H18*C*⋯Br6^i^	0.98	3.00	3.942 (6)	162
C27—H27*A*⋯Br8^iii^	0.98	2.99	3.822 (5)	143
C34—H34⋯Br1^iv^	0.95	3.01	3.957 (5)	172

Symmetry codes: (i) 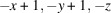; (iii) 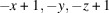; (iv) 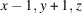.

**Table:** 

	**1a**	**1b**	**2**
Crystal data			
Chemical formula	[AuBr_2_(C_7_H_9_N)_2_](Br_3_)	[AuBr_2_(C_7_H_9_N)_2_](Br_3_)	(C_7_H_10_N)[AuBr_4_]
*M*_r_	810.82	810.82	624.77
Crystal system, space group	Triclinic, *P* 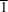	Orthorhombic, *C*222_1_	Trigonal, *P*3_2_
Temperature (K)	100	100	100
*a*, *b*, *c* (Å)	7.4459 (4), 8.9211 (6), 9.4090 (6)	9.42043 (16), 15.8371 (2), 13.7492 (2)	10.0289 (4), 10.0289 (4), 11.2031 (5)
α, β, γ (°)	106.488 (6), 101.605 (5), 112.778 (7)	90, 90, 90	90, 90, 120
*V* (Å^3^)	517.23 (6)	2051.28 (6)	975.83 (9)
*Z*	1	4	3
Radiation type	Mo *K*α	Mo *K*α	Mo *K*α
μ (mm^−1^)	16.77	16.91	23.55
Crystal size (mm)	0.20 × 0.18 × 0.15	0.2 × 0.1 × 0.05	0.1 × 0.05 × 0.01

Data collection			
Diffractometer	Oxford Diffraction Xcalibur, Eos	Oxford Diffraction Xcalibur, Eos	Oxford Diffraction Xcalibur, Eos
Absorption correction	Multi-scan (*CrysAlis PRO*; Rigaku OD, 2014 ▸)	Multi-scan (*CrysAlis PRO*; Rigaku OD, 2014 ▸)	Multi-scan (*CrysAlis PRO*; Rigaku OD, 2014 ▸)
*T*_min_, *T*_max_	0.321, 1.000	0.234, 1.000	0.413, 1.000
No. of measured, independent and observed [*I* > 2σ(*I*)] reflections	27639, 3076, 2938	112533, 3158, 3084	25086, 3312, 2979
*R*_int_	0.033	0.052	0.071
θ values (°)	θ_max_ = 31.0, θ_min_ = 2.4	θ_max_ = 31.1, θ_min_ = 2.5	θ_max_ = 29.3, θ_min_ = 2.4
(sin θ/λ)_max_ (Å^−1^)	0.725	0.727	0.688

Refinement			
*R*[*F*^2^ > 2σ(*F*^2^)], *wR*(*F*^2^), *S*	0.019, 0.040, 1.10	0.018, 0.037, 1.07	0.027, 0.036, 0.97
No. of reflections	3076	3158	3312
No. of parameters	106	105	124
No. of restraints	0	0	1
H-atom treatment	H-atom parameters constrained	H-atom parameters constrained	H atoms treated by a mixture of independent and constrained refinement
Δρ_max_, Δρ_min_ (e Å^−3^)	1.39, −0.97	1.37, −1.05	0.98, −1.17
Extinction method	*F*_c_^*^ = *kF*_c_[1+0.001*xF*_c_^2^λ^3^/sin(2θ)]^-1/4^ (*SHELXL2019/3*; Sheldrick, 2015 ▸)	None	None
Extinction coefficient	0.0035 (2)	–	–
Absolute structure	–	Flack *x* determined using 1294 quotients [(*I*^+^)−(*I*^−^)]/[(*I*^+^)+(*I*^−^)] (Parsons *et al.*, 2013 ▸)	Flack *x* determined using 1316 quotients [(*I*^+^)−(*I*^−^)]/[(*I*^+^)+(*I*^−^)] (Parsons *et al.*, 2013 ▸)
Absolute structure parameter	–	−0.028 (3)	−0.026 (8)

**Table:** 

	**3a**	**3b**	**4**
Crystal data			
Chemical formula	(C_7_H_10_N)_2_[AuBr_4_]Br	(C_7_H_10_N)_2_[AuBr_4_]Br	(C_7_H_10_N)_3_[AuBr_4_]_2_Br
*M*_r_	812.84	812.84	1437.60
Crystal system, space group	Monoclinic, *C*2/*c*	Monoclinic, *P*2_1_/*c*	Triclinic, *P* 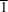
Temperature (K)	100	100	100
*a*, *b*, *c* (Å)	15.56307 (10), 9.5783 (4), 16.2371 (11)	10.5702 (4), 24.8303 (11), 16.4823 (7)	8.1261 (4), 12.0375 (5), 18.0076 (11)
α, β, γ (°)	90, 119.625 (9), 90	90, 98.256 (4), 90	90.532 (4), 94.151 (5), 103.359 (4)
*V* (Å^3^)	2104.0 (2)	4281.1 (3)	1708.68 (15)
*Z*	4	8	2
Radiation type	Mo *K*α	Mo *K*α	Mo *K*α
μ (mm^−1^)	16.49	16.20	19.12
Crystal size (mm)	0.1 × 0.05 × 0.03	0.11 × 0.11 × 0.03	0.15 × 0.10 × 0.05

Data collection			
Diffractometer	Oxford Diffraction Xcalibur, Eos	Oxford Diffraction Xcalibur, Eos	Oxford Diffraction Xcalibur, Eos
Absorption correction	Multi-scan (*CrysAlis PRO*; Rigaku OD, 2014 ▸)	Multi-scan (*CrysAlis PRO*; Rigaku OD, 2014 ▸)	Multi-scan (*CrysAlis PRO*; Rigaku OD, 2014 ▸)
*T*_min_, *T*_max_	0.426, 1.000	0.330, 1.000	0.256, 1.000
No. of measured, independent and observed [*I* > 2σ(*I*)] reflections	29892, 3043, 2496	206035, 10607, 7996	86859, 8153, 6751
*R*_int_	0.080	0.171	0.088
θ values (°)	θ_max_ = 30.0, θ_min_ = 2.6	θ_max_ = 28.3, θ_min_ = 2.1	θ_max_ = 27.9, θ_min_ = 2.3
(sin θ/λ)_max_ (Å^−1^)	0.704	0.667	0.658

Refinement			
*R*[*F*^2^ > 2σ(*F*^2^)], *wR*(*F*^2^), *S*	0.030, 0.046, 1.08	0.048, 0.079, 1.05	0.031, 0.053, 1.03
No. of reflections	3043	10607	8153
No. of parameters	109	418	336
No. of restraints	0	6	3
H-atom treatment	H atoms treated by a mixture of independent and constrained refinement	H atoms treated by a mixture of independent and constrained refinement	H atoms treated by a mixture of independent and constrained refinement
Δρ_max_, Δρ_min_ (e Å^−3^)	0.95, −0.92	1.38, −1.11	1.33, −1.16
Extinction method	*F*_c_^*^ = *kF*_c_[1+0.001*xF*_c_^2^λ^3^/sin(2θ)]^-1/4^ (*SHELXL2019/3*; Sheldrick, 2015 ▸)	None	*F*_c_^*^ = *kF*_c_[1+0.001*xF*_c_^2^λ^3^/sin(2θ)]^-1/4^ (*SHELXL2019/3*; Sheldrick, 2015 ▸)
Extinction coefficient	0.00018 (2)	–	0.00037 (2)

Computer programs: *CrysAlis PRO* (Rigaku OD, 2014 ▸, 2024 ▸), *SHELXS97* (Sheldrick, 2008 ▸), *SHELXL2019/3* (Sheldrick, 2015 ▸), *XP* (Bruker, 1998 ▸) and *publCIF* (Westrip, 2010 ▸).

## supplementary crystallographic information

### trans-Dibromidobis(3,5-dimethylpyridine)gold(III) tribromide (1a) Crystal data

**Table:**

[AuBr_2_(C_7_H_9_N)_2_](Br_3_)	*Z* = 1
*M**_r_* = 810.82	*F*(000) = 370
Triclinic, *P*1	*D*_x_ = 2.603 Mg m^−^^3^
*a* = 7.4459 (4) Å	Mo *K*α radiation, λ = 0.71073 Å
*b* = 8.9211 (6) Å	Cell parameters from 13952 reflections
*c* = 9.4090 (6) Å	θ = 2.7–30.8°
α = 106.488 (6)°	µ = 16.77 mm^−^^1^
β = 101.605 (5)°	*T* = 100 K
γ = 112.778 (7)°	Block, orange
*V* = 517.23 (6) Å^3^	0.20 × 0.18 × 0.15 mm

### trans-Dibromidobis(3,5-dimethylpyridine)gold(III) tribromide (1a) Data collection

**Table:**

Oxford Diffraction Xcalibur, Eos diffractometer	3076 independent reflections
Radiation source: Enhance (Mo) X-ray Source	2938 reflections with *I* > 2σ(*I*)
Graphite monochromator	*R*_int_ = 0.033
Detector resolution: 16.1419 pixels mm^-1^	θ_max_ = 31.0°, θ_min_ = 2.4°
ω scan	*h* = −10→10
Absorption correction: multi-scan (CrysAlisPro; Rigaku OD, 2014)	*k* = −12→12
*T*_min_ = 0.321, *T*_max_ = 1.000	*l* = −13→13
27639 measured reflections	

### trans-Dibromidobis(3,5-dimethylpyridine)gold(III) tribromide (1a) Refinement

**Table:**

Refinement on *F*^2^	Secondary atom site location: difference Fourier map
Least-squares matrix: full	Hydrogen site location: inferred from neighbouring sites
*R*[*F*^2^ > 2σ(*F*^2^)] = 0.019	H-atom parameters constrained
*wR*(*F*^2^) = 0.040	*w* = 1/[σ^2^(*F*_o_^2^) + (0.0144*P*)^2^ + 0.7764*P*] where *P* = (*F*_o_^2^ + 2*F*_c_^2^)/3
*S* = 1.10	(Δ/σ)_max_ < 0.001
3076 reflections	Δρ_max_ = 1.39 e Å^−^^3^
106 parameters	Δρ_min_ = −0.97 e Å^−^^3^
0 restraints	Extinction correction: *SHELXL2019/3* (Sheldrick, 2015), *F*_c_^*^ = *kF*_c_[1 + 0.001*xF*_c_^2^λ^3^/sin(2θ)]^-1/4^
Primary atom site location: structure-invariant direct methods	Extinction coefficient: 0.0035 (2)

### trans-Dibromidobis(3,5-dimethylpyridine)gold(III) tribromide (1a) Fractional atomic coordinates and isotropic or equivalent isotropic displacement parameters (Å^2^)

**Table:**

	*x*	*y*	*z*	*U*_iso_*/*U*_eq_	
Au1	0.500000	0.500000	0.500000	0.01066 (5)	
Br1	0.17269 (4)	0.44609 (3)	0.32851 (3)	0.01908 (7)	
Br2	0.500000	0.500000	1.000000	0.01572 (8)	
Br3	0.32443 (5)	0.59580 (4)	0.80950 (3)	0.02047 (7)	
N11	0.3743 (3)	0.2408 (3)	0.4650 (3)	0.0132 (4)	
C12	0.3651 (4)	0.1989 (3)	0.5902 (3)	0.0150 (5)	
H12	0.422929	0.291356	0.692657	0.018*	
C13	0.2730 (4)	0.0236 (4)	0.5740 (4)	0.0175 (5)	
C14	0.1918 (4)	−0.1078 (4)	0.4231 (4)	0.0205 (6)	
H14	0.126709	−0.228790	0.408368	0.025*	
C15	0.2037 (5)	−0.0654 (4)	0.2928 (4)	0.0200 (6)	
C16	0.2975 (4)	0.1132 (3)	0.3189 (3)	0.0165 (5)	
H16	0.307428	0.145490	0.231733	0.020*	
C17	0.2655 (5)	−0.0170 (4)	0.7182 (4)	0.0260 (7)	
H17A	0.127151	−0.047635	0.725360	0.039*	
H17B	0.370612	0.087284	0.813223	0.039*	
H17C	0.293699	−0.117159	0.709784	0.039*	
C18	0.1206 (6)	−0.2049 (4)	0.1277 (4)	0.0325 (8)	
H18A	0.194472	−0.275274	0.123554	0.049*	
H18B	0.141378	−0.147602	0.053318	0.049*	
H18C	−0.028361	−0.282757	0.098607	0.049*	

### trans-Dibromidobis(3,5-dimethylpyridine)gold(III) tribromide (1a) Atomic displacement parameters (Å^2^)

**Table:**

	*U*^11^	*U*^22^	*U*^33^	*U*^12^	*U*^13^	*U*^23^
Au1	0.00999 (8)	0.00759 (7)	0.01247 (7)	0.00381 (5)	0.00169 (5)	0.00352 (5)
Br1	0.01447 (14)	0.01466 (12)	0.02314 (14)	0.00637 (11)	−0.00075 (11)	0.00679 (10)
Br2	0.01613 (19)	0.01974 (18)	0.01447 (17)	0.00995 (15)	0.00661 (14)	0.00818 (14)
Br3	0.02277 (16)	0.02845 (15)	0.02051 (14)	0.01737 (13)	0.00949 (11)	0.01481 (12)
N11	0.0114 (11)	0.0107 (10)	0.0165 (11)	0.0058 (9)	0.0035 (8)	0.0043 (8)
C12	0.0126 (13)	0.0121 (11)	0.0211 (13)	0.0064 (10)	0.0058 (10)	0.0070 (10)
C13	0.0109 (13)	0.0158 (13)	0.0297 (15)	0.0078 (11)	0.0081 (11)	0.0115 (11)
C14	0.0115 (14)	0.0103 (12)	0.0402 (17)	0.0053 (11)	0.0096 (12)	0.0100 (12)
C15	0.0138 (14)	0.0129 (12)	0.0273 (15)	0.0063 (11)	0.0049 (11)	0.0016 (11)
C16	0.0136 (14)	0.0134 (12)	0.0190 (13)	0.0064 (11)	0.0042 (10)	0.0029 (10)
C17	0.0258 (17)	0.0249 (15)	0.0410 (19)	0.0141 (14)	0.0173 (14)	0.0245 (14)
C18	0.0296 (19)	0.0170 (14)	0.0327 (18)	0.0049 (13)	0.0077 (15)	−0.0036 (13)

### trans-Dibromidobis(3,5-dimethylpyridine)gold(III) tribromide (1a) Geometric parameters (Å, º)

**Table:**

Au1—N11^i^	2.025 (2)	C14—C15	1.391 (4)
Au1—N11	2.025 (2)	C14—H14	0.9500
Au1—Br1^i^	2.4174 (3)	C15—C16	1.391 (4)
Au1—Br1	2.4174 (3)	C15—C18	1.505 (4)
Br2—Br3^ii^	2.5385 (3)	C16—H16	0.9500
Br2—Br3	2.5385 (3)	C17—H17A	0.9800
N11—C12	1.340 (3)	C17—H17B	0.9800
N11—C16	1.343 (3)	C17—H17C	0.9800
C12—C13	1.389 (4)	C18—H18A	0.9800
C12—H12	0.9500	C18—H18B	0.9800
C13—C14	1.384 (4)	C18—H18C	0.9800
C13—C17	1.507 (4)		
			
N11^i^—Au1—N11	180.0	C16—C15—C14	117.8 (3)
N11^i^—Au1—Br1^i^	90.01 (6)	C16—C15—C18	119.8 (3)
N11—Au1—Br1^i^	89.99 (6)	C14—C15—C18	122.4 (3)
N11^i^—Au1—Br1	89.99 (6)	N11—C16—C15	121.2 (3)
N11—Au1—Br1	90.01 (6)	N11—C16—H16	119.4
Br1^i^—Au1—Br1	180.0	C15—C16—H16	119.4
Br3^ii^—Br2—Br3	180.0	C13—C17—H17A	109.5
C12—N11—C16	120.6 (2)	C13—C17—H17B	109.5
C12—N11—Au1	118.62 (18)	H17A—C17—H17B	109.5
C16—N11—Au1	120.76 (18)	C13—C17—H17C	109.5
N11—C12—C13	121.7 (3)	H17A—C17—H17C	109.5
N11—C12—H12	119.1	H17B—C17—H17C	109.5
C13—C12—H12	119.1	C15—C18—H18A	109.5
C14—C13—C12	117.6 (3)	C15—C18—H18B	109.5
C14—C13—C17	122.6 (3)	H18A—C18—H18B	109.5
C12—C13—C17	119.8 (3)	C15—C18—H18C	109.5
C13—C14—C15	121.1 (3)	H18A—C18—H18C	109.5
C13—C14—H14	119.5	H18B—C18—H18C	109.5
C15—C14—H14	119.5		
			
C16—N11—C12—C13	−1.3 (4)	C13—C14—C15—C16	−0.9 (4)
Au1—N11—C12—C13	177.3 (2)	C13—C14—C15—C18	178.8 (3)
N11—C12—C13—C14	0.5 (4)	C12—N11—C16—C15	1.0 (4)
N11—C12—C13—C17	−179.7 (3)	Au1—N11—C16—C15	−177.5 (2)
C12—C13—C14—C15	0.6 (4)	C14—C15—C16—N11	0.1 (4)
C17—C13—C14—C15	−179.2 (3)	C18—C15—C16—N11	−179.6 (3)

Symmetry codes: (i) −*x*+1, −*y*+1, −*z*+1; (ii) −*x*+1, −*y*+1, −*z*+2.

### trans-Dibromidobis(3,5-dimethylpyridine)gold(III) tribromide (1a) Hydrogen-bond geometry (Å, º)

**Table:**

*D*—H···*A*	*D*—H	H···*A*	*D*···*A*	*D*—H···*A*
C14—H14···Br1^iii^	0.95	2.95	3.762 (3)	144
C12—H12···Br2	0.95	2.76	3.683 (3)	165
C12—H12···Br3	0.95	3.05	3.718 (3)	129
C16—H16···Br3^i^	0.95	3.00	3.689 (3)	130
C17—H17*C*···Br3^iii^	0.98	3.04	3.946 (3)	155

Symmetry codes: (i) −*x*+1, −*y*+1, −*z*+1; (iii) *x*, *y*−1, *z*.

### trans-Dibromidobis(3,5-dimethylpyridine)gold(III) tribromide (1b) Crystal data

**Table:**

[AuBr_2_(C_7_H_9_N)_2_](Br_3_)	*D*_x_ = 2.625 Mg m^−^^3^
*M**_r_* = 810.82	Mo *K*α radiation, λ = 0.71073 Å
Orthorhombic, *C*222_1_	Cell parameters from 42051 reflections
*a* = 9.42043 (16) Å	θ = 2.5–30.7°
*b* = 15.8371 (2) Å	µ = 16.91 mm^−^^1^
*c* = 13.7492 (2) Å	*T* = 100 K
*V* = 2051.28 (6) Å^3^	Plate, orange
*Z* = 4	0.2 × 0.1 × 0.05 mm
*F*(000) = 1480	

### trans-Dibromidobis(3,5-dimethylpyridine)gold(III) tribromide (1b) Data collection

**Table:**

Oxford Diffraction Xcalibur, Eos diffractometer	3158 independent reflections
Radiation source: Enhance (Mo) X-ray Source	3084 reflections with *I* > 2σ(*I*)
Graphite monochromator	*R*_int_ = 0.052
Detector resolution: 16.1419 pixels mm^-1^	θ_max_ = 31.1°, θ_min_ = 2.5°
ω scan	*h* = −13→13
Absorption correction: multi-scan (CrysAlisPro; Rigaku OD, 2014)	*k* = −22→22
*T*_min_ = 0.234, *T*_max_ = 1.000	*l* = −19→19
112533 measured reflections	

### trans-Dibromidobis(3,5-dimethylpyridine)gold(III) tribromide (1b) Refinement

**Table:**

Refinement on *F*^2^	Secondary atom site location: difference Fourier map
Least-squares matrix: full	Hydrogen site location: inferred from neighbouring sites
*R*[*F*^2^ > 2σ(*F*^2^)] = 0.018	H-atom parameters constrained
*wR*(*F*^2^) = 0.037	*w* = 1/[σ^2^(*F*_o_^2^) + (0.0159*P*)^2^ + 7.964*P*] where *P* = (*F*_o_^2^ + 2*F*_c_^2^)/3
*S* = 1.07	(Δ/σ)_max_ = 0.002
3158 reflections	Δρ_max_ = 1.37 e Å^−^^3^
105 parameters	Δρ_min_ = −1.05 e Å^−^^3^
0 restraints	Absolute structure: Flack *x* determined using 1294 quotients [(*I*^+^)-(*I*^-^)]/[(*I*^+^)+(*I*^-^)] (Parsons *et al.*, 2013)
Primary atom site location: structure-invariant direct methods	Absolute structure parameter: −0.028 (3)

### trans-Dibromidobis(3,5-dimethylpyridine)gold(III) tribromide (1b) Fractional atomic coordinates and isotropic or equivalent isotropic displacement parameters (Å^2^)

**Table:**

	*x*	*y*	*z*	*U*_iso_*/*U*_eq_	
Au1	0.500000	0.59331 (2)	0.750000	0.00987 (5)	
Br1	0.59023 (5)	0.59396 (3)	0.91394 (3)	0.01749 (9)	
Br2	0.15342 (6)	0.500000	1.000000	0.01598 (12)	
Br3	0.15449 (5)	0.59671 (3)	0.85274 (4)	0.02405 (10)	
N11	0.500000	0.7209 (3)	0.750000	0.0119 (8)	
C12	0.5815 (5)	0.7630 (3)	0.6858 (3)	0.0128 (8)	
H12	0.638182	0.732117	0.641157	0.015*	
C13	0.5845 (5)	0.8506 (3)	0.6834 (3)	0.0136 (8)	
C14	0.500000	0.8937 (3)	0.750000	0.0141 (10)	
H14	0.499998	0.953642	0.749999	0.017*	
C15	0.6752 (5)	0.8955 (3)	0.6110 (4)	0.0216 (10)	
H15A	0.765415	0.865283	0.603593	0.032*	
H15B	0.693697	0.953103	0.633602	0.032*	
H15C	0.626148	0.897448	0.548185	0.032*	
N21	0.500000	0.4650 (3)	0.750000	0.0125 (8)	
C22	0.5727 (5)	0.4235 (2)	0.6806 (3)	0.0138 (8)	
H22	0.622294	0.454632	0.632306	0.017*	
C23	0.5763 (5)	0.3360 (3)	0.6783 (4)	0.0154 (9)	
C24	0.500000	0.2927 (3)	0.750000	0.0170 (11)	
H24	0.500000	0.232732	0.750000	0.020*	
C25	0.6583 (6)	0.2903 (3)	0.6013 (4)	0.0224 (10)	
H25A	0.597580	0.247472	0.571077	0.034*	
H25B	0.741207	0.263002	0.630605	0.034*	
H25C	0.689726	0.330681	0.551699	0.034*	

### trans-Dibromidobis(3,5-dimethylpyridine)gold(III) tribromide (1b) Atomic displacement parameters (Å^2^)

**Table:**

	*U*^11^	*U*^22^	*U*^33^	*U*^12^	*U*^13^	*U*^23^
Au1	0.01356 (9)	0.00659 (8)	0.00946 (9)	0.000	−0.00054 (8)	0.000
Br1	0.0247 (2)	0.01420 (17)	0.01352 (19)	0.00057 (18)	−0.00435 (15)	0.00044 (17)
Br2	0.0119 (3)	0.0137 (3)	0.0224 (3)	0.000	0.000	0.0011 (2)
Br3	0.0239 (2)	0.0194 (2)	0.0289 (2)	−0.0031 (2)	−0.00906 (18)	0.0081 (2)
N11	0.014 (2)	0.0098 (18)	0.012 (2)	0.000	−0.002 (3)	0.000
C12	0.0124 (19)	0.015 (2)	0.011 (2)	0.0005 (15)	0.0018 (15)	0.0000 (15)
C13	0.0129 (19)	0.0137 (18)	0.014 (2)	−0.0012 (15)	−0.0011 (16)	0.0021 (15)
C14	0.014 (2)	0.008 (2)	0.020 (3)	0.000	−0.004 (3)	0.000
C15	0.021 (2)	0.019 (2)	0.025 (2)	−0.0048 (18)	0.0028 (18)	0.0071 (18)
N21	0.015 (2)	0.0101 (18)	0.012 (2)	0.000	0.001 (3)	0.000
C22	0.016 (2)	0.011 (2)	0.015 (2)	0.0018 (14)	0.0006 (16)	−0.0002 (14)
C23	0.018 (2)	0.0113 (18)	0.017 (2)	0.0032 (15)	−0.0053 (17)	−0.0022 (15)
C24	0.022 (3)	0.010 (2)	0.019 (3)	0.000	−0.008 (3)	0.000
C25	0.029 (2)	0.016 (2)	0.023 (2)	0.0058 (18)	0.003 (2)	−0.0044 (18)

### trans-Dibromidobis(3,5-dimethylpyridine)gold(III) tribromide (1b) Geometric parameters (Å, º)

**Table:**

Au1—N11	2.020 (4)	C15—H15A	0.9800
Au1—N21	2.032 (4)	C15—H15B	0.9800
Au1—Br1^i^	2.4090 (4)	C15—H15C	0.9800
Au1—Br1	2.4090 (4)	N21—C22^i^	1.346 (5)
Br2—Br3^ii^	2.5387 (5)	N21—C22	1.346 (5)
Br2—Br3	2.5388 (5)	C22—C23	1.386 (5)
N11—C12^i^	1.347 (5)	C22—H22	0.9500
N11—C12	1.347 (5)	C23—C24	1.399 (5)
C12—C13	1.387 (6)	C23—C25	1.497 (7)
C12—H12	0.9500	C24—H24	0.9500
C13—C14	1.392 (5)	C25—H25A	0.9800
C13—C15	1.492 (6)	C25—H25B	0.9800
C14—H14	0.9500	C25—H25C	0.9800
			
N11—Au1—N21	180.0	C13—C15—H15C	109.5
N11—Au1—Br1^i^	89.753 (12)	H15A—C15—H15C	109.5
N21—Au1—Br1^i^	90.247 (12)	H15B—C15—H15C	109.5
N11—Au1—Br1	89.753 (12)	C22^i^—N21—C22	121.5 (5)
N21—Au1—Br1	90.247 (12)	C22^i^—N21—Au1	119.3 (2)
Br1^i^—Au1—Br1	179.51 (2)	C22—N21—Au1	119.3 (2)
Br3^ii^—Br2—Br3	179.55 (3)	N21—C22—C23	121.1 (4)
C12^i^—N11—C12	120.5 (5)	N21—C22—H22	119.4
C12^i^—N11—Au1	119.7 (2)	C23—C22—H22	119.4
C12—N11—Au1	119.7 (2)	C22—C23—C24	117.5 (4)
N11—C12—C13	121.5 (4)	C22—C23—C25	120.7 (4)
N11—C12—H12	119.2	C24—C23—C25	121.8 (4)
C13—C12—H12	119.2	C23^i^—C24—C23	121.3 (5)
C12—C13—C14	117.5 (4)	C23^i^—C24—H24	119.3
C12—C13—C15	120.2 (4)	C23—C24—H24	119.3
C14—C13—C15	122.2 (4)	C23—C25—H25A	109.5
C13—C14—C13^i^	121.3 (5)	C23—C25—H25B	109.5
C13—C14—H14	119.3	H25A—C25—H25B	109.5
C13^i^—C14—H14	119.3	C23—C25—H25C	109.5
C13—C15—H15A	109.5	H25A—C25—H25C	109.5
C13—C15—H15B	109.5	H25B—C25—H25C	109.5
H15A—C15—H15B	109.5		
			
C12^i^—N11—C12—C13	0.0 (3)	C22^i^—N21—C22—C23	−0.5 (3)
Au1—N11—C12—C13	−180.0 (3)	Au1—N21—C22—C23	179.5 (3)
N11—C12—C13—C14	0.0 (6)	N21—C22—C23—C24	0.9 (6)
N11—C12—C13—C15	−179.6 (4)	N21—C22—C23—C25	−179.5 (4)
C12—C13—C14—C13^i^	0.0 (3)	C22—C23—C24—C23^i^	−0.4 (3)
C15—C13—C14—C13^i^	179.6 (5)	C25—C23—C24—C23^i^	−180.0 (5)

Symmetry codes: (i) −*x*+1, *y*, −*z*+3/2; (ii) *x*, −*y*+1, −*z*+2.

### trans-Dibromidobis(3,5-dimethylpyridine)gold(III) tribromide (1b) Hydrogen-bond geometry (Å, º)

**Table:**

*D*—H···*A*	*D*—H	H···*A*	*D*···*A*	*D*—H···*A*
C15—H15*A*···Br1^iii^	0.98	3.01	3.500 (5)	112
C22—H22···Br1^i^	0.95	3.05	3.367 (4)	102
C22—H22···Br2^iv^	0.95	2.88	3.780 (4)	159
C12—H12···Br3^i^	0.95	2.90	3.662 (4)	138
C14—H14···Br3^v^	0.95	3.04	3.802 (4)	138
C14—H14···Br3^vi^	0.95	3.04	3.802 (4)	138
C24—H24···Br3^vii^	0.95	2.96	3.708 (4)	137
C24—H24···Br3^viii^	0.95	2.96	3.708 (4)	137

Symmetry codes: (i) −*x*+1, *y*, −*z*+3/2; (iii) −*x*+3/2, −*y*+3/2, *z*−1/2; (iv) −*x*+1, −*y*+1, *z*−1/2; (v) *x*+1/2, *y*+1/2, *z*; (vi) −*x*+1/2, *y*+1/2, −*z*+3/2; (vii) *x*+1/2, *y*−1/2, *z*; (viii) −*x*+1/2, *y*−1/2, −*z*+3/2.

### 3,5-Dimethylpyridinium tetrabromidoaurate(III) (2) Crystal data

**Table:**

(C_7_H_10_N)[AuBr_4_]	*D*_x_ = 3.189 Mg m^−^^3^
*M**_r_* = 624.77	Mo *K*α radiation, λ = 0.71073 Å
Trigonal, *P*3_2_	Cell parameters from 6101 reflections
*a* = 10.0289 (4) Å	θ = 3.0–28.4°
*c* = 11.2031 (5) Å	µ = 23.55 mm^−^^1^
*V* = 975.83 (9) Å^3^	*T* = 100 K
*Z* = 3	Plate, red
*F*(000) = 834	0.1 × 0.05 × 0.01 mm

### 3,5-Dimethylpyridinium tetrabromidoaurate(III) (2) Data collection

**Table:**

Oxford Diffraction Xcalibur, Eos diffractometer	3312 independent reflections
Radiation source: Enhance (Mo) X-ray Source	2979 reflections with *I* > 2σ(*I*)
Graphite monochromator	*R*_int_ = 0.071
Detector resolution: 16.1419 pixels mm^-1^	θ_max_ = 29.3°, θ_min_ = 2.4°
ω scan	*h* = −13→13
Absorption correction: multi-scan (CrysAlisPro; Rigaku OD, 2014)	*k* = −13→13
*T*_min_ = 0.413, *T*_max_ = 1.000	*l* = −15→14
25086 measured reflections	

### 3,5-Dimethylpyridinium tetrabromidoaurate(III) (2) Refinement

**Table:**

Refinement on *F*^2^	Secondary atom site location: difference Fourier map
Least-squares matrix: full	Hydrogen site location: mixed
*R*[*F*^2^ > 2σ(*F*^2^)] = 0.027	H atoms treated by a mixture of independent and constrained refinement
*wR*(*F*^2^) = 0.036	*w* = 1/[σ^2^(*F*_o_^2^) + (0.0044*P*)^2^] where *P* = (*F*_o_^2^ + 2*F*_c_^2^)/3
*S* = 0.97	(Δ/σ)_max_ = 0.004
3312 reflections	Δρ_max_ = 0.98 e Å^−^^3^
124 parameters	Δρ_min_ = −1.17 e Å^−^^3^
1 restraint	Absolute structure: Flack *x* determined using 1316 quotients [(*I*^+^)-(*I*^-^)]/[(*I*^+^)+(*I*^-^)] (Parsons *et al.*, 2013)
Primary atom site location: structure-invariant direct methods	Absolute structure parameter: −0.026 (8)

### 3,5-Dimethylpyridinium tetrabromidoaurate(III) (2) Fractional atomic coordinates and isotropic or equivalent isotropic displacement parameters (Å^2^)

**Table:**

	*x*	*y*	*z*	*U*_iso_*/*U*_eq_	
Au1	0.74829 (4)	0.96276 (4)	0.49944 (3)	0.01152 (7)	
Br1	0.94551 (11)	0.90783 (11)	0.43069 (7)	0.0161 (2)	
Br2	0.86802 (11)	1.19711 (11)	0.38173 (8)	0.0191 (2)	
Br3	0.54730 (12)	1.01487 (12)	0.55997 (9)	0.0265 (2)	
Br4	0.63343 (11)	0.73390 (10)	0.62398 (7)	0.0154 (2)	
N11	1.0081 (10)	0.7334 (9)	0.6751 (8)	0.019 (2)	
H01	0.996 (9)	0.812 (9)	0.668 (7)	0.00 (2)*	
C12	1.1063 (10)	0.7201 (10)	0.6020 (8)	0.016 (2)	
H12	1.177312	0.804675	0.554794	0.019*	
C13	1.1018 (11)	0.5793 (11)	0.5968 (8)	0.018 (2)	
C14	0.9901 (9)	0.4597 (11)	0.6632 (8)	0.014 (2)	
H14	0.983312	0.361941	0.658517	0.017*	
C15	0.8877 (10)	0.4752 (11)	0.7362 (8)	0.016 (2)	
C16	0.9007 (11)	0.6174 (12)	0.7404 (8)	0.019 (2)	
H16	0.833425	0.634088	0.789812	0.022*	
C17	1.2141 (10)	0.5614 (11)	0.5184 (8)	0.022 (2)	
H17A	1.158851	0.466390	0.471764	0.033*	
H17B	1.291811	0.556534	0.568365	0.033*	
H17C	1.264721	0.649656	0.464218	0.033*	
C18	0.7671 (10)	0.3425 (11)	0.8090 (8)	0.022 (2)	
H18A	0.768960	0.376505	0.891301	0.032*	
H18B	0.789129	0.257671	0.808583	0.032*	
H18C	0.665206	0.307406	0.774263	0.032*	

### 3,5-Dimethylpyridinium tetrabromidoaurate(III) (2) Atomic displacement parameters (Å^2^)

**Table:**

	*U*^11^	*U*^22^	*U*^33^	*U*^12^	*U*^13^	*U*^23^
Au1	0.01101 (18)	0.01116 (18)	0.01289 (14)	0.00591 (16)	0.00031 (16)	−0.00055 (15)
Br1	0.0165 (5)	0.0183 (5)	0.0169 (5)	0.0113 (4)	0.0047 (4)	0.0024 (4)
Br2	0.0213 (5)	0.0164 (5)	0.0205 (5)	0.0100 (5)	0.0033 (4)	0.0052 (4)
Br3	0.0203 (6)	0.0229 (6)	0.0433 (6)	0.0160 (5)	0.0116 (5)	0.0064 (5)
Br4	0.0147 (5)	0.0122 (5)	0.0180 (5)	0.0059 (4)	0.0019 (4)	0.0021 (4)
N11	0.025 (5)	0.016 (4)	0.023 (5)	0.016 (4)	0.000 (4)	0.003 (4)
C12	0.019 (5)	0.014 (5)	0.013 (5)	0.008 (5)	0.000 (4)	0.002 (4)
C13	0.020 (6)	0.019 (5)	0.013 (5)	0.009 (5)	−0.006 (4)	−0.004 (4)
C14	0.012 (5)	0.021 (6)	0.016 (4)	0.013 (4)	−0.008 (4)	−0.006 (4)
C15	0.014 (5)	0.023 (6)	0.011 (5)	0.011 (5)	−0.002 (4)	0.007 (4)
C16	0.023 (6)	0.025 (6)	0.009 (5)	0.012 (5)	−0.002 (4)	0.002 (4)
C17	0.021 (6)	0.024 (6)	0.020 (5)	0.012 (5)	0.003 (4)	−0.004 (4)
C18	0.018 (5)	0.031 (6)	0.021 (5)	0.017 (5)	0.007 (4)	0.009 (5)

### 3,5-Dimethylpyridinium tetrabromidoaurate(III) (2) Geometric parameters (Å, º)

**Table:**

Au1—Br3	2.4186 (10)	C14—C15	1.381 (12)
Au1—Br2	2.4254 (10)	C14—H14	0.9500
Au1—Br4	2.4285 (9)	C15—C16	1.366 (13)
Au1—Br1	2.4285 (10)	C15—C18	1.513 (12)
N11—C12	1.338 (11)	C16—H16	0.9500
N11—C16	1.340 (12)	C17—H17A	0.9800
N11—H01	0.86 (7)	C17—H17B	0.9800
C12—C13	1.391 (12)	C17—H17C	0.9800
C12—H12	0.9500	C18—H18A	0.9800
C13—C14	1.380 (13)	C18—H18B	0.9800
C13—C17	1.508 (12)	C18—H18C	0.9800
			
Br3—Au1—Br2	90.31 (4)	C16—C15—C14	116.7 (9)
Br3—Au1—Br4	89.45 (3)	C16—C15—C18	121.1 (9)
Br2—Au1—Br4	177.81 (4)	C14—C15—C18	122.2 (9)
Br3—Au1—Br1	177.69 (4)	N11—C16—C15	120.2 (9)
Br2—Au1—Br1	89.01 (3)	N11—C16—H16	119.9
Br4—Au1—Br1	91.32 (3)	C15—C16—H16	119.9
C12—N11—C16	124.0 (8)	C13—C17—H17A	109.5
C12—N11—H01	119 (6)	C13—C17—H17B	109.5
C16—N11—H01	115 (6)	H17A—C17—H17B	109.5
N11—C12—C13	118.4 (9)	C13—C17—H17C	109.5
N11—C12—H12	120.8	H17A—C17—H17C	109.5
C13—C12—H12	120.8	H17B—C17—H17C	109.5
C14—C13—C12	117.4 (9)	C15—C18—H18A	109.5
C14—C13—C17	122.7 (9)	C15—C18—H18B	109.5
C12—C13—C17	119.9 (9)	H18A—C18—H18B	109.5
C13—C14—C15	123.2 (9)	C15—C18—H18C	109.5
C13—C14—H14	118.4	H18A—C18—H18C	109.5
C15—C14—H14	118.4	H18B—C18—H18C	109.5
			
C16—N11—C12—C13	−2.9 (15)	C13—C14—C15—C16	0.2 (14)
N11—C12—C13—C14	3.3 (13)	C13—C14—C15—C18	−179.5 (8)
N11—C12—C13—C17	−178.0 (9)	C12—N11—C16—C15	0.9 (15)
C12—C13—C14—C15	−2.1 (14)	C14—C15—C16—N11	0.5 (13)
C17—C13—C14—C15	179.3 (8)	C18—C15—C16—N11	−179.9 (8)

### 3,5-Dimethylpyridinium tetrabromidoaurate(III) (2) Hydrogen-bond geometry (Å, º)

**Table:**

*D*—H···*A*	*D*—H	H···*A*	*D*···*A*	*D*—H···*A*
N11—H01···Br1	0.86 (7)	2.96 (8)	3.471 (8)	120 (6)
N11—H01···Br1^i^	0.86 (7)	2.83 (8)	3.612 (8)	153 (7)
C16—H16···Br2^ii^	0.95	2.96	3.699 (9)	136
C17—H17*A*···Br2^iii^	0.98	2.99	3.881 (9)	152
C18—H18*C*···Br2^iv^	0.98	2.99	3.948 (9)	165

Symmetry codes: (i) −*x*+*y*+1, −*x*+2, *z*+1/3; (ii) −*y*+2, *x*−*y*+1, *z*+2/3; (iii) *x*, *y*−1, *z*; (iv) −*x*+*y*, −*x*+1, *z*+1/3.

### Bis(3,5-dimethylpyridinium) tetrabromidoaurate(III) bromide (3a) Crystal data

**Table:**

(C_7_H_10_N)_2_[AuBr_4_]Br	*F*(000) = 1488
*M**_r_* = 812.84	*D*_x_ = 2.566 Mg m^−^^3^
Monoclinic, *C*2/*c*	Mo *K*α radiation, λ = 0.71073 Å
*a* = 15.56307 (10) Å	Cell parameters from 5779 reflections
*b* = 9.5783 (4) Å	θ = 2.6–27.4°
*c* = 16.2371 (11) Å	µ = 16.49 mm^−^^1^
β = 119.625 (9)°	*T* = 100 K
*V* = 2104.0 (2) Å^3^	Block, red
*Z* = 4	0.1 × 0.05 × 0.03 mm

### Bis(3,5-dimethylpyridinium) tetrabromidoaurate(III) bromide (3a) Data collection

**Table:**

Oxford Diffraction Xcalibur, Eos diffractometer	3043 independent reflections
Radiation source: fine-focus sealed X-ray tube	2496 reflections with *I* > 2σ(*I*)
Detector resolution: 16.1419 pixels mm^-1^	*R*_int_ = 0.080
ω scans	θ_max_ = 30.0°, θ_min_ = 2.6°
Absorption correction: multi-scan (CrysAlisPro; Rigaku OD, 2014)	*h* = −21→21
*T*_min_ = 0.426, *T*_max_ = 1.000	*k* = −13→13
29892 measured reflections	*l* = −22→22

### Bis(3,5-dimethylpyridinium) tetrabromidoaurate(III) bromide (3a) Refinement

**Table:**

Refinement on *F*^2^	Secondary atom site location: difference Fourier map
Least-squares matrix: full	Hydrogen site location: mixed
*R*[*F*^2^ > 2σ(*F*^2^)] = 0.030	H atoms treated by a mixture of independent and constrained refinement
*wR*(*F*^2^) = 0.046	*w* = 1/[σ^2^(*F*_o_^2^) + (0.0095*P*)^2^ + 1.2893*P*] where *P* = (*F*_o_^2^ + 2*F*_c_^2^)/3
*S* = 1.08	(Δ/σ)_max_ = 0.001
3043 reflections	Δρ_max_ = 0.95 e Å^−^^3^
109 parameters	Δρ_min_ = −0.92 e Å^−^^3^
0 restraints	Extinction correction: *SHELXL2019/3* (Sheldrick, 2015), *F*_c_^*^ = *kF*_c_[1 + 0.001*xF*_c_^2^λ^3^/sin(2θ)]^-1/4^
Primary atom site location: structure-invariant direct methods	Extinction coefficient: 0.00018 (2)

### Bis(3,5-dimethylpyridinium) tetrabromidoaurate(III) bromide (3a) Fractional atomic coordinates and isotropic or equivalent isotropic displacement parameters (Å^2^)

**Table:**

	*x*	*y*	*z*	*U*_iso_*/*U*_eq_	
Au1	0.500000	0.500000	0.500000	0.01572 (7)	
Br1	0.34420 (3)	0.59116 (5)	0.47583 (3)	0.03006 (12)	
Br2	0.49857 (3)	0.67295 (5)	0.39000 (3)	0.02193 (11)	
Br3	0.500000	0.96475 (6)	0.250000	0.01521 (13)	
N11	0.6983 (3)	0.7775 (4)	0.3312 (2)	0.0202 (8)	
H01	0.643 (3)	0.813 (5)	0.320 (3)	0.031 (14)*	
C12	0.7393 (3)	0.8298 (4)	0.2825 (3)	0.0215 (9)	
H12	0.708459	0.905566	0.240308	0.026*	
C13	0.8261 (3)	0.7748 (4)	0.2930 (3)	0.0189 (9)	
C14	0.8672 (3)	0.6653 (4)	0.3566 (3)	0.0156 (8)	
H14	0.927341	0.625631	0.365944	0.019*	
C15	0.8246 (3)	0.6112 (4)	0.4069 (3)	0.0162 (9)	
C16	0.7374 (3)	0.6715 (4)	0.3917 (3)	0.0191 (9)	
H16	0.705230	0.637376	0.424403	0.023*	
C17	0.8718 (3)	0.8299 (5)	0.2369 (3)	0.0273 (11)	
H17A	0.900660	0.752527	0.219336	0.041*	
H17B	0.923581	0.897448	0.275374	0.041*	
H17C	0.820901	0.875864	0.179472	0.041*	
C18	0.8688 (3)	0.4917 (5)	0.4745 (3)	0.0234 (10)	
H18A	0.817195	0.445186	0.482308	0.035*	
H18B	0.920308	0.526653	0.536010	0.035*	
H18C	0.897871	0.424960	0.449445	0.035*	

### Bis(3,5-dimethylpyridinium) tetrabromidoaurate(III) bromide (3a) Atomic displacement parameters (Å^2^)

**Table:**

	*U*^11^	*U*^22^	*U*^33^	*U*^12^	*U*^13^	*U*^23^
Au1	0.01979 (12)	0.01359 (12)	0.01490 (12)	−0.00205 (10)	0.00944 (9)	0.00040 (9)
Br1	0.0288 (3)	0.0269 (3)	0.0433 (3)	0.0071 (2)	0.0246 (2)	0.0133 (2)
Br2	0.0254 (2)	0.0213 (2)	0.0221 (2)	0.00071 (18)	0.01410 (19)	0.00706 (18)
Br3	0.0151 (3)	0.0145 (3)	0.0188 (3)	0.000	0.0105 (2)	0.000
N11	0.0151 (19)	0.022 (2)	0.023 (2)	0.0051 (16)	0.0085 (16)	−0.0019 (16)
C12	0.023 (2)	0.018 (2)	0.023 (2)	0.0050 (18)	0.012 (2)	0.0042 (18)
C13	0.022 (2)	0.016 (2)	0.018 (2)	0.0029 (18)	0.0101 (19)	−0.0002 (17)
C14	0.0107 (19)	0.017 (2)	0.015 (2)	0.0038 (16)	0.0034 (16)	−0.0026 (16)
C15	0.015 (2)	0.018 (2)	0.014 (2)	−0.0021 (17)	0.0058 (17)	−0.0014 (17)
C16	0.020 (2)	0.021 (2)	0.019 (2)	0.0011 (18)	0.0122 (19)	0.0003 (18)
C17	0.028 (2)	0.026 (3)	0.035 (3)	0.005 (2)	0.021 (2)	0.007 (2)
C18	0.020 (2)	0.027 (3)	0.024 (2)	0.004 (2)	0.0124 (19)	0.007 (2)

### Bis(3,5-dimethylpyridinium) tetrabromidoaurate(III) bromide (3a) Geometric parameters (Å, º)

**Table:**

Au1—Br1^i^	2.4197 (4)	C14—C15	1.383 (5)
Au1—Br1	2.4197 (4)	C14—H14	0.9500
Au1—Br2	2.4280 (4)	C15—C16	1.380 (5)
Au1—Br2^i^	2.4281 (4)	C15—C18	1.497 (5)
N11—C16	1.332 (5)	C16—H16	0.9500
N11—C12	1.334 (5)	C17—H17A	0.9800
N11—H01	0.87 (4)	C17—H17B	0.9800
C12—C13	1.380 (5)	C17—H17C	0.9800
C12—H12	0.9500	C18—H18A	0.9800
C13—C14	1.386 (5)	C18—H18B	0.9800
C13—C17	1.501 (5)	C18—H18C	0.9800
			
Br1^i^—Au1—Br1	180.0	C16—C15—C14	117.0 (4)
Br1^i^—Au1—Br2	90.550 (16)	C16—C15—C18	120.4 (4)
Br1—Au1—Br2	89.450 (16)	C14—C15—C18	122.6 (4)
Br1^i^—Au1—Br2^i^	89.450 (16)	N11—C16—C15	120.1 (4)
Br1—Au1—Br2^i^	90.550 (16)	N11—C16—H16	120.0
Br2—Au1—Br2^i^	180.0	C15—C16—H16	120.0
C16—N11—C12	123.0 (4)	C13—C17—H17A	109.5
C16—N11—H01	120 (3)	C13—C17—H17B	109.5
C12—N11—H01	117 (3)	H17A—C17—H17B	109.5
N11—C12—C13	120.6 (4)	C13—C17—H17C	109.5
N11—C12—H12	119.7	H17A—C17—H17C	109.5
C13—C12—H12	119.7	H17B—C17—H17C	109.5
C12—C13—C14	116.3 (4)	C15—C18—H18A	109.5
C12—C13—C17	121.3 (4)	C15—C18—H18B	109.5
C14—C13—C17	122.4 (4)	H18A—C18—H18B	109.5
C15—C14—C13	123.0 (4)	C15—C18—H18C	109.5
C15—C14—H14	118.5	H18A—C18—H18C	109.5
C13—C14—H14	118.5	H18B—C18—H18C	109.5
			
C16—N11—C12—C13	0.1 (6)	C13—C14—C15—C16	−0.1 (6)
N11—C12—C13—C14	−0.6 (6)	C13—C14—C15—C18	179.2 (4)
N11—C12—C13—C17	178.4 (4)	C12—N11—C16—C15	0.5 (6)
C12—C13—C14—C15	0.7 (6)	C14—C15—C16—N11	−0.4 (6)
C17—C13—C14—C15	−178.4 (4)	C18—C15—C16—N11	−179.7 (4)

Symmetry code: (i) −*x*+1, −*y*+1, −*z*+1.

### Bis(3,5-dimethylpyridinium) tetrabromidoaurate(III) bromide (3a) Hydrogen-bond geometry (Å, º)

**Table:**

*D*—H···*A*	*D*—H	H···*A*	*D*···*A*	*D*—H···*A*
N11—H01···Br3	0.87 (4)	2.42 (4)	3.234 (4)	158 (4)
N11—H01···Br3^ii^	0.87 (4)	2.42 (4)	3.234 (4)	158 (4)
C18—H18*A*···Br1^i^	0.98	2.93	3.862 (4)	159
C16—H16···Br2	0.95	3.00	3.704 (4)	132
C16—H16···Br1^i^	0.95	3.04	3.908 (4)	153

Symmetry codes: (i) −*x*+1, −*y*+1, −*z*+1; (ii) −*x*+1, *y*, −*z*+1/2.

### Bis(3,5-dimethylpyridinium) tetrabromidoaurate(III) bromide (3b) Crystal data

**Table:**

(C_7_H_10_N)_2_[AuBr_4_]Br	*F*(000) = 2976
*M**_r_* = 812.84	*D*_x_ = 2.522 Mg m^−^^3^
Monoclinic, *P*2_1_/*c*	Mo *K*α radiation, λ = 0.71073 Å
*a* = 10.5702 (4) Å	Cell parameters from 10853 reflections
*b* = 24.8303 (11) Å	θ = 2.5–28.6°
*c* = 16.4823 (7) Å	µ = 16.20 mm^−^^1^
β = 98.256 (4)°	*T* = 100 K
*V* = 4281.1 (3) Å^3^	Plate, red
*Z* = 8	0.11 × 0.11 × 0.03 mm

### Bis(3,5-dimethylpyridinium) tetrabromidoaurate(III) bromide (3b) Data collection

**Table:**

Oxford Diffraction Xcalibur, Eos diffractometer	10607 independent reflections
Radiation source: Enhance (Mo) X-ray Source	7996 reflections with *I* > 2σ(*I*)
Graphite monochromator	*R*_int_ = 0.171
Detector resolution: 16.1419 pixels mm^-1^	θ_max_ = 28.3°, θ_min_ = 2.1°
ω scan	*h* = −14→14
Absorption correction: multi-scan (CrysAlisPro; Rigaku OD, 2014)	*k* = −33→33
*T*_min_ = 0.330, *T*_max_ = 1.000	*l* = −21→21
206035 measured reflections	

### Bis(3,5-dimethylpyridinium) tetrabromidoaurate(III) bromide (3b) Refinement

**Table:**

Refinement on *F*^2^	Primary atom site location: structure-invariant direct methods
Least-squares matrix: full	Secondary atom site location: difference Fourier map
*R*[*F*^2^ > 2σ(*F*^2^)] = 0.048	Hydrogen site location: mixed
*wR*(*F*^2^) = 0.079	H atoms treated by a mixture of independent and constrained refinement
*S* = 1.05	*w* = 1/[σ^2^(*F*_o_^2^) + (0.0215*P*)^2^ + 9.196*P*] where *P* = (*F*_o_^2^ + 2*F*_c_^2^)/3
10607 reflections	(Δ/σ)_max_ = 0.002
418 parameters	Δρ_max_ = 1.38 e Å^−^^3^
6 restraints	Δρ_min_ = −1.11 e Å^−^^3^

### Bis(3,5-dimethylpyridinium) tetrabromidoaurate(III) bromide (3b) Fractional atomic coordinates and isotropic or equivalent isotropic displacement parameters (Å^2^)

**Table:**

	*x*	*y*	*z*	*U*_iso_*/*U*_eq_	
Au1	0.17651 (2)	0.74043 (2)	0.23320 (2)	0.01746 (7)	
Au2	0.68504 (3)	0.52616 (2)	0.24220 (2)	0.02044 (7)	
Br1	0.18764 (7)	0.64286 (3)	0.23884 (5)	0.02772 (17)	
Br2	0.22269 (7)	0.74504 (3)	0.38158 (4)	0.02562 (17)	
Br3	0.16695 (7)	0.83786 (3)	0.22820 (4)	0.02294 (16)	
Br4	0.12889 (7)	0.73686 (3)	0.08476 (4)	0.02495 (17)	
Br5	0.69161 (7)	0.55604 (3)	0.10291 (4)	0.02712 (18)	
Br6	0.46937 (7)	0.55908 (3)	0.23300 (5)	0.03003 (19)	
Br7	0.67985 (9)	0.50161 (4)	0.38341 (5)	0.0411 (2)	
Br8	0.90012 (9)	0.49197 (5)	0.25045 (6)	0.0580 (3)	
Br9	0.60080 (7)	0.66533 (3)	0.47762 (4)	0.02621 (18)	
Br10	0.77805 (7)	0.65564 (3)	−0.04715 (4)	0.02819 (18)	
N11	0.8193 (6)	0.7055 (4)	0.1348 (4)	0.039 (2)	
H01	0.809 (7)	0.686 (3)	0.098 (4)	0.026 (12)*	
C12	0.8080 (7)	0.7580 (4)	0.1345 (5)	0.034 (2)	
H12	0.786850	0.776503	0.083927	0.041*	
C13	0.8266 (6)	0.7867 (3)	0.2071 (5)	0.0227 (17)	
C14	0.8541 (6)	0.7562 (3)	0.2783 (4)	0.0223 (16)	
H14	0.866631	0.774100	0.329740	0.027*	
C15	0.8636 (6)	0.7009 (3)	0.2766 (5)	0.0229 (17)	
C16	0.8473 (7)	0.6756 (4)	0.2025 (5)	0.033 (2)	
H16	0.855677	0.637611	0.199016	0.039*	
C17	0.8163 (7)	0.8463 (3)	0.2088 (5)	0.039 (2)	
H17A	0.790662	0.857604	0.261048	0.059*	
H17B	0.752112	0.858278	0.163542	0.059*	
H17C	0.899241	0.862279	0.203010	0.059*	
C18	0.8936 (8)	0.6674 (3)	0.3527 (5)	0.042 (2)	
H18A	0.822559	0.669652	0.384829	0.063*	
H18B	0.971852	0.680792	0.385614	0.063*	
H18C	0.905840	0.629792	0.337475	0.063*	
N21	0.5366 (6)	0.7395 (3)	0.3167 (4)	0.0268 (15)	
H02	0.557 (7)	0.726 (3)	0.359 (3)	0.026 (12)*	
C22	0.5313 (6)	0.7933 (3)	0.3123 (5)	0.0269 (18)	
H22	0.544005	0.814329	0.360942	0.032*	
C23	0.5074 (6)	0.8179 (3)	0.2375 (5)	0.0255 (18)	
C24	0.4907 (6)	0.7857 (3)	0.1686 (5)	0.0254 (17)	
H24	0.474963	0.802369	0.116286	0.030*	
C25	0.4962 (6)	0.7294 (3)	0.1728 (5)	0.0250 (17)	
C26	0.5213 (6)	0.7070 (3)	0.2500 (4)	0.0246 (17)	
H26	0.527691	0.669021	0.256326	0.029*	
C27	0.5015 (8)	0.8777 (3)	0.2308 (6)	0.046 (2)	
H27A	0.559196	0.893683	0.276251	0.069*	
H27B	0.413893	0.889805	0.233056	0.069*	
H27C	0.527645	0.888813	0.178623	0.069*	
C28	0.4776 (7)	0.6941 (3)	0.1000 (5)	0.032 (2)	
H28A	0.386775	0.685079	0.086383	0.048*	
H28B	0.527337	0.660989	0.111656	0.048*	
H28C	0.506350	0.712799	0.053529	0.048*	
N31	0.3241 (6)	0.6113 (3)	0.4696 (4)	0.0209 (14)	
H03	0.382 (5)	0.631 (3)	0.477 (4)	0.026 (12)*	
C32	0.2173 (7)	0.6242 (3)	0.5004 (4)	0.0199 (15)	
H32	0.213309	0.656745	0.530358	0.024*	
C33	0.1146 (6)	0.5905 (3)	0.4886 (4)	0.0181 (15)	
C34	0.1252 (7)	0.5438 (3)	0.4441 (4)	0.0194 (16)	
H34	0.053325	0.520543	0.433022	0.023*	
C35	0.2384 (7)	0.5300 (3)	0.4153 (4)	0.0202 (16)	
C36	0.3362 (6)	0.5661 (3)	0.4282 (4)	0.0206 (16)	
H36	0.413872	0.558818	0.407530	0.025*	
C37	−0.0043 (6)	0.6019 (3)	0.5272 (4)	0.0227 (16)	
H37A	0.007108	0.587905	0.583387	0.034*	
H37B	−0.078121	0.584335	0.495087	0.034*	
H37C	−0.018888	0.640860	0.528164	0.034*	
C38	0.2547 (7)	0.4772 (3)	0.3744 (4)	0.0282 (18)	
H38A	0.324846	0.479882	0.341637	0.042*	
H38B	0.175456	0.467922	0.338528	0.042*	
H38C	0.274386	0.449109	0.416063	0.042*	
N41	1.0513 (6)	0.5978 (3)	−0.0009 (4)	0.0327 (17)	
H04	0.986 (5)	0.611 (3)	−0.017 (4)	0.026 (12)*	
C42	1.1478 (7)	0.6121 (3)	−0.0399 (5)	0.0289 (18)	
H42	1.136176	0.639996	−0.079727	0.035*	
C43	1.2640 (7)	0.5869 (3)	−0.0229 (4)	0.0255 (17)	
C44	1.2734 (7)	0.5462 (3)	0.0343 (4)	0.0256 (17)	
H44	1.352315	0.527519	0.046626	0.031*	
C45	1.1710 (7)	0.5310 (3)	0.0751 (4)	0.0246 (17)	
C46	1.0587 (7)	0.5581 (3)	0.0545 (5)	0.0276 (18)	
H46	0.986021	0.548772	0.079439	0.033*	
C47	1.3737 (7)	0.6011 (3)	−0.0676 (5)	0.0318 (19)	
H47A	1.451094	0.606423	−0.027930	0.048*	
H47B	1.353733	0.634346	−0.098861	0.048*	
H47C	1.387792	0.571824	−0.105148	0.048*	
C48	1.1814 (7)	0.4867 (3)	0.1360 (5)	0.033 (2)	
H48A	1.096272	0.471827	0.138839	0.049*	
H48B	1.217610	0.500749	0.189997	0.049*	
H48C	1.237056	0.458418	0.119619	0.049*	

### Bis(3,5-dimethylpyridinium) tetrabromidoaurate(III) bromide (3b) Atomic displacement parameters (Å^2^)

**Table:**

	*U*^11^	*U*^22^	*U*^33^	*U*^12^	*U*^13^	*U*^23^
Au1	0.01297 (13)	0.02184 (15)	0.01819 (14)	−0.00024 (12)	0.00436 (10)	0.00068 (11)
Au2	0.02262 (15)	0.01984 (15)	0.01828 (14)	0.00061 (12)	0.00095 (11)	0.00175 (12)
Br1	0.0297 (4)	0.0222 (4)	0.0321 (4)	0.0037 (3)	0.0072 (3)	0.0003 (3)
Br2	0.0310 (4)	0.0281 (4)	0.0181 (4)	0.0007 (3)	0.0045 (3)	0.0022 (3)
Br3	0.0192 (4)	0.0224 (4)	0.0266 (4)	−0.0016 (3)	0.0014 (3)	0.0017 (3)
Br4	0.0243 (4)	0.0313 (4)	0.0193 (4)	−0.0024 (3)	0.0033 (3)	−0.0014 (3)
Br5	0.0311 (4)	0.0321 (5)	0.0196 (4)	0.0027 (3)	0.0085 (3)	0.0013 (3)
Br6	0.0226 (4)	0.0441 (5)	0.0243 (4)	0.0038 (4)	0.0065 (3)	0.0065 (4)
Br7	0.0572 (6)	0.0437 (6)	0.0213 (4)	0.0103 (4)	0.0020 (4)	0.0128 (4)
Br8	0.0328 (5)	0.0898 (9)	0.0506 (6)	0.0283 (5)	0.0036 (4)	0.0124 (6)
Br9	0.0209 (4)	0.0374 (5)	0.0202 (4)	−0.0078 (3)	0.0025 (3)	−0.0025 (3)
Br10	0.0286 (4)	0.0359 (5)	0.0195 (4)	0.0115 (4)	0.0015 (3)	0.0057 (3)
N11	0.018 (4)	0.069 (6)	0.031 (4)	0.005 (4)	0.004 (3)	−0.021 (4)
C12	0.017 (4)	0.063 (7)	0.022 (4)	0.009 (4)	0.007 (3)	0.022 (4)
C13	0.008 (3)	0.025 (4)	0.036 (4)	0.005 (3)	0.006 (3)	0.008 (4)
C14	0.013 (4)	0.030 (4)	0.024 (4)	0.005 (3)	0.005 (3)	−0.004 (3)
C15	0.010 (4)	0.029 (4)	0.031 (4)	−0.001 (3)	0.006 (3)	0.006 (3)
C16	0.016 (4)	0.041 (5)	0.041 (5)	0.003 (4)	0.001 (4)	0.000 (4)
C17	0.022 (4)	0.036 (5)	0.063 (6)	0.008 (4)	0.016 (4)	0.020 (5)
C18	0.040 (5)	0.038 (5)	0.050 (6)	0.009 (4)	0.011 (4)	0.020 (4)
N21	0.010 (3)	0.052 (5)	0.020 (3)	0.001 (3)	0.007 (3)	0.005 (3)
C22	0.013 (4)	0.032 (5)	0.038 (5)	−0.005 (3)	0.008 (3)	−0.009 (4)
C23	0.009 (4)	0.032 (5)	0.037 (5)	−0.004 (3)	0.006 (3)	−0.007 (4)
C24	0.012 (4)	0.036 (5)	0.029 (4)	0.003 (3)	0.004 (3)	0.010 (4)
C25	0.010 (4)	0.032 (5)	0.033 (4)	−0.006 (3)	0.004 (3)	−0.003 (4)
C26	0.014 (4)	0.030 (5)	0.029 (4)	0.000 (3)	0.002 (3)	0.001 (4)
C27	0.023 (5)	0.044 (6)	0.072 (7)	−0.009 (4)	0.014 (4)	−0.008 (5)
C28	0.023 (4)	0.045 (5)	0.027 (4)	0.004 (4)	−0.002 (3)	−0.008 (4)
N31	0.018 (3)	0.023 (4)	0.022 (3)	−0.006 (3)	0.002 (3)	−0.001 (3)
C32	0.032 (4)	0.016 (4)	0.011 (3)	0.002 (3)	0.000 (3)	0.002 (3)
C33	0.023 (4)	0.016 (4)	0.015 (3)	−0.002 (3)	0.001 (3)	0.001 (3)
C34	0.020 (4)	0.020 (4)	0.018 (4)	−0.007 (3)	0.004 (3)	0.004 (3)
C35	0.028 (4)	0.017 (4)	0.014 (3)	−0.005 (3)	0.000 (3)	0.002 (3)
C36	0.016 (4)	0.025 (4)	0.022 (4)	0.006 (3)	0.007 (3)	0.013 (3)
C37	0.017 (4)	0.026 (4)	0.026 (4)	−0.003 (3)	0.007 (3)	0.001 (3)
C38	0.034 (5)	0.025 (4)	0.028 (4)	−0.004 (4)	0.013 (3)	0.001 (3)
N41	0.020 (4)	0.032 (4)	0.044 (4)	0.007 (3)	−0.003 (3)	−0.005 (3)
C42	0.032 (5)	0.027 (5)	0.027 (4)	0.002 (4)	−0.002 (4)	−0.003 (3)
C43	0.021 (4)	0.028 (4)	0.027 (4)	0.003 (3)	0.000 (3)	−0.009 (3)
C44	0.031 (4)	0.023 (4)	0.022 (4)	0.006 (3)	0.002 (3)	−0.008 (3)
C45	0.032 (4)	0.024 (4)	0.018 (4)	−0.003 (3)	0.006 (3)	−0.005 (3)
C46	0.019 (4)	0.029 (5)	0.038 (5)	−0.003 (3)	0.015 (3)	−0.007 (4)
C47	0.035 (5)	0.034 (5)	0.026 (4)	0.003 (4)	0.004 (4)	0.005 (4)
C48	0.033 (5)	0.037 (5)	0.030 (4)	0.000 (4)	0.009 (4)	0.004 (4)

### Bis(3,5-dimethylpyridinium) tetrabromidoaurate(III) bromide (3b) Geometric parameters (Å, º)

**Table:**

Au1—Br3	2.4222 (8)	C27—H27C	0.9800
Au1—Br1	2.4268 (8)	C28—H28A	0.9800
Au1—Br2	2.4269 (7)	C28—H28B	0.9800
Au1—Br4	2.4276 (7)	C28—H28C	0.9800
Au2—Br6	2.4060 (8)	N31—C36	1.331 (9)
Au2—Br8	2.4122 (9)	N31—C32	1.342 (9)
Au2—Br7	2.4142 (8)	N31—H03	0.77 (4)
Au2—Br5	2.4232 (8)	C32—C33	1.364 (9)
N11—C12	1.310 (11)	C32—H32	0.9500
N11—C16	1.337 (11)	C33—C34	1.385 (9)
N11—H01	0.77 (4)	C33—C37	1.515 (9)
C12—C13	1.383 (11)	C34—C35	1.392 (9)
C12—H12	0.9500	C34—H34	0.9500
C13—C14	1.392 (10)	C35—C36	1.362 (9)
C13—C17	1.483 (10)	C35—C38	1.496 (10)
C14—C15	1.377 (10)	C36—H36	0.9500
C14—H14	0.9500	C37—H37A	0.9800
C15—C16	1.362 (10)	C37—H37B	0.9800
C15—C18	1.502 (10)	C37—H37C	0.9800
C16—H16	0.9500	C38—H38A	0.9800
C17—H17A	0.9800	C38—H38B	0.9800
C17—H17B	0.9800	C38—H38C	0.9800
C17—H17C	0.9800	N41—C42	1.329 (10)
C18—H18A	0.9800	N41—C46	1.340 (10)
C18—H18B	0.9800	N41—H04	0.77 (4)
C18—H18C	0.9800	C42—C43	1.372 (10)
N21—C22	1.339 (10)	C42—H42	0.9500
N21—C26	1.356 (9)	C43—C44	1.375 (10)
N21—H02	0.78 (4)	C43—C47	1.503 (10)
C22—C23	1.365 (10)	C44—C45	1.405 (10)
C22—H22	0.9500	C44—H44	0.9500
C23—C24	1.379 (10)	C45—C46	1.364 (10)
C23—C27	1.490 (11)	C45—C48	1.483 (10)
C24—C25	1.400 (10)	C46—H46	0.9500
C24—H24	0.9500	C47—H47A	0.9800
C25—C26	1.379 (10)	C47—H47B	0.9800
C25—C28	1.476 (10)	C47—H47C	0.9800
C26—H26	0.9500	C48—H48A	0.9800
C27—H27A	0.9800	C48—H48B	0.9800
C27—H27B	0.9800	C48—H48C	0.9800
			
Br3—Au1—Br1	179.57 (3)	C25—C28—H28A	109.5
Br3—Au1—Br2	89.34 (3)	C25—C28—H28B	109.5
Br1—Au1—Br2	90.39 (3)	H28A—C28—H28B	109.5
Br3—Au1—Br4	90.04 (3)	C25—C28—H28C	109.5
Br1—Au1—Br4	90.23 (3)	H28A—C28—H28C	109.5
Br2—Au1—Br4	179.30 (3)	H28B—C28—H28C	109.5
Br6—Au2—Br8	179.17 (4)	C36—N31—C32	123.0 (6)
Br6—Au2—Br7	89.66 (3)	C36—N31—H03	118 (6)
Br8—Au2—Br7	90.49 (3)	C32—N31—H03	119 (6)
Br6—Au2—Br5	89.54 (3)	N31—C32—C33	119.7 (7)
Br8—Au2—Br5	90.35 (3)	N31—C32—H32	120.2
Br7—Au2—Br5	176.77 (3)	C33—C32—H32	120.2
C12—N11—C16	124.3 (7)	C32—C33—C34	117.9 (6)
C12—N11—H01	129 (6)	C32—C33—C37	121.1 (6)
C16—N11—H01	106 (6)	C34—C33—C37	121.0 (6)
N11—C12—C13	120.6 (7)	C33—C34—C35	121.7 (6)
N11—C12—H12	119.7	C33—C34—H34	119.2
C13—C12—H12	119.7	C35—C34—H34	119.2
C12—C13—C14	115.7 (7)	C36—C35—C34	117.1 (7)
C12—C13—C17	122.0 (7)	C36—C35—C38	120.9 (7)
C14—C13—C17	122.3 (7)	C34—C35—C38	122.0 (6)
C15—C14—C13	122.2 (7)	N31—C36—C35	120.6 (7)
C15—C14—H14	118.9	N31—C36—H36	119.7
C13—C14—H14	118.9	C35—C36—H36	119.7
C16—C15—C14	118.6 (7)	C33—C37—H37A	109.5
C16—C15—C18	118.5 (8)	C33—C37—H37B	109.5
C14—C15—C18	122.9 (7)	H37A—C37—H37B	109.5
N11—C16—C15	118.5 (8)	C33—C37—H37C	109.5
N11—C16—H16	120.8	H37A—C37—H37C	109.5
C15—C16—H16	120.8	H37B—C37—H37C	109.5
C13—C17—H17A	109.5	C35—C38—H38A	109.5
C13—C17—H17B	109.5	C35—C38—H38B	109.5
H17A—C17—H17B	109.5	H38A—C38—H38B	109.5
C13—C17—H17C	109.5	C35—C38—H38C	109.5
H17A—C17—H17C	109.5	H38A—C38—H38C	109.5
H17B—C17—H17C	109.5	H38B—C38—H38C	109.5
C15—C18—H18A	109.5	C42—N41—C46	123.6 (7)
C15—C18—H18B	109.5	C42—N41—H04	116 (6)
H18A—C18—H18B	109.5	C46—N41—H04	120 (6)
C15—C18—H18C	109.5	N41—C42—C43	120.4 (8)
H18A—C18—H18C	109.5	N41—C42—H42	119.8
H18B—C18—H18C	109.5	C43—C42—H42	119.8
C22—N21—C26	123.5 (7)	C42—C43—C44	116.8 (7)
C22—N21—H02	119 (6)	C42—C43—C47	121.6 (7)
C26—N21—H02	117 (6)	C44—C43—C47	121.6 (7)
N21—C22—C23	119.7 (7)	C43—C44—C45	122.7 (7)
N21—C22—H22	120.2	C43—C44—H44	118.7
C23—C22—H22	120.2	C45—C44—H44	118.7
C22—C23—C24	118.0 (7)	C46—C45—C44	116.9 (7)
C22—C23—C27	120.8 (7)	C46—C45—C48	120.7 (7)
C24—C23—C27	121.1 (7)	C44—C45—C48	122.4 (7)
C23—C24—C25	122.6 (7)	N41—C46—C45	119.7 (7)
C23—C24—H24	118.7	N41—C46—H46	120.2
C25—C24—H24	118.7	C45—C46—H46	120.2
C26—C25—C24	116.7 (7)	C43—C47—H47A	109.5
C26—C25—C28	119.7 (7)	C43—C47—H47B	109.5
C24—C25—C28	123.6 (7)	H47A—C47—H47B	109.5
N21—C26—C25	119.5 (7)	C43—C47—H47C	109.5
N21—C26—H26	120.2	H47A—C47—H47C	109.5
C25—C26—H26	120.2	H47B—C47—H47C	109.5
C23—C27—H27A	109.5	C45—C48—H48A	109.5
C23—C27—H27B	109.5	C45—C48—H48B	109.5
H27A—C27—H27B	109.5	H48A—C48—H48B	109.5
C23—C27—H27C	109.5	C45—C48—H48C	109.5
H27A—C27—H27C	109.5	H48A—C48—H48C	109.5
H27B—C27—H27C	109.5	H48B—C48—H48C	109.5
			
C16—N11—C12—C13	0.8 (12)	C36—N31—C32—C33	−1.1 (10)
N11—C12—C13—C14	−1.6 (10)	N31—C32—C33—C34	−0.3 (10)
N11—C12—C13—C17	179.1 (7)	N31—C32—C33—C37	176.0 (6)
C12—C13—C14—C15	0.7 (10)	C32—C33—C34—C35	2.9 (10)
C17—C13—C14—C15	−180.0 (6)	C37—C33—C34—C35	−173.4 (6)
C13—C14—C15—C16	0.9 (10)	C33—C34—C35—C36	−4.0 (10)
C13—C14—C15—C18	179.8 (7)	C33—C34—C35—C38	174.0 (6)
C12—N11—C16—C15	1.0 (12)	C32—N31—C36—C35	−0.1 (10)
C14—C15—C16—N11	−1.8 (10)	C34—C35—C36—N31	2.6 (10)
C18—C15—C16—N11	179.3 (7)	C38—C35—C36—N31	−175.4 (6)
C26—N21—C22—C23	1.0 (10)	C46—N41—C42—C43	−1.8 (12)
N21—C22—C23—C24	−0.6 (10)	N41—C42—C43—C44	1.3 (11)
N21—C22—C23—C27	−179.7 (6)	N41—C42—C43—C47	178.3 (7)
C22—C23—C24—C25	0.6 (10)	C42—C43—C44—C45	−1.0 (11)
C27—C23—C24—C25	179.7 (6)	C47—C43—C44—C45	−178.0 (7)
C23—C24—C25—C26	−0.9 (10)	C43—C44—C45—C46	1.2 (11)
C23—C24—C25—C28	179.4 (7)	C43—C44—C45—C48	179.4 (7)
C22—N21—C26—C25	−1.3 (10)	C42—N41—C46—C45	1.9 (12)
C24—C25—C26—N21	1.2 (10)	C44—C45—C46—N41	−1.5 (11)
C28—C25—C26—N21	−179.1 (6)	C48—C45—C46—N41	−179.8 (7)

### Bis(3,5-dimethylpyridinium) tetrabromidoaurate(III) bromide (3b) Hydrogen-bond geometry (Å, º)

**Table:**

*D*—H···*A*	*D*—H	H···*A*	*D*···*A*	*D*—H···*A*
N11—H01···Br10	0.77 (4)	2.49 (5)	3.215 (7)	157 (8)
N21—H02···Br9	0.78 (4)	2.46 (4)	3.220 (7)	168 (8)
N31—H03···Br9	0.77 (4)	2.47 (5)	3.203 (6)	159 (7)
N41—H04···Br10	0.77 (4)	2.45 (4)	3.218 (6)	172 (8)
C12—H12···Br9^i^	0.95	2.83	3.672 (8)	148
C16—H16···Br5	0.95	2.97	3.666 (8)	131
C22—H22···Br10^ii^	0.95	2.81	3.470 (7)	127
C26—H26···Br6	0.95	2.81	3.718 (8)	160
C32—H32···Br4^ii^	0.95	2.97	3.882 (7)	162
C36—H36···Br6	0.95	3.02	3.697 (7)	130
C42—H42···Br2^iii^	0.95	3.09	3.896 (8)	143
C17—H17*C*···Br3^iv^	0.98	2.87	3.680 (7)	141
C27—H27*B*···Br3	0.98	2.90	3.666 (8)	136
C28—H28*A*···Br4	0.98	3.01	3.809 (7)	140
C48—H48*B*···Br6^iv^	0.98	3.02	3.694 (8)	127
C48—H48*A*···Br8	0.98	3.00	3.744 (8)	133
C18—H18*A*···Br9	0.98	2.98	3.955 (8)	174
C24—H24···Br9^i^	0.95	2.92	3.714 (7)	142
C14—H14···Br10^ii^	0.95	2.93	3.792 (7)	152

Symmetry codes: (i) *x*, −*y*+3/2, *z*−1/2; (ii) *x*, −*y*+3/2, *z*+1/2; (iii) *x*+1, −*y*+3/2, *z*−1/2; (iv) *x*+1, *y*, *z*.

### Tris(3,5-dimethylpyridinium) bis[tetrabromidoaurate(III)] bromide (4) Crystal data

**Table:**

(C_7_H_10_N)_3_[AuBr_4_]_2_Br	*Z* = 2
*M**_r_* = 1437.60	*F*(000) = 1300
Triclinic, *P*1	*D*_x_ = 2.794 Mg m^−^^3^
*a* = 8.1261 (4) Å	Mo *K*α radiation, λ = 0.71073 Å
*b* = 12.0375 (5) Å	Cell parameters from 14875 reflections
*c* = 18.0076 (11) Å	θ = 2.6–28.3°
α = 90.532 (4)°	µ = 19.12 mm^−^^1^
β = 94.151 (5)°	*T* = 100 K
γ = 103.359 (4)°	Block, red
*V* = 1708.68 (15) Å^3^	0.15 × 0.10 × 0.05 mm

### Tris(3,5-dimethylpyridinium) bis[tetrabromidoaurate(III)] bromide (4) Data collection

**Table:**

Oxford Diffraction Xcalibur, Eos diffractometer	8153 independent reflections
Radiation source: fine-focus sealed X-ray tube	6751 reflections with *I* > 2σ(*I*)
Detector resolution: 16.1419 pixels mm^-1^	*R*_int_ = 0.088
ω scans	θ_max_ = 27.9°, θ_min_ = 2.3°
Absorption correction: multi-scan (CrysAlisPro; Rigaku OD, 2014)	*h* = −10→10
*T*_min_ = 0.256, *T*_max_ = 1.000	*k* = −15→15
86859 measured reflections	*l* = −23→23

### Tris(3,5-dimethylpyridinium) bis[tetrabromidoaurate(III)] bromide (4) Refinement

**Table:**

Refinement on *F*^2^	Secondary atom site location: difference Fourier map
Least-squares matrix: full	Hydrogen site location: mixed
*R*[*F*^2^ > 2σ(*F*^2^)] = 0.031	H atoms treated by a mixture of independent and constrained refinement
*wR*(*F*^2^) = 0.053	*w* = 1/[σ^2^(*F*_o_^2^) + (0.0142*P*)^2^ + 0.0753*P*] where *P* = (*F*_o_^2^ + 2*F*_c_^2^)/3
*S* = 1.03	(Δ/σ)_max_ = 0.001
8153 reflections	Δρ_max_ = 1.33 e Å^−^^3^
336 parameters	Δρ_min_ = −1.16 e Å^−^^3^
3 restraints	Extinction correction: *SHELXL2019/3* (Sheldrick, 2015), *F*_c_^*^ = *kF*_c_[1 + 0.001*xF*_c_^2^λ^3^/sin(2θ)]^-1/4^
Primary atom site location: structure-invariant direct methods	Extinction coefficient: 0.00037 (2)

### Tris(3,5-dimethylpyridinium) bis[tetrabromidoaurate(III)] bromide (4) Fractional atomic coordinates and isotropic or equivalent isotropic displacement parameters (Å^2^)

**Table:**

	*x*	*y*	*z*	*U*_iso_*/*U*_eq_	
Au1	0.65381 (2)	0.06365 (2)	0.22454 (2)	0.01300 (5)	
Br1	0.59814 (7)	−0.03830 (5)	0.10520 (3)	0.02484 (13)	
Br2	0.35236 (6)	0.02537 (5)	0.23924 (3)	0.01974 (12)	
Br3	0.71207 (7)	0.16783 (4)	0.34277 (3)	0.02142 (12)	
Br4	0.95626 (6)	0.09723 (5)	0.21069 (3)	0.02221 (13)	
Au2	0.500000	0.500000	0.000000	0.01212 (7)	
Br5	0.20154 (6)	0.47887 (5)	0.01682 (3)	0.02164 (13)	
Br6	0.57077 (7)	0.62243 (4)	0.11030 (3)	0.02101 (12)	
Au3	1.000000	0.500000	0.500000	0.01305 (7)	
Br7	0.74042 (6)	0.50067 (4)	0.42418 (3)	0.01793 (12)	
Br8	0.91605 (7)	0.29495 (4)	0.51063 (3)	0.01917 (12)	
Br9	0.30373 (7)	0.50391 (4)	0.34428 (3)	0.02044 (12)	
N11	0.4858 (6)	0.3775 (4)	0.2276 (3)	0.0215 (11)	
H01	0.429 (6)	0.399 (4)	0.259 (3)	0.025 (10)*	
C12	0.6523 (7)	0.4001 (4)	0.2250 (3)	0.0211 (13)	
H12	0.723425	0.446315	0.263163	0.025*	
C13	0.7233 (6)	0.3573 (4)	0.1674 (3)	0.0185 (12)	
C14	0.6146 (6)	0.2883 (4)	0.1148 (3)	0.0164 (12)	
H14	0.660941	0.257362	0.074618	0.020*	
C15	0.4380 (7)	0.2619 (4)	0.1183 (3)	0.0166 (12)	
C16	0.3786 (7)	0.3113 (4)	0.1769 (3)	0.0192 (12)	
H16	0.259903	0.298079	0.181345	0.023*	
C17	0.9113 (7)	0.3875 (5)	0.1625 (3)	0.0285 (14)	
H17A	0.945485	0.463845	0.142020	0.043*	
H17B	0.943382	0.331629	0.129942	0.043*	
H17C	0.968229	0.386937	0.212326	0.043*	
C18	0.3195 (7)	0.1840 (5)	0.0630 (3)	0.0255 (13)	
H18A	0.203791	0.170674	0.078772	0.038*	
H18B	0.353164	0.111150	0.059787	0.038*	
H18C	0.323679	0.219177	0.014081	0.038*	
N21	0.3416 (6)	0.2832 (4)	0.4445 (3)	0.0203 (11)	
H02	0.356 (6)	0.350 (3)	0.428 (3)	0.025 (10)*	
C22	0.2263 (6)	0.1964 (4)	0.4105 (3)	0.0174 (12)	
H22	0.159610	0.209640	0.367385	0.021*	
C23	0.2045 (6)	0.0874 (4)	0.4383 (3)	0.0167 (12)	
C24	0.3074 (6)	0.0743 (4)	0.5006 (3)	0.0160 (12)	
H24	0.295801	0.000183	0.520392	0.019*	
C25	0.4266 (6)	0.1639 (4)	0.5354 (3)	0.0161 (12)	
C26	0.4403 (6)	0.2708 (4)	0.5049 (3)	0.0174 (12)	
H26	0.520057	0.335132	0.526903	0.021*	
C27	0.0734 (6)	−0.0090 (4)	0.4009 (3)	0.0193 (12)	
H27A	0.036624	−0.068223	0.437125	0.029*	
H27B	0.122193	−0.041442	0.359995	0.029*	
H27C	−0.024200	0.019433	0.381184	0.029*	
C28	0.5347 (6)	0.1493 (5)	0.6038 (3)	0.0216 (13)	
H28A	0.638229	0.129084	0.589313	0.032*	
H28B	0.471830	0.088413	0.633586	0.032*	
H28C	0.564945	0.220958	0.633285	0.032*	
N31	0.1585 (6)	0.6752 (4)	0.2327 (3)	0.0245 (12)	
H03	0.226 (6)	0.635 (4)	0.246 (3)	0.025 (10)*	
C32	0.1847 (6)	0.7178 (4)	0.1647 (3)	0.0202 (12)	
H32	0.275060	0.703419	0.138306	0.024*	
C33	0.0792 (6)	0.7824 (4)	0.1334 (3)	0.0184 (12)	
C34	−0.0517 (6)	0.7978 (4)	0.1741 (3)	0.0177 (12)	
H34	−0.127847	0.840309	0.153234	0.021*	
C35	−0.0765 (6)	0.7535 (4)	0.2444 (3)	0.0190 (12)	
C36	0.0344 (7)	0.6913 (4)	0.2731 (3)	0.0219 (13)	
H36	0.022628	0.660189	0.321244	0.026*	
C37	0.1043 (7)	0.8295 (5)	0.0574 (3)	0.0289 (14)	
H37A	0.052303	0.770105	0.019695	0.043*	
H37B	0.225957	0.854884	0.051112	0.043*	
H37C	0.051069	0.894466	0.051506	0.043*	
C38	−0.2200 (7)	0.7705 (5)	0.2887 (3)	0.0292 (14)	
H38A	−0.325074	0.716238	0.270633	0.044*	
H38B	−0.234259	0.848682	0.282854	0.044*	
H38C	−0.193679	0.757677	0.341473	0.044*	

### Tris(3,5-dimethylpyridinium) bis[tetrabromidoaurate(III)] bromide (4) Atomic displacement parameters (Å^2^)

**Table:**

	*U*^11^	*U*^22^	*U*^33^	*U*^12^	*U*^13^	*U*^23^
Au1	0.01294 (10)	0.01275 (10)	0.01356 (12)	0.00393 (7)	−0.00054 (8)	0.00104 (8)
Br1	0.0243 (3)	0.0316 (3)	0.0207 (3)	0.0132 (2)	−0.0046 (2)	−0.0110 (3)
Br2	0.0139 (3)	0.0251 (3)	0.0207 (3)	0.0049 (2)	0.0020 (2)	0.0059 (2)
Br3	0.0282 (3)	0.0199 (3)	0.0151 (3)	0.0048 (2)	−0.0018 (2)	−0.0021 (2)
Br4	0.0134 (3)	0.0242 (3)	0.0287 (4)	0.0037 (2)	0.0017 (2)	0.0019 (2)
Au2	0.00975 (13)	0.01180 (13)	0.01529 (16)	0.00318 (10)	0.00171 (11)	0.00245 (11)
Br5	0.0106 (2)	0.0274 (3)	0.0278 (4)	0.0055 (2)	0.0041 (2)	0.0023 (3)
Br6	0.0210 (3)	0.0205 (3)	0.0211 (3)	0.0047 (2)	0.0000 (2)	−0.0043 (2)
Au3	0.01652 (14)	0.01264 (14)	0.00981 (16)	0.00322 (11)	0.00055 (11)	−0.00053 (11)
Br7	0.0190 (3)	0.0195 (3)	0.0147 (3)	0.0043 (2)	−0.0020 (2)	0.0004 (2)
Br8	0.0233 (3)	0.0131 (2)	0.0203 (3)	0.0027 (2)	0.0007 (2)	0.0002 (2)
Br9	0.0302 (3)	0.0213 (3)	0.0147 (3)	0.0157 (2)	0.0022 (2)	0.0023 (2)
N11	0.030 (3)	0.018 (2)	0.020 (3)	0.010 (2)	0.013 (2)	0.002 (2)
C12	0.030 (3)	0.018 (3)	0.016 (3)	0.005 (2)	0.004 (3)	0.004 (2)
C13	0.021 (3)	0.013 (3)	0.022 (3)	0.004 (2)	0.002 (2)	0.007 (2)
C14	0.023 (3)	0.015 (3)	0.015 (3)	0.009 (2)	0.008 (2)	0.006 (2)
C15	0.026 (3)	0.016 (3)	0.008 (3)	0.005 (2)	0.000 (2)	0.007 (2)
C16	0.026 (3)	0.019 (3)	0.016 (3)	0.008 (2)	0.007 (2)	0.005 (2)
C17	0.024 (3)	0.029 (3)	0.031 (4)	0.003 (3)	0.004 (3)	0.008 (3)
C18	0.031 (3)	0.024 (3)	0.021 (4)	0.006 (3)	0.000 (3)	0.002 (3)
N21	0.028 (3)	0.014 (2)	0.022 (3)	0.008 (2)	0.008 (2)	0.010 (2)
C22	0.020 (3)	0.019 (3)	0.016 (3)	0.007 (2)	0.004 (2)	0.002 (2)
C23	0.016 (3)	0.019 (3)	0.018 (3)	0.007 (2)	0.010 (2)	0.000 (2)
C24	0.017 (3)	0.017 (3)	0.016 (3)	0.005 (2)	0.007 (2)	0.004 (2)
C25	0.016 (3)	0.021 (3)	0.015 (3)	0.009 (2)	0.008 (2)	0.003 (2)
C26	0.018 (3)	0.015 (3)	0.019 (3)	0.004 (2)	0.007 (2)	0.000 (2)
C27	0.015 (3)	0.021 (3)	0.020 (3)	0.002 (2)	0.001 (2)	0.000 (2)
C28	0.023 (3)	0.023 (3)	0.021 (3)	0.009 (2)	0.001 (2)	0.005 (2)
N31	0.023 (3)	0.021 (3)	0.032 (3)	0.013 (2)	−0.011 (2)	0.000 (2)
C32	0.013 (3)	0.022 (3)	0.026 (4)	0.005 (2)	0.001 (2)	−0.006 (3)
C33	0.017 (3)	0.015 (3)	0.020 (3)	−0.002 (2)	−0.003 (2)	−0.007 (2)
C34	0.010 (3)	0.014 (3)	0.029 (4)	0.004 (2)	−0.006 (2)	−0.001 (2)
C35	0.020 (3)	0.011 (3)	0.024 (3)	0.001 (2)	−0.005 (2)	−0.001 (2)
C36	0.028 (3)	0.016 (3)	0.017 (3)	−0.002 (2)	−0.004 (2)	0.001 (2)
C37	0.028 (3)	0.031 (3)	0.026 (4)	0.002 (3)	0.002 (3)	0.003 (3)
C38	0.029 (3)	0.028 (3)	0.030 (4)	0.003 (3)	0.004 (3)	−0.003 (3)

### Tris(3,5-dimethylpyridinium) bis[tetrabromidoaurate(III)] bromide (4) Geometric parameters (Å, º)

**Table:**

Au1—Br2	2.4203 (5)	C22—C23	1.386 (7)
Au1—Br3	2.4206 (6)	C22—H22	0.9500
Au1—Br1	2.4255 (6)	C23—C24	1.383 (7)
Au1—Br4	2.4285 (5)	C23—C27	1.499 (7)
Au2—Br6^i^	2.4159 (6)	C24—C25	1.383 (7)
Au2—Br6	2.4159 (6)	C24—H24	0.9500
Au2—Br5	2.4210 (5)	C25—C26	1.388 (7)
Au2—Br5^i^	2.4210 (5)	C25—C28	1.496 (7)
Au3—Br8	2.4174 (5)	C26—H26	0.9500
Au3—Br8^ii^	2.4174 (5)	C27—H27A	0.9800
Au3—Br7^ii^	2.4300 (5)	C27—H27B	0.9800
Au3—Br7	2.4300 (5)	C27—H27C	0.9800
N11—C12	1.321 (7)	C28—H28A	0.9800
N11—C16	1.333 (7)	C28—H28B	0.9800
N11—H01	0.83 (3)	C28—H28C	0.9800
C12—C13	1.374 (7)	N31—C36	1.334 (7)
C12—H12	0.9500	N31—C32	1.345 (7)
C13—C14	1.376 (7)	N31—H03	0.83 (3)
C13—C17	1.496 (7)	C32—C33	1.379 (7)
C14—C15	1.403 (7)	C32—H32	0.9500
C14—H14	0.9500	C33—C34	1.381 (7)
C15—C16	1.378 (7)	C33—C37	1.497 (7)
C15—C18	1.491 (7)	C34—C35	1.387 (7)
C16—H16	0.9500	C34—H34	0.9500
C17—H17A	0.9800	C35—C36	1.375 (7)
C17—H17B	0.9800	C35—C38	1.510 (7)
C17—H17C	0.9800	C36—H36	0.9500
C18—H18A	0.9800	C37—H37A	0.9800
C18—H18B	0.9800	C37—H37B	0.9800
C18—H18C	0.9800	C37—H37C	0.9800
N21—C26	1.333 (7)	C38—H38A	0.9800
N21—C22	1.341 (7)	C38—H38B	0.9800
N21—H02	0.84 (3)	C38—H38C	0.9800
			
Br2—Au1—Br3	90.39 (2)	C24—C23—C22	116.9 (5)
Br2—Au1—Br1	90.16 (2)	C24—C23—C27	123.3 (5)
Br3—Au1—Br1	179.13 (2)	C22—C23—C27	119.8 (5)
Br2—Au1—Br4	178.57 (2)	C25—C24—C23	123.0 (5)
Br3—Au1—Br4	89.93 (2)	C25—C24—H24	118.5
Br1—Au1—Br4	89.55 (2)	C23—C24—H24	118.5
Br6^i^—Au2—Br6	180.00 (3)	C24—C25—C26	116.9 (5)
Br6^i^—Au2—Br5	90.278 (19)	C24—C25—C28	122.7 (5)
Br6—Au2—Br5	89.722 (19)	C26—C25—C28	120.5 (5)
Br6^i^—Au2—Br5^i^	89.721 (19)	N21—C26—C25	120.0 (5)
Br6—Au2—Br5^i^	90.279 (19)	N21—C26—H26	120.0
Br5—Au2—Br5^i^	180.0	C25—C26—H26	120.0
Br8—Au3—Br8^ii^	180.0	C23—C27—H27A	109.5
Br8—Au3—Br7^ii^	88.897 (18)	C23—C27—H27B	109.5
Br8^ii^—Au3—Br7^ii^	91.104 (18)	H27A—C27—H27B	109.5
Br8—Au3—Br7	91.104 (18)	C23—C27—H27C	109.5
Br8^ii^—Au3—Br7	88.895 (18)	H27A—C27—H27C	109.5
Br7^ii^—Au3—Br7	180.0	H27B—C27—H27C	109.5
C12—N11—C16	123.1 (5)	C25—C28—H28A	109.5
C12—N11—H01	129 (4)	C25—C28—H28B	109.5
C16—N11—H01	108 (4)	H28A—C28—H28B	109.5
N11—C12—C13	120.4 (5)	C25—C28—H28C	109.5
N11—C12—H12	119.8	H28A—C28—H28C	109.5
C13—C12—H12	119.8	H28B—C28—H28C	109.5
C12—C13—C14	117.3 (5)	C36—N31—C32	123.8 (5)
C12—C13—C17	120.4 (5)	C36—N31—H03	124 (4)
C14—C13—C17	122.2 (5)	C32—N31—H03	112 (4)
C13—C14—C15	122.4 (5)	N31—C32—C33	119.5 (5)
C13—C14—H14	118.8	N31—C32—H32	120.2
C15—C14—H14	118.8	C33—C32—H32	120.2
C16—C15—C14	116.0 (5)	C32—C33—C34	117.1 (5)
C16—C15—C18	121.2 (5)	C32—C33—C37	120.6 (5)
C14—C15—C18	122.8 (5)	C34—C33—C37	122.3 (5)
N11—C16—C15	120.7 (5)	C33—C34—C35	122.6 (5)
N11—C16—H16	119.6	C33—C34—H34	118.7
C15—C16—H16	119.6	C35—C34—H34	118.7
C13—C17—H17A	109.5	C36—C35—C34	117.6 (5)
C13—C17—H17B	109.5	C36—C35—C38	120.0 (5)
H17A—C17—H17B	109.5	C34—C35—C38	122.4 (5)
C13—C17—H17C	109.5	N31—C36—C35	119.3 (5)
H17A—C17—H17C	109.5	N31—C36—H36	120.3
H17B—C17—H17C	109.5	C35—C36—H36	120.3
C15—C18—H18A	109.5	C33—C37—H37A	109.5
C15—C18—H18B	109.5	C33—C37—H37B	109.5
H18A—C18—H18B	109.5	H37A—C37—H37B	109.5
C15—C18—H18C	109.5	C33—C37—H37C	109.5
H18A—C18—H18C	109.5	H37A—C37—H37C	109.5
H18B—C18—H18C	109.5	H37B—C37—H37C	109.5
C26—N21—C22	123.3 (5)	C35—C38—H38A	109.5
C26—N21—H02	116 (4)	C35—C38—H38B	109.5
C22—N21—H02	121 (4)	H38A—C38—H38B	109.5
N21—C22—C23	119.9 (5)	C35—C38—H38C	109.5
N21—C22—H22	120.1	H38A—C38—H38C	109.5
C23—C22—H22	120.1	H38B—C38—H38C	109.5
			
C16—N11—C12—C13	1.6 (8)	C23—C24—C25—C26	−0.2 (8)
N11—C12—C13—C14	−1.8 (7)	C23—C24—C25—C28	178.6 (5)
N11—C12—C13—C17	177.0 (5)	C22—N21—C26—C25	0.1 (8)
C12—C13—C14—C15	0.3 (7)	C24—C25—C26—N21	−0.2 (7)
C17—C13—C14—C15	−178.5 (5)	C28—C25—C26—N21	−179.0 (5)
C13—C14—C15—C16	1.4 (7)	C36—N31—C32—C33	0.2 (8)
C13—C14—C15—C18	−177.9 (5)	N31—C32—C33—C34	−1.4 (7)
C12—N11—C16—C15	0.3 (8)	N31—C32—C33—C37	−179.2 (5)
C14—C15—C16—N11	−1.7 (7)	C32—C33—C34—C35	1.6 (8)
C18—C15—C16—N11	177.6 (5)	C37—C33—C34—C35	179.4 (5)
C26—N21—C22—C23	0.5 (8)	C33—C34—C35—C36	−0.6 (8)
N21—C22—C23—C24	−0.8 (7)	C33—C34—C35—C38	180.0 (5)
N21—C22—C23—C27	179.0 (5)	C32—N31—C36—C35	0.9 (8)
C22—C23—C24—C25	0.7 (8)	C34—C35—C36—N31	−0.7 (8)
C27—C23—C24—C25	−179.1 (5)	C38—C35—C36—N31	178.7 (5)

Symmetry codes: (i) −*x*+1, −*y*+1, −*z*; (ii) −*x*+2, −*y*+1, −*z*+1.

### Tris(3,5-dimethylpyridinium) bis[tetrabromidoaurate(III)] bromide (4) Hydrogen-bond geometry (Å, º)

**Table:**

*D*—H···*A*	*D*—H	H···*A*	*D*···*A*	*D*—H···*A*
N11—H01···Br9	0.83 (3)	2.40 (3)	3.217 (5)	166 (5)
N21—H02···Br9	0.84 (3)	2.50 (4)	3.286 (4)	157 (5)
N31—H03···Br9	0.83 (3)	2.53 (4)	3.246 (5)	145 (5)
C12—H12···Br7	0.95	2.95	3.740 (6)	141
C32—H32···Br6	0.95	2.87	3.769 (5)	159
C18—H18*C*···Br6^i^	0.98	3.00	3.942 (6)	162
C27—H27*A*···Br8^iii^	0.98	2.99	3.822 (5)	143
C34—H34···Br1^iv^	0.95	3.01	3.957 (5)	172

Symmetry codes: (i) −*x*+1, −*y*+1, −*z*; (iii) −*x*+1, −*y*, −*z*+1; (iv) *x*−1, *y*+1, *z*.
